# Difluorenoheteroles: topological control of π conjugation in diradicaloids and mixed-valence radical ions†

**DOI:** 10.1039/d4sc02459a

**Published:** 2024-05-23

**Authors:** Bibek Prajapati, Tendai Kwenda, Tadeusz Lis, Piotr J. Chmielewski, Carlos J. Gómez-García, Marcin A. Majewski, Marcin Stępień

**Affiliations:** a Wydział Chemii, Uniwersytet Wrocławski ul. F. Joliot-Curie 14 50-383 Wrocław Poland marcin.stepien@uwr.edu.pl; b Departamento de Química Inorgánica, Universidad de Valencia Dr Moliner 50 46100 Burjasot Spain

## Abstract

Two families of difluorenoheterole diradicaloids were synthesized, featuring isomeric ring systems with distinct conjugation topologies. The two types of difluorenoheteroles contain, respectively, a Chichibabin-like motif (CH) and a newly introduced heteroatom-linked triphenylmethyl dyad (TD-X). Combined experimental and theoretical investigations show that the TD-X systems have reduced quinoidal character but the interaction between formal spin centers is sufficiently strong to ensure a singlet ground state. The singlet–triplet energy gaps in the TD-X difluorenoheteroles are strongly affected by the heterocyclic ring, with values of −4.3 and −0.7 kcal mol^−1^ determined for the pyrrole- and thiophene-containing analogues, respectively. In cyclic voltammetry experiments, the TD-X systems show diminished energy gaps and superior reversibility in comparison with their CH counterparts. The radical anions and cations obtained from these diradicaloids show extremely red-shifted bands, occasionally with *λ*_max_ > 3500 nm. Computational studies show that some of these ions adopt distonic structures and may be characterized as class-II mixed-valence species.

## Abstract 2

Linking and fusion of triphenylmethyl radicals produces isomeric difluorenoheterole diradicaloids, with heteroatom-dependent paths for spin–spin interactions.

## Graphical abstract

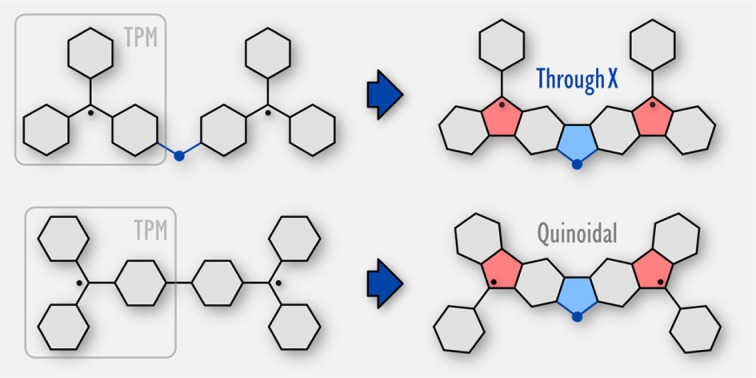

## Introduction

**Chart 1:**
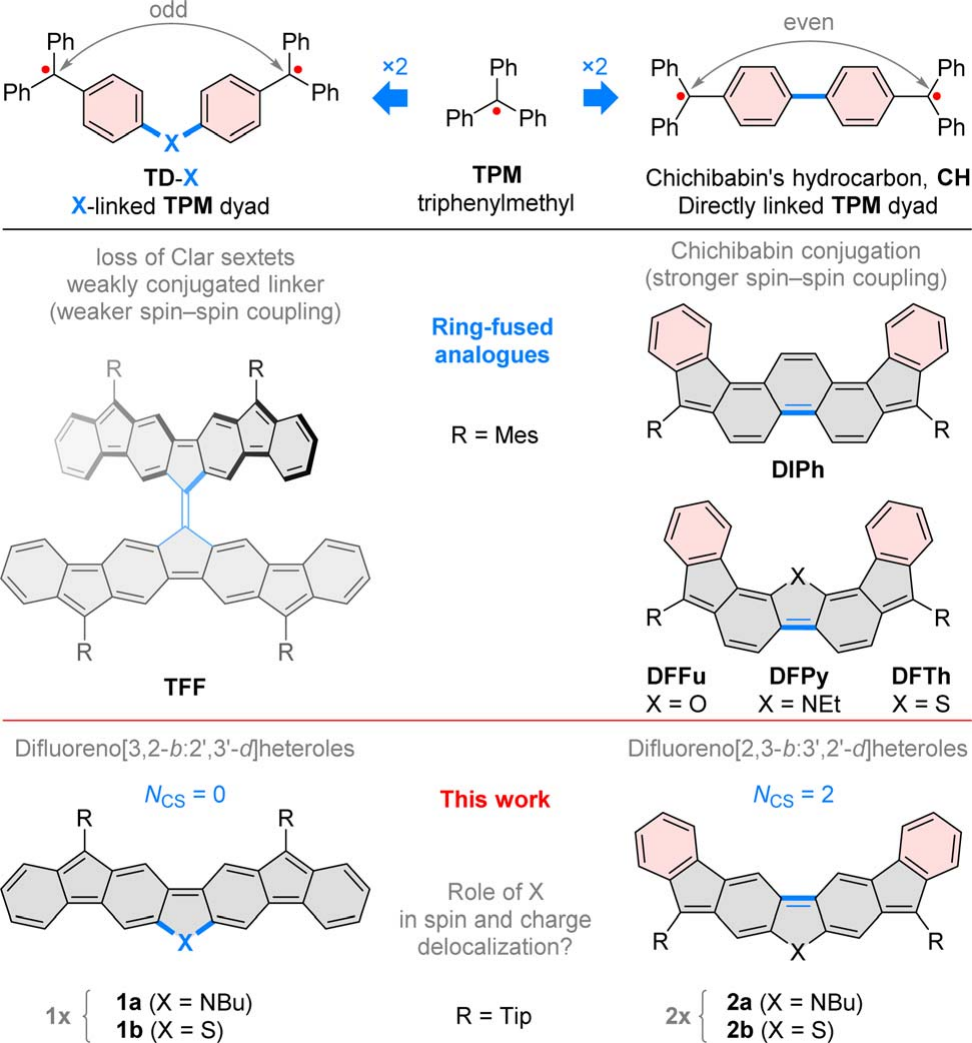
Difluorenoheteroles and the influence of ring fusion on spin–spin interactions in indeno-fused oligoradicals. Mes, mesityl; Tip, 2,4,6-tri(isopropyl)phenyl. NCS, clar-sextet count.

Chichibabin's hydrocarbon,^1^ one of the oldest known diradicaloids (CH, Chart 1), can be viewed as a pair of triphenylmethyl (TPM) radicals conjoined at *para*-phenyl positions. Such a topology leads to a relatively strong interaction between the two spin centers, resulting in a singlet ground state.^2^ However, this interaction alone is insufficient to ensure good ambient stability, unless additional modifications, such as electron-withdrawing groups,^3^ are introduced. In particular, the chemical robustness of Chichibabin's hydrocarbon can be enhanced by ring fusion and peripheral substitution, as illustrated by the recently reported derivatives of CH, such as 5,10-dimesityldiindeno[1,2-*a*:2′,1′-*i*]phenanthrene (DIPh),^4^ its heterocyclic analogues DFFu,^5^DFPy and DFTh,^6^ and some coronoid oligoradicaloid structures.^7–9^ These systems exemplify the use of indeno fusion^10–12^ as a strategy for elaborating stable di- and oligoradicaloids, which are of interest as organic semiconductors,^6,13,14^ redox-active systems,^15^ ion receptors,^8^ and chiral materials.^16,17^ Heterocycle fusion, showcased by DFFu, DFPy and DFTh, is a complementary approach to effectively stabilizing open-shell organics, with diverse recent examples documented in the literature.^18–23^

The spin–spin interaction in such diradicaloids can be engineered by modifying the topology of the π-system. A simple alteration of the original Chichibabin structure involves insertion of a monoatomic linker X, potentially bearing an additional substituent, in between the two constituent TPM units (TD-X, Chart 1). An early attempt to obtain such a diradical with X = O (TD-O), produced a transiently paramagnetic species,^24–27^ which apparently oligomerized on standing. N-linked diradicals TD-NR (R = H, aryl)^27,28^ can be made more stable by chlorination of aryl rings,^28^ however communication between radical centers is weakened by the nonplanarity of the π system.

Rajca's trimethylenemethane benzologue^29^ and Wu's fluorenyl dendrons^30^ offer examples of C-linked oligoradicaloids based on the TD-X conjugation type, displaying respectively ferromagnetic and antiferromagnetic coupling between spin centers. The recently reported tetrafluorenofulvalene (TFF, Chart 1), which can be seen as a dyad of TD-C moieties, features a complex relationship between its electronic state and the center alkene bond strength.^31^TFF differs from the former two systems in that a fully planarized conjugation pathway exists between the two formal spin centers in each of the TD-C subunits.

Our investigations of TFF suggested that the extension of the conjugation pathway introduced by the X linker may produce weakly coupled diradicals with unique spin and redox characteristics. In particular, we considered two isomeric difluorenoheterole motifs 1a–b (labeled jointly 1x) and 2a–b (2x, Chart 1), in which the seemingly similar ring fusion patterns produce very different spin-coupling topologies. While 2x can be analyzed as CH-like systems with even-electron coupling pathways, in 1x, the shortest path between formal spin centers contains an odd number of atoms and includes the heteroatom linker X. In addition, the closed-shell formulae of 1x contain no Clar sextets (*N*_CS_ = 0), whereas two sextets are present in 2x, similarly as in other diindeno analogues of CH. While 1x can be expected to be more weakly coupled that their 2x isomers, the involvement of X bridges in conjugation is far less evident. To study the latter effect, we focused on difluorenopyrroles (1a and 2a, X = NR) and difluorenothiophenes (1b and 2b, X = S). Our choice was motivated by the electron-rich character of both heterocycles, which was thought to facilitate exploration of positively charged states, and the expected difference in π-conjugation between a second- and third-row heteroatom centers.

**Scheme 1:**
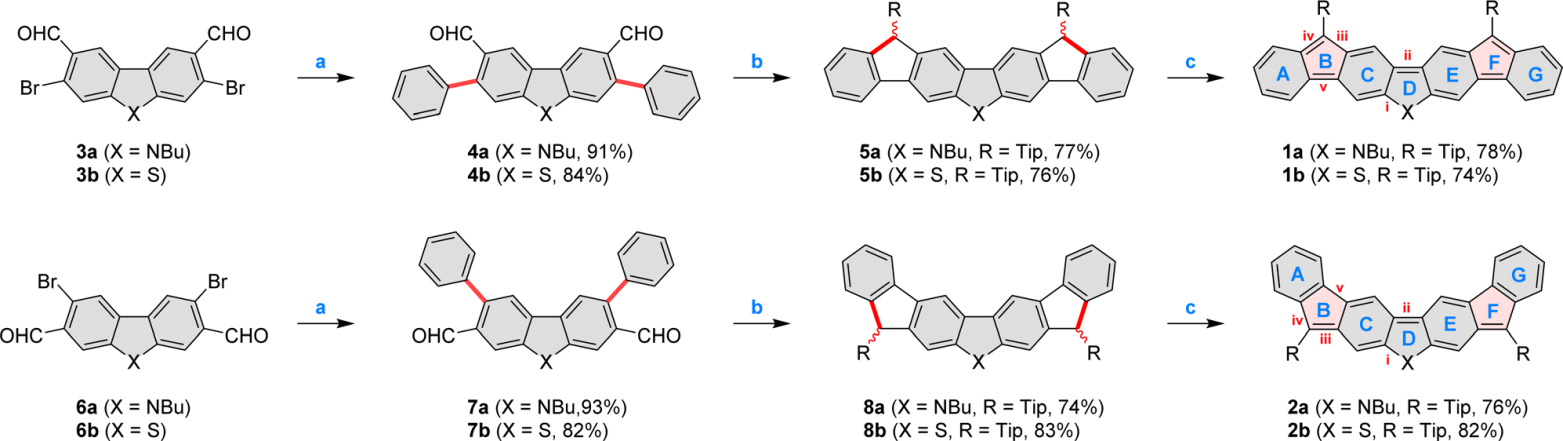
Synthesis of difluorenoheteroles 1x and 2x. Reagents and conditions: (a) phenylboronic acid (2.5 equiv.), Pd(PPh_3_)_4_ (0.1 equiv.), Na_2_CO_3_ (8.0 equiv.), dioxane, H_2_O, 24 h; (b) (1) TipMgBr (0.5 M solution in THF, 6.0 equiv.), THF, overnight. (2) BF_3_·Et_2_O, DCM, 15 min; (c) (1) *t*-BuOK (2 M in 2-MeTHF, 6.0 equiv), THF, (2) CuI (4.0 equiv.), 15 min. For the synthesis of the new dialdehydes 3a, 3b, and 6b, see the ESI.† New bonds and modified rings, are shaded in red. Labelling of key rings (blue) and bonds (red) is given for 1x and 2x.

The four difluorenoheteroles 1a, 1b, 2a, and 2b were obtained using a three-step procedure summarized in Scheme 1. Syntheses of the requisite dibromodialdehydes 3a, 3b, 6a,^32^ and 6b were designed by taking into account intrinsic reactivities of dibenzothiophene and carbazole (see the ESI†). Subsequently, the seven-ring framework of each difluorenoheterole was constructed by double Suzuki coupling followed by Grignard addition of the bulky 2,4,6-tri(isopropyl)phenyl (Tip) group, and electrophilic pentannulation. In each case, formyl groups were strategically placed at the heterocyclic core of 4a–b and 7a–b, to ensure complete regioselectivity of ring closures in step b, which could not be achieved with *ortho*-formylphenyl substituents. Ultimately, the resulting dihydro precursors 5a, 5b, 8a, and 8b, were deprotonated using potassium *tert*-butoxide, and conveniently oxidized with copper(i) iodide. The desired diradicaloids were obtained in high yield and isolated as crystalline solids. 1a and 1b were relatively unstable, and had to be handled and stored in an inert atmosphere. Their isomeric congeners had better stability: refrigerated solid samples 2a and 2b could be stored in air, but the corresponding solutions would gradually decompose over the course of several hours. In spite of structural similarities, the four difluorenoheteroles showed markedly different colors in the solid state, being respectively green (1a), brown (1b), red (2a), and pink (2b).

**Fig. 1:**
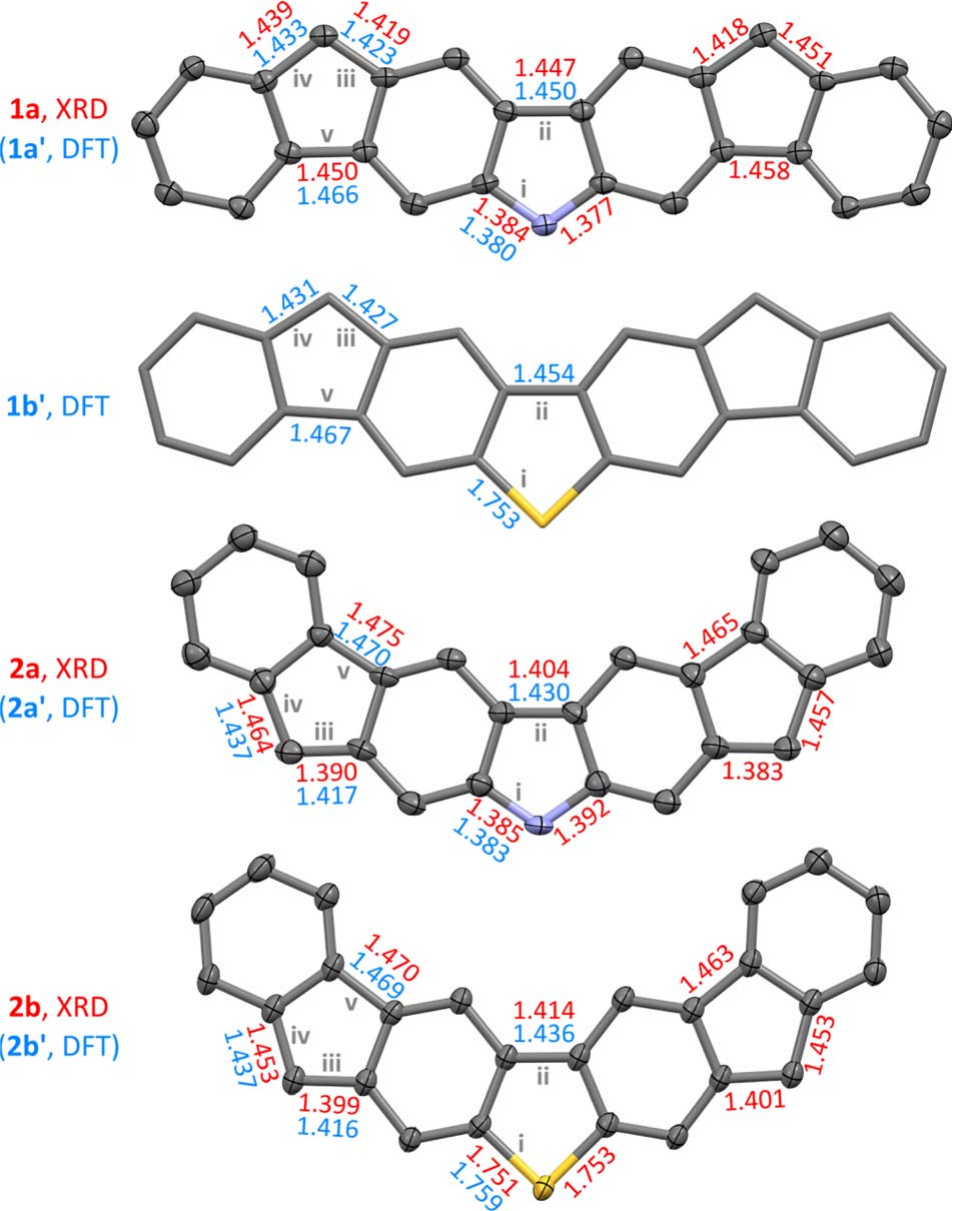
Molecular structures and selected bond distances (Å) of difluorenoheteroles, as obtained from single-crystal X-ray diffraction analyses (XRD, red labels) and DFT calculations (GD3BJ-CAM-B3LYP/6-31G(d,p), broken-symmetry singlets, 1x′ and 2x′ structures, with R = H, blue labels). For 1a, 2a, and 2b, XRD geometries are shown, with solvent molecules, hydrogen atoms, and substituents removed for clarity (thermal ellipsoids at the 50% probability level). C–C bond precision of these XRD geometries is 0.0038 Å (1a), 0.0084 Å (2a), and 0.0029 Å (2b), respectively. A DFT-optimized geometry is shown for 1b′ (R = H). Interatomic distances are given for bonds i–v, as defined in Scheme 1.

Single crystals of the four difluorenoheteroles were grown using vapor diffusion under strictly inert conditions (see the ESI†). X-ray diffraction analyses performed on these crystals confirmed the structures of all diradicaloids, except that, in the case of 1b, the quality of diffraction data was insufficient for complete refinement. As a complementary source of structural data we used DFT calculations, which were performed for the substituent-free systems 1a′, 1b′, 2a′, and 2b′, for all of the relevant spin states and oxidation levels. Experimental and theoretical geometries of each difluorenoheterole are very similar (Fig. 1, and ESI†): each system is predicted to be perfectly planar in the gas phase (*C*_2v_ point symmetry), and the planarity is largely preserved in the solid state. No π stacking interactions were however observed in the crystals, apparently because of the steric hindrance introduced by the bulky Tip groups.

The balance between quinoidal and radicaloid contributions can be assessed using relative lengths of bonds iii contained in the outer five-membered rings (Scheme 1). In 2a and 2b, these distances are significantly longer than formal double bonds but they are nevertheless shorter than the corresponding iv distances (Fig. 1). The difference between iii and iv distances becomes smaller in the 1x series, implying a relatively higher diradicaloid character. Structural variations within the center heterole rings reveal further differences: the center bond ii in 2x is somewhat shorter than the corresponding distance in 1x, in line with the Chichibabin-like quinoidal contribution. Conversely, slight shortening of the C–X bonds (i) in the 1x systems may be thought to indicate involvement of heteroatoms in π-conjugation between the spin centers.

All difluorenoheteroles show distinct room-temperature magnetism, consistent with non-negligible populations of the respective triplet states. The paramagnetic nature of solution samples was inferred from variable-temperature ^1^H NMR spectroscopy (Fig. S46–S49†). Specifically, 2a and 2b yielded sharp spectra at 200 K, but the linewidths would progressively increase when the samples were heated to room temperature. In contrast, 1a produced a broadened spectrum even at 160 K, whereas 1b remained NMR-silent from room temperature down to 180 K. The relative triplet populations cannot however be inferred from these observations since in the fast exchange limit the paramagnetic broadening of NMR lines depend additionally on hyperfine coupling constants and exchange rates,^33^ which may differ among the species being compared. The presence of triplets was verified for compounds 1a, 2a, and 2b, which showed characteristic zero-field splitting patterns in their ESR spectra (Table 1, Fig. S50 and S51†). For 1b, no triplet species was observed, possibly because of unfavorable relaxation effects. However, in SQUID magnetometry, all four systems revealed temperature dependencies consistent with a singlet ground state and a thermally accessible triplet. Interestingly, the Δ*E*_ST_ gap estimated for 1a using the Bleaney–Bowers model (−4.3(1) kcal mol^−1^) has a larger absolute value than the corresponding value obtained for 1b (−0.71(1) kcal mol^−1^). In comparison, gaps of −1.46(1) and −1.42(1) kcal mol^−1^ were determined for 2a and 2b, respectively. The latter two gaps have much smaller absolute values than those of the corresponding isomers, DFPy and DFTh (−5.0 and −4.3 kcal mol^−1^, respectively).^6^ These results show that the energetics of spin alignment in difluorenoheterole diradicaloids depend in a complex way on the topology of the π system and the identity of the heteroatom.

**Table 1:** Experimental and theoretical characteristics of neutral difluorenoheteroles^a^

Species	Δ*E*_ST_^b^ (kcal mol^−1^)	zfs^e^	^1^H NMR	Species	Δ*E*^CAM^_ST_ b (kcal mol^−1^)	*y* ^CAM^_0_	*y* ^CAS^_0_	*n* ^CAM^_U_	*n* ^CAS^_U_
1a	−4.3(1)^c^	*D* = 109 G	300 K: very broad	1a′	1.21	0.846	0.841	1.800	1.683
	−4.16(10)^d^	*E* = 16 G	160 K: broad						
1b	−0.71(1)^c^	f	200–300 K: unobservable	1b′	0.59	0.914	0.918	1.941	1.835
	−0.93(3)^d^								
2a	−1.46(1)^e^	f	300 K: broad	2a′	1.98	0.761	0.694	1.640	1.387
			200 K: sharp						
2b	−1.42(1)^c^	*D* = 83 G	300 K: broad	2b′	2.45	0.750	0.683	1.624	1.366
			200 K: sharp						

a *y*_0_ and *n*_U_ are, the diradicaloid index and number of unpaired electrons respectively; CAM and CAS denote the respective levels of theory (see text).

b Singlet–triplet gap.

c SQUID magnetometry.

d ESR spectroscopy.

e Triplet zero field splitting parameters.

f Splitting not observable.

**Fig. 2:**
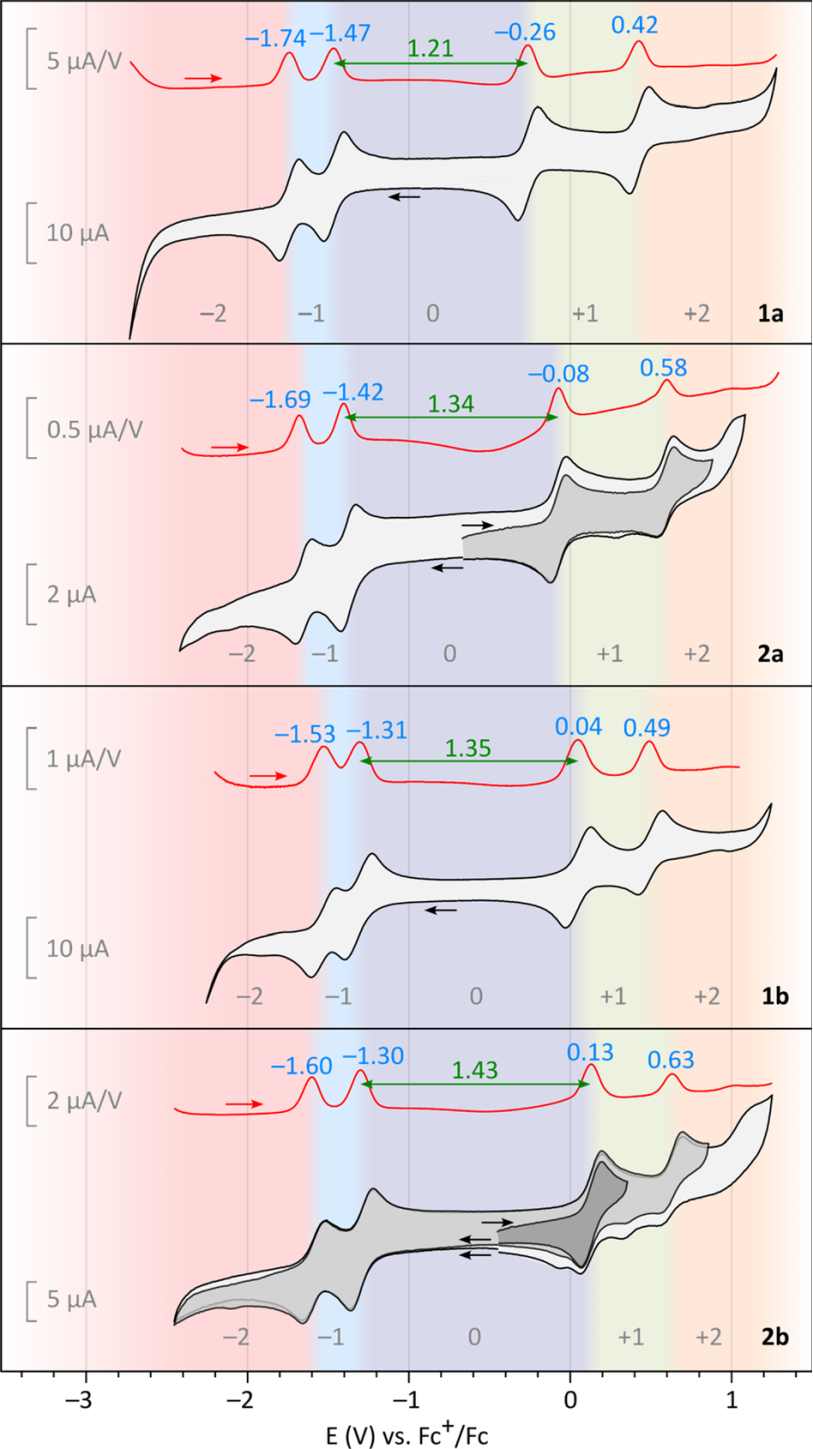
Cyclic voltammetry (CV, black-gray) and differential pulse voltammetry (DPV, red) obtained for difluorenoheteroles 1x and 2x (TBAPF_6_, dichloromethane, 100 mV s^−1^). Blue labels indicate positions of maxima in DPV scans. Electrochemical energy gaps, defined as Δ*E* = *E*_ox1_ − *E*_red1_, are indicated in green. Color shading indicates the oxidation level dominant at a particular potential.

In electrochemical experiments, all difluorenoheteroles, 1a, 1b, 2a, and 2b, exhibited two one-electron oxidations (Fig. 2). In particular, the first oxidation is fully reversible in all systems, and occurs at relatively low potentials. Pyrroles are easier to oxidize than the corresponding thiophenes, indicating a more electron-donating character of the NBu fragment in comparison with the sulfur center. The 1x series is more easily oxidized than the 2x series, with 1a displaying a particularly low *E*_ox1_ potential of −0.26 V *vs.* Fc^+^/Fc. This finding is consistent with better delocalization of the positive charge in the 1x series (*vide infra*). The second oxidation showed very good reversibility in the 1x series, whereas non-reversible processes were observed for 2a and, especially 2b. Two reversible one-electron reduction events were observed for each system, with less variable redox potentials than observed for the oxidations. Overall, the 1x systems show smaller electrochemical energy gaps than their 2x counterparts, with the smallest Δ*E* = 1.21 found for 1a. Interestingly, 2a and 2b are somewhat more electron-rich than their isomers, DFPy and DFTh,^6^ respectively, featuring *E*_ox_ values lower by *ca.* 0.2 eV, and somewhat reduced electrochemical gaps.

**Fig. 3:**
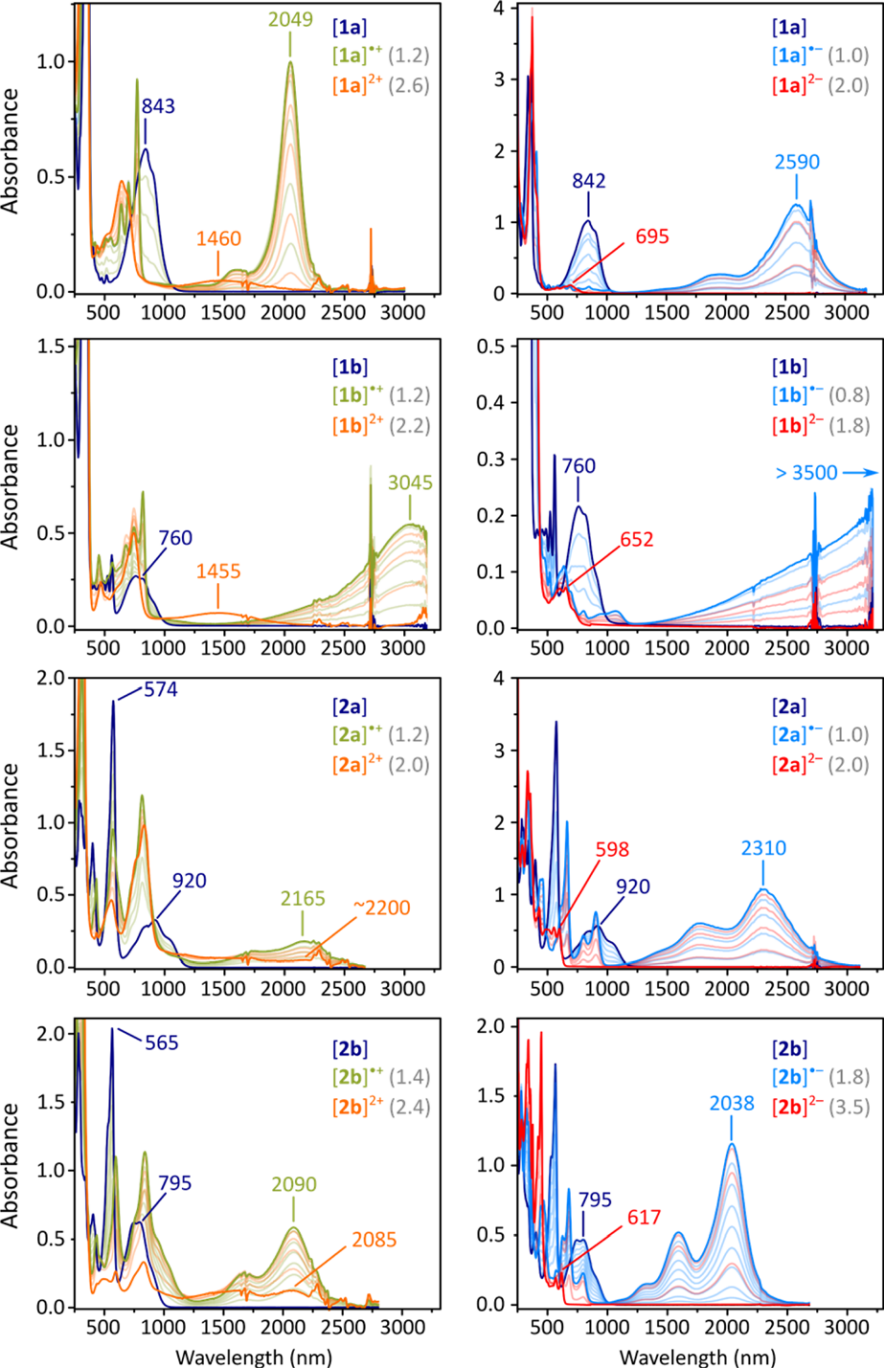
Titration of difluorenoheteroles 1x and 2x with tris(4-bromophenyl)ammoniumyl hexachloroantimonate (BAHA, in dichloromethane, left) and sodium anthracenide (in THF, right). Traces corresponding to maximum concentration of +2, +1, 0, −1, and −2 states are colored in orange, olive green, dark blue, light blue, and red, respectively. Values in gray indicate the number of added equivalents.

The absorption spectra of 2a and 2b each contain an intense maximum at 574 nm and 565 nm, respectively, followed by a group of weaker lower-energy features above 700 nm (navy-blue traces, Fig. 3). These spectra qualitatively resemble those of other CH-conjugated diradicaloids DIPh,^4^DFPy, and DFTh,^6^ but they feature a much better separation between the visible and NIR absorption ranges. In contrast, in the spectra of 1a and 1b, there is no strong feature in the visible region, but the NIR bands have a similar appearance. The apparent optical energy gaps increase in the order 2a < 1a < 2b ∼ 1b, somewhat different than observed electrochemically; however, the gaps are again shown to increase on going from pyrrole-to thiophene-containing diradicaloids.

Electrochemically observed redox events were subsequently reproduced in chemical oxidation and reduction experiments (Fig. 3). Each species could be oxidized in two steps using BAHA, yielding the corresponding radical cation and dication. All of the dications (orange traces, Fig. 3) showed relatively weak NIR absorption bands (1000–2500 nm), whereas striking diversity was observed for the radical cations (olive traces). [2a]˙^+^ and [2b]˙^+^ showed qualitatively similar spectra, with the major NIR maxima at 2165 and 2090 nm, respectively. For [1a]˙^+^, a very narrow and intense band was found at 2049 nm, accompanied by a set of sharp features in the 500–800 nm range. Remarkably, the thiophene containing [2a]˙^+^ featured a spectacular shift of its NIR maximum (3045 nm), in opposition to the energy gap relationship established for the neutral states.

Reduction experiments, carried out using sodium anthracenide (NaA) in THF, revealed similar irregularities in the monoanions of our difluorenoheteroles (blue traces, Fig. 3). Radical anions [2a]˙^−^ and [2b]˙^−^, show strong NIR absorptions, reminiscent of those of the respective cations, with energy gaps correlating with those of the neutral states. The NIR band of [1a]˙^−^ already appears at very low energies (2590 nm, 0.48 eV), but a spectacular red shift was observed for the thiophene counterpart [1b]˙^−^. In this species, the NIR absorption extended beyond the measurement limit of our spectrometer (3300 nm), with an expected position of the maximum of *λ*_max_ > 3500 nm. Attempts to record an FT-IR spectrum of [1b]˙^−^ met with only partial success, because of the low stability of this species and instrumental difficulties (Fig. S56†). However, available data suggest that the low-energy absorption may extend up to *ca.* 2400 cm^−1^ (*ca.* 4200 nm). Using these values, the energy gap of [1b]˙^−^ can be estimated to lie within the 0.30–0.35 eV range.

By using a larger excess of NaA, the difluorenoheteroles could be cleanly reduced to the corresponding dianions [1x]^2−^ and [2x]^2−^ (Fig. 3, red traces). The dianions show much larger energy gaps than the neutral parents or any other ionic states, with somewhat more red-shifted bands found for [1a]^2−^ and [1b]^2−^ (*λ*_max_ = 695 and 652 nm, respectively). These observations agree with the expected closed-shell character of the difluorenoheterole dianions. The energy gap was similarly diminished in the quadruply charged [TFF]^4−^,which may be viewed as a “doubled” analogue of the [1x]^2−^ ions.^31^

Electronic structure features of substituent free difluorenoheteroles were investigated using geometries and densities obtained at the GD3BJ-CAM-B3LYP/6-31G(d,p) level of theory (denoted CAM) along with CAS-SCF(2,2,UNO)/6-31G(d,p) calculations (CAS), carried out on optimized CAM geometries (Table 1). All optimizations yielded flat, *C*_2v_-symmetric structures, each containing a pair of equivalent fluorenyl subunits. For the neutral open-shell singlets, diradicaloid indices *y*_0_ (ref. 34) and numbers of unpaired electrons *n*_U_^35^ calculated using both methods show the same trend, *i.e.*^1^2a′ ≈ ^1^2b′ < ^1^1a′ < ^1^1b′. Thus, the influence of the heteroatom on the open-shell character is notably weak in the 2x′ singlets, in line with the predominant Chichibabin-like conjugation along the biphenyl backbone of these molecules. In contrast, the 1x′ singlets reveal a higher open-shell character than their 2x′ counterparts. Most strikingly, there is a significant difference of *n*_U_ values between 1b′ and 1a′ (*ca.* 0.15 at the CAS level), which may be seen as an indication that the NR fragment in 1a′ provides a more efficient coupling pathway between radicaloid centers than the S atom in 1b′.

CAM geometry optimizations carried out for oxidized and reduced forms of 1x′ and 2x′ revealed unexpected symmetry lowering of some states (Fig. S57 and S58†). In most cases, the *C*_2v_ symmetry of the neutral singlets and triplets was retained in the ions, and structural changes were limited to variations of bond lengths. However, for three radical ions, [1a′]˙^−^, [1a′]˙^+^, and [1b′]˙^−^, *C*_s_ symmetric geometries were obtained, with different bonding distances in the two conjoined fluorenyl subunits. Such geometries are consistent with a distonic nature of these radical ions, *i.e.* with preferential localization of the spin and charge on different fluorenyl fragments. The automerization (degenerate rearrangement) of such species would require only a relatively small bond shift, and is expected to have a very low energy barrier. *C*_2v_-symmetric stationary points located for [1a′]˙^−^ and [1a′]˙^+^ are transition states according to frequency analyses, but their relative energies are in the 0.1–0.3 kcal mol^−1^ range. The symmetry of global energy minima in the radical ions of 1x may depend on *e.g.* substitution or solvation and give very soft potentials, and may be represented by the CAM calculation in only an approximate way.^36^ For consistency, further discussion corresponds to global minima identified at the CAM level.

**Fig. 4:**
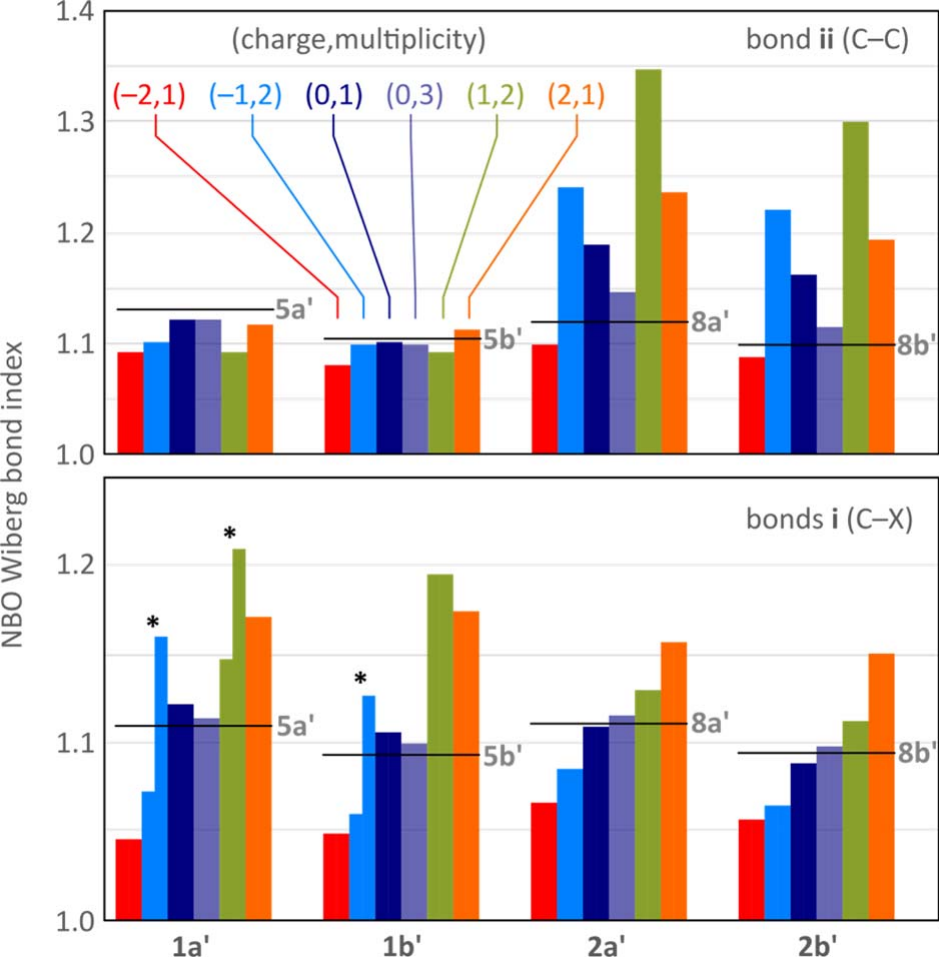
Evolution of selected Wiberg bond indices as a function of increasing charge calculated for the unsubstituted structures 1a′, 1b′, 2a′, and 2b′ (NBO3/CAM-B3LYP-GD3BJ/6-31G(d,p)). Black horizontal lines indicate reference values obtained for the dihydro reference systems 5a′, 5b′, 8a′, and 8b′, respectively (Scheme 1, R = H). Bonds i and ii (defined in Scheme 1) are shown using separate color scales. For the *C*_s_-symmetric structures (denoted with asterisks), *i.e.* [1a′]˙^−^, [1b′]˙^−^, and [1a′]˙^+^, the two i bonds have unequal Wiberg indices and are shown with two separate bars.

In all difluorenoheteroles, coupling between the two halves of the molecule can involve both the C–C pathway (bond ii) or the C–X–C pathway (bonds i), but the interaction topology differs between the 1x and 2x series. Relative contributions of these two pathways as a function of the increasing charge of the system can be inferred from the variations of Wiberg indices^37^ obtained for bonds i and ii in a natural bond orbital analysis^38^ (NBO, Fig. 4). The indices of 1a′, 1b′, 2a′, and 2b′ are conveniently compared with values obtained for the corresponding dihydro reference systems, 5a′, 5b′, 8a′, and 8b′, respectively. All reference systems show relatively weak conjugation between the two fluorene sections of the molecules: the i (C–X) and ii (C–C) bond indices take relatively low values of 1.09–1.11 and 1.10–1.13, respectively. These indices are essentially independent of the ring fusion pattern (5x′*vs.*8x′), in line with the absence of cross-conjugation in the dihydro references.

Neutral singlets in the 1x′ series have somewhat stronger i bonds than the dihydro ref. 5x′. This is in contrast with the 2x′ systems, in which the i bond indices are lower than in the respective 8x′ references, and may indicate TD-X-like conjugation in 1x′ (as defined in Chart 1). In comparison with the singlets, the i and ii Wiberg indices in the triplet states ^3^[1x′] and ^3^[2x′] are generally closer to the values observed for dihydro references, implying a weaker interaction between the fluorenyl subunits in the high-spin state. Importantly, in the majority of oxidation levels of 2x′, the ii index is higher than in 8x′, in line with a Chichibabin-like coupling. Interestingly, the interaction is the strongest in the radical ions [2x′]^+^ and [2x′]^−^, apparently reflecting the involvement of quinoidal conjugation in the delocalization of either the positive or negative charge. The i index decreases in the anions of 2x′, but it becomes higher in the positively charged states, notably in the dications [2x]^2+^, a feature attributable to onium-type resonance (*vide infra*). Conversely, the ii index is consistently low at all oxidation levels of 1x′, whereas the i index shows a pronounced variation, becoming particularly high for cationic states. Its values are differentiated in the *C*_s_ symmetric states [1a′]˙^−^, [1a′]˙^+^, and [1b′]˙^−^, showing that indeed one of the C–X bonds is stronger than the other.

**Fig. 5:**
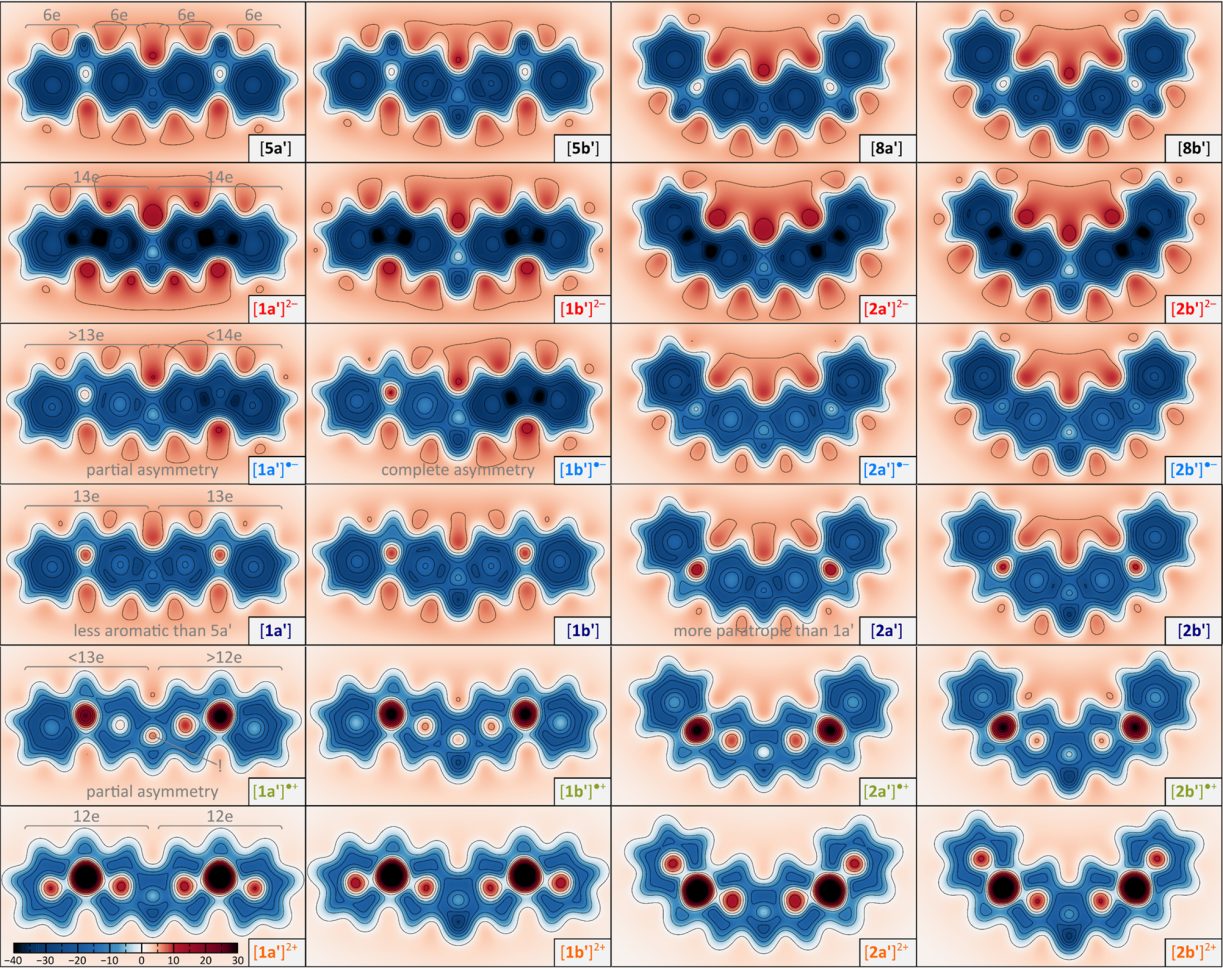
NICS(1) XY_*zz*_ scans for the different oxidation levels 1x′ and 2x′ and the dihydro ref. 5x′ and 8x′ (X = S, NH, R = H). The maps show negative values of the *zz* component of the magnetic shielding tensor calculated 1 Å above the mean plane of each molecule at the GIAO/CAM-B3LYP-GD3BJ/6-31G(d,p) level of theory. Electron counts shown for 1a′ and 5a′ show the dominant.

NICS maps calculated for the difluorenoheteroles (1x′, 2x′) and their dihydro analogues (5x′, 8x′) reveal the relationship between local aromaticity features and the oxidation state (Fig. 5). 5x′ and 8x′ offer a convenient reference picture, displaying the expected aromaticity in all benzenoid rings and in the central heterole unit. Their fully conjugated counterparts 1x′ and 2x′ progress from strong diatropicity to strong paratropicity as their charge is raised from −2 to +2, however, this general trend is superimposed with differences specific to each of the two series. The NICS maps in the neutral singlet states of 1x′ and 2x′ show diminished benzenoid aromaticity relative to the dihydro references, and to a first approximation may be viewed as unions of two 13-electron fluorenyl radicals. However, the greater paratropicity in the 2x′ series, reveals the effect of inter-subunit interactions, and is in line with their stronger quinoidal character of the Chichibabin-like systems. The dianions [1x′]^2−^ and [2x′]^2−^ are magnetically most uniform, and all of them can be viewed as a fused pair of strongly diatropic 14-electron fluorenyl anions. Likewise, the conjugation in [1x′]^2+^ and [2x′]^2+^ can be interpreted in terms of antiaromaticity of the constituent 12-electron fluorenyl cations. Radical anions [2x′]˙^−^ reveal shieldings that are intermediate between that of the corresponding dianions and the neutral states. In contrast, in the [1x′]˙^−^ structures, one flurenyl unit shows radical-like magnetism, whereas the other unit is strongly diatropic, implying its predominantly anionic nature. Thus, the NICS maps provide compelling evidence for the distonic character of the [1x′]˙^−^ anions. A similar analysis is also valid for the radical cations [1x′]˙^+^ and [2x′]˙^+^, with the noteworthy exception of the center heterole ring, which is more strongly paratropic than in the corresponding neutral and dicationic states.

**Fig. 6:**
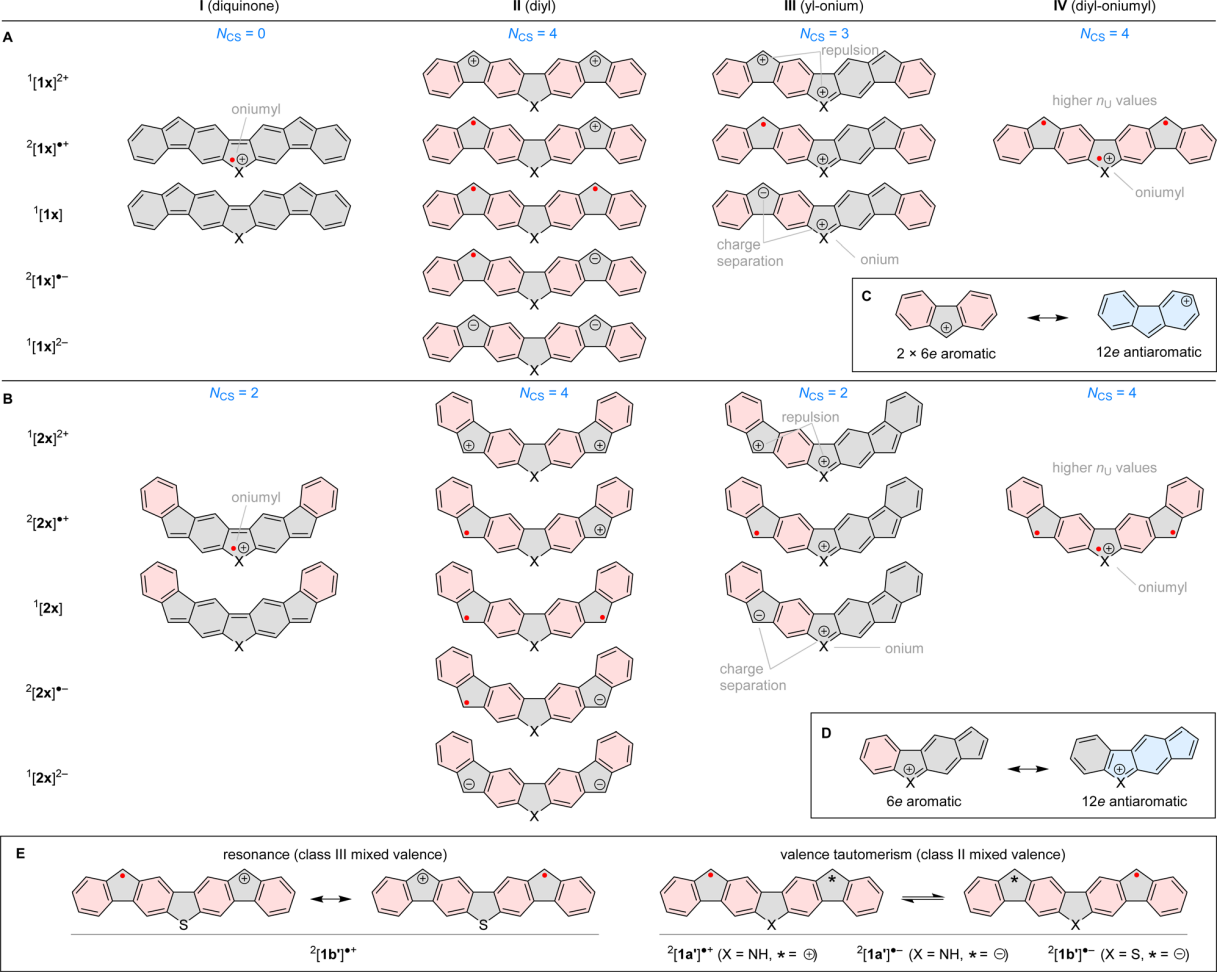
Conjugation in difluorenoheteroles and their ions. R groups are omitted for clarity. Clar sextets and Hückel-antiaromatic circuits are shaded in red and blue, respectively. (A) Conjugation in 1x (X = S, NR), (B) conjugation in 2x (X = S, NR), (C) antiaromaticity the of fluorenyl cation; (D) indacene-like antiaromaticity of benzo[*b*]indeno[5,6-*d*]heterole cations; (E) mixed-valence characters of radical ions, according to CAM calculations.

The combined experimental and theoretical evidence obtained for difluorenoheteroles can be rationalized in terms of several types of delocalization contributions I–IV, summarized in Fig. 6A and B. Diquinone contributors I can be drawn for both neutral 1x and 2x, but they feature low Clar sextet counts *N*_CS_ (0 and 2, respectively), which makes them less relevant. Analogous structures can formally also be proposed for radical cations by assuming non-octet oniumyl heteroatom sites, but their contribution will likewise be limited. Clar sextet counts are maximized in “diyl” structures II (*N*_CS_ = 4), which can be constructed for all oxidation levels of both 1x and 2x, and are expected to be the dominant resonance contributors. The diyl structures can also be reinterpreted as consisting of two fluorene units, each at a specific oxidation level (anion, radical, or cation). Ring currents observed in the NICS maps (Fig. 5) are largely consistent with such a formulation, *e.g.* the intrinsic antiaromaticity of the fluorenyl cation (Fig. 6C), is reflected in the magnetic features of the [1x′]^2+^ and [2x′]^2+^ cations.

The presence of heteroatoms in 1x and 2x provides additional opportunities for resonance: in the “yl-onium” structures III, which are available for all nonnegatively charged states, a formal positive charge is placed on the X site whereas a cationic, radical, or anionic site is located in one of the outer 5-membered rings. Type-III contributions are particularly important in radical cations, wherein they do not lead to either charge separation or charge repulsion. Yl-onium structures can be used to construct indacene-like antiaromatic circuits (Fig. 6D), which provide an appealing rationale for the increased paratropicity of heterole rings observed in [1x′]˙^+^ and [2x′]˙^+^. Finally, “diyl-oniumyl” contributions IV can be proposed to explain the elevated *n*^CAM^_U_ values (*ca.* 1.2) determined for [1x′]˙^+^ and [2x′]˙^+^.

For radical cations and anions of 1x′ and 2x′, canonical structures II and III are inherently unsymmetrical, because of the distonic placement of formal charge and spin sites. This feature may lead to two distinct Robin–Day classes of organic mixed valence:^39,40^ class III, with spin and charge uniformly delocalized over the whole ion (*C*_2v_ symmetry); and class II, characterized by lower symmetry (*C*_s_) and valence tautomerization. CAM geometries and densities (see above) indicate that all 2x ions should be class-III CT systems, in line with the vibronic structure of their lowest-energy absorption bands. In the 1x series, the [1b′]˙^+^ state is also predicted to belong to class III, and its structure can therefore be described in terms of resonance between the two equivalent type II structures (Fig. 6E). In the remaining ions, [1a′]˙^+^, [1a′]˙^−^, and [1b′]˙^−^, the two type-II structures would correspond to valence tautomers.

## Conclusions

The two families of difluorenoheteroles described in this work provide a rare opportunity for side-by-side comparison of isomeric structures containing distinct conjugation patterns, thus offering a valuable insight into heteroatom-mediated conjugation effects in organic diradicaloids and their ions. The new fusion pattern introduced herein, based on the X-linked triphenylmethyl dyad (TD-X) motif, produces diradicaloids with smaller energy gaps and better redox reversibility than their Chichibabin-like (CH) counterparts, making the former systems of interest in organic semiconductor applications. The TD-X difluorenoheteroles are less quinoidal than the corresponding CH systems, showing instead a stronger involvement of the heteroatom in the overall conjugation. This effect leads to different properties of the TD-S and TD-NR analogues: quite remarkably, the TD-NR diradicaloid features a much larger singlet–triplet gap, showing the ability of the nitrogen bridge to promote antiferromagnetic alignment of spins.

The TD-X difluorenoheteroles are particularly notable for the extremely NIR-shifted spectra of their radical cations and anions, a characteristic that can be linked to their mixed-valence character. Unlike their CH analogues, which display complete delocalization of spin and charge, leading to fully symmetrical structures, most of the TD-X radical ions are predicted to have low-symmetry structures consistent with distonic placement of spin and charge, and corresponding to class-II mixed valence. Efforts to explore this elusive property and correlate it with electronic characteristics of these ions are ongoing in our laboratory.

## Data availability

The crystallographic data was provided in Cambridge Structural Database, and the other necessary data of this study have been provided in the ESI.†

## Author contributions

B. P. and M. S. designed the experiments and co-wrote the manuscript. B. P., T. K. and M. A. M. performed all the synthetic exeperiments and characterization. T. L. performed X-ray crystallographic analyses. P. J. C. performed electrochemical analyses. C. J. G.-G. performed and analyzed magnetometric measurements. M. S. performed theoretical calculations.

## Conflicts of interest

There are no conflicts to declare.

## Supplementary Material

SC-015-D4SC02459A-s001

SC-015-D4SC02459A-s002

SC-015-D4SC02459A-s003
