# Supplementary material for: Difluorenoheteroles: topological control of π conjugation in diradicaloids and mixed-valence radical ions
Source: Chem Sci. 2024 May 23;15(26):10101–9. doi: 10.1039/d4sc02459a (PMC11220597; doi:10.1039/d4sc02459a)
Supplement: SC-015-D4SC02459A-s002 [file SC-015-D4SC02459A-s002.pdf]

Electronic Supplementary Material (ESI) for Chemical Science.
This journal is © The Royal Society of Chemistry 2024

Difluorenoheteroles: Topological Control of π Conjugation in Diradicaloids and Mixed-Valence Radical Ions

Supporting Information

by Bibek Prajapati, Tendai Kwenda, Tadeusz Lis, Piotr J. Chmielewski, Carlos J. Gómez-García, Marcin A. Majewski, and Marcin Stępień

Table of Contents

1. General 3
1.1. Synthetic Methods 3
1.2. Analytical Methods 3
1.3. Computational Methods 4
2. Synthesis and Characterization 5
2,7-Dibromo-9-butyl-9*H*-carbazole-3,6-dicarbaldehyde (**3a**) 5
9-Butyl-2,7-diphenyl-9*H*-carbazole-3,6-dicarbaldehyde (**4a**) 6
6-Butyl-12,15-bis(2,4,6-triisopropylphenyl)-12,15-dihydro-6*H*-diindeno[1,2-*b:2',1'-h*]carbazole (**5a**) 9
6-Butyl-12,15-bis(2,4,6-triisopropylphenyl)-6*H*-diindeno[1,2-*b*:2',1'-*h*]carbazole (**1a**) 11
3,7-Dibromodibenzo[*b,d*]thiophene-2,8-dicarbaldehyde (**3b**) 12
3,7-Diphenyldibenzo[*b,d*]thiophene-2,8-dicarbaldehyde (**4b**) 14
12,15-Bis(2,4,6-triisopropylphenyl)-12,15-dihydrodifluoreno[3,2-*b*:2',3'-*d*]thiophene (**5b**) 16
12,15-Bis(2,4,6-triisopropylphenyl) difluoreno[3,2-*b*:2',3'-*d*]thiophene (**1b**) 18
9-Butyl-3,6-diphenyl-9*H*-carbazole-2,7-dicarbaldehyde (**7a**) 19
7-Butyl-5,9-bis(2,4,6-triisopropylphenyl)-7,9-dihydro-5*H*-diindeno[2,1-*b*:1',2'-*h*]carbazole (**8a**) 21
7-Butyl-5,9-bis(2,4,6-triisopropylphenyl)-7*H*-diindeno[2,1-*b*:1',2'-*h*]carbazole (**2a**) 23
2,8-Dibromo-3,7-bis(dibromomethyl)dibenzo[*b,d*]thiophene (**S1**) 25
2,8-Dibromodibenzo[*b,d*]thiophene-3,7-dicarbaldehyde (**6b**) 27
2,8-Diphenyldibenzo[*b,d*]thiophene-3,7-dicarbaldehyde (**7b**) 28
5,9-Bis(2,4,6-triisopropylphenyl)-5,9-dihydrodifluoreno[2,3-*b*:3',2'-*d*]thiophene (**8b**) 31
5,9-Bis(2,4,6-triisopropylphenyl)difluoreno[2,3-*b*:3',2'-*d*]thiophene (**2b**) 33
3. Aditional Schemes 35
4. Experimental Data 36
4.1. X-ray Crystallography 36
4.2. NMR Spectroscopy 42

4.3. ESR Spectroscopy 46
4.4. Magnetometry 47
4.5. IR Spectroscopy 49
5. Computational Data 50
5.1. Energies and Geometries 50
6. References 53

1. General

1.1. Synthetic Methods

Tetrahydrofuran and 1,4-dioxane were dried using a commercial solvent purification system. Dichloromethane was distilled from calcium hydride when used as a reaction solvent. All other solvents and reagents were used as received. Compounds **S1**[1], **S2**[2], **6a**[3], **S3**[4] were prepared according to the modified literature procedures.

2,4,6-Triisopropylphenylmagnesium bromide (TIPMgBr, 0.5 M in THF): Magnesium turnings (437 mg, 18.0 mmol) were suspended into a flask and dry tetrahydrofuran (30 mL) was added under N_2 atmosphere. A drop of dibromoethane was added and the mixture was stirred at room temperature for 10 minutes. 1-Bromo-2,4,6-triisopropylbenzene (4.25 g, 15 mmol) was then added in one portion. The reaction mixture was stirred at 60 °C for 1 h. After cooling to room temperature and settling, the solution was collected from above the remaining solids and stored in a Schlenk tube under N_2 for further use.

1.2. Analytical Methods

^{1}H NMR spectra were recorded on high-field spectrometers (^{1}H frequency 500.13 or 600.13 MHz), equipped with broadband inverse or conventional gradient probe heads. Spectra were referenced to the residual solvent signals (chloroform-*d*, 7.24 ppm, dichloromethane-*d*$_2$, 5.32 ppm). ^{13}C NMR spectra were recorded with ^{1}H broadband decoupling and referenced to solvent signals ($^{13}CDCl_3$, 77.0 ppm, $^{13}CD_2Cl_2$, 55.0 ppm). High resolution mass spectra were recorded using ESI ionization in the positive mode on Bruker Apex ultra-FT-ICR and MALDI mass spectra were recorded on a JMSS3000 SpiralTOF™-plus 2.0 MALDI-TOF spectrometer. UV-Vis-NIR Absorption spectrometry was performed using Perkin Elmer LAMBDA 1050 UV-NIR spectrometer. Electrochemical measurements (DCM, 0.1 M $[NBu_4][PF_6]$, 293 K) were performed on an Metrohm Autolab potentiostat/galvanostat using a glassy carbon working electrode, platinum wire as the auxiliary electrode, and silver wire as a reference electrode. The voltammograms were referenced against the half-wave potential of Fc^+ /Fc.

Variable temperature susceptibility measurements were carried out with a Quantum Design MPMS-XL-7 SQUID magnetometer, in the temperature range 2 -370 K, with an applied magnetic field of 0.5 T, on polycrystalline samples of compounds, sealed in glass capillaries under inert atmosphere. The samples were measured in heating and cooling scans at a scan rate of 2 K/min. The data (and the fits) were very similar in the cooling and heating scans. The susceptibility data were corrected for the empty glass capillary previously measured using the same conditions and for the diamagnetic contribution of the samples as deduced by using Pascal´s constant tables.[5]

Diffraction data were collected on a Rigaku Oxford Diffraction XtaLAB Synergy-R DW diffractometer equipped with a HyPix ARC 150° Hybrid Photon Counting (HPC) detector using CuKα (λ = 1.5418 Å) at 100 K. Data collection, cell refinement, data reduction and analysis were carried out with the Xcalibur PX software, CRYSALIS CCD and CRYSALIS RED, respectively (Oxford Diffraction Ltd., Abignon, England, 2009). An analytical absorption correction was applied with the use of CRYSALIS RED. All structures were solved by direct methods with the SHELXS-97 program and refined using SHELXL-97 with anisotropic thermal parameters for non-H atoms. In the final refinement cycles, all H atoms were treated as riding atoms in geometrically optimized positions. Crystals of compounds **2b**, **2a**, and **1a** were grown by vapor diffusion of methanol into a solution of dichloromethane. CCDC numbers of compounds **2b (2302632)**, **2a (2302633)**, and **1a (2302634)** contain the supplementary crystallographic data for this paper. These data are provided free of charge by The Cambridge Crystallographic Data Centre via www.ccdc.cam.ac.uk/data_request/cif

ATR-IR spectra in the 4000-1000 cm-1 range were measured on Thermo Scientific Nicolet iS50 spectrometer with Golden Gate (PIKE) attachment. The spectra were collected with resolution of 4 cm-1 and 64 scans were

accumulated. During measurements we applied a KBr beamsplitter and DTGS detector. The spectra were then processed using the OMNIC software package (baseline correction, conversion to nanometer scale).

1.3. Computational Methods

Density functional theory (DFT) calculations were performed using Gaussian 16.[6] DFT geometry optimizations were carried out in unconstrained C_1 symmetry, using molecular mechanics or semiempirical models as starting geometries. The calculations were performed using the hybrid functional B3LYP,[7–9] including the CAM[10] and GD3BJ[11] corrections, and the 6-31G(d,p) basis set. Unrestricted wavefunctions were used for all open-shell systems and broken-symmetry solutions were obtained for all open-shell singlets. Each structure was optimized to meet standard convergence criteria, and the existence of a local minimum was verified by a normal mode frequency calculation. NICS maps (Figure 5) were obtained at the CAM level of theory, by evaluating GIAO shieldings over a square grid of 201 × 201 points and located 1 Å above the plane of the molecule. The plots correspond to the anisotropic shielding value in the direction perpendicular to the cross-section plane. HOMA values were calculated as previously reported, using experiment-based parameters.[16,17] Oligoradicaloid indices were calculated as $y_i = 1 - (n_{\mathrm{HONO}-i} - n_{\mathrm{LUNO}-i})/2$.[18]

2. Synthesis and Characterization

2,7-Dibromo-9-butyl-9*H*-carbazole-3,6-dicarbaldehyde (**3a**)

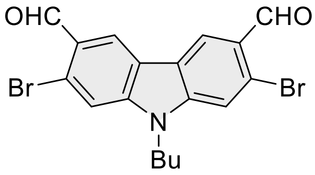

Compound **S3** (6 g, 9.48 mmol) was dissolved in 150 mL of dry tetrahydrofuran and purged with nitrogen for 15 minutes. The solution under nitrogen was cooled to -20 °C and a solution of isopropyl magnesium chloride (9.95 mL, 19.9 mmol, 2 M solution in tetrahydrofuran) was added dropwise keeping the temperature below -20 °C. The mixture was stirred for an hour under that temperature and an anhydrous *N,N* -dimethylformamide (7.35 mL, 94.8 mmol) was added dropwise. The reaction mixture was warmed to an ambient temperature and stirred for an additional 3 hours. The reaction was then quenched aqueous ammonium chloride solution and extracted with chloroform. The organic layer was washed with brine, dried over anhydrous sodium sulfate(VI) and the solvent was removed on a rotary evaporator. The crude mixture was purified via silica column chromatography using 60% dichloromethane in *n*-hexane as an eluent to give compound **3a** (2.87 g, 69%) as a colorless solid.

^{1}H NMR (500 MHz, chloroform-*d*, 300 K): δ 10.42 (2H, s), 8.63 (2H, s), 7.59 (2H, s), 4.24 (2H,t, ^3J = 7.3 Hz), 1.87 (2H, m), 1.43 (2H, m), 0.99 (3H, t, ^3J = 7.4 Hz).

^{13}C NMR (125 MHz, chloroform-*d*, 300 K): δ 191.10, 145.02, 126.58, 125.32, 123.07, 122.53, 114.00, 43.84, 30.81, 20.46, 13.74.

HRMS (APCI–TOF): *m/z*: $[M + H]^+$ Calcd for $C_{18}H_{15}Br_2NO_2$: 437.9523; Found 437.9545.

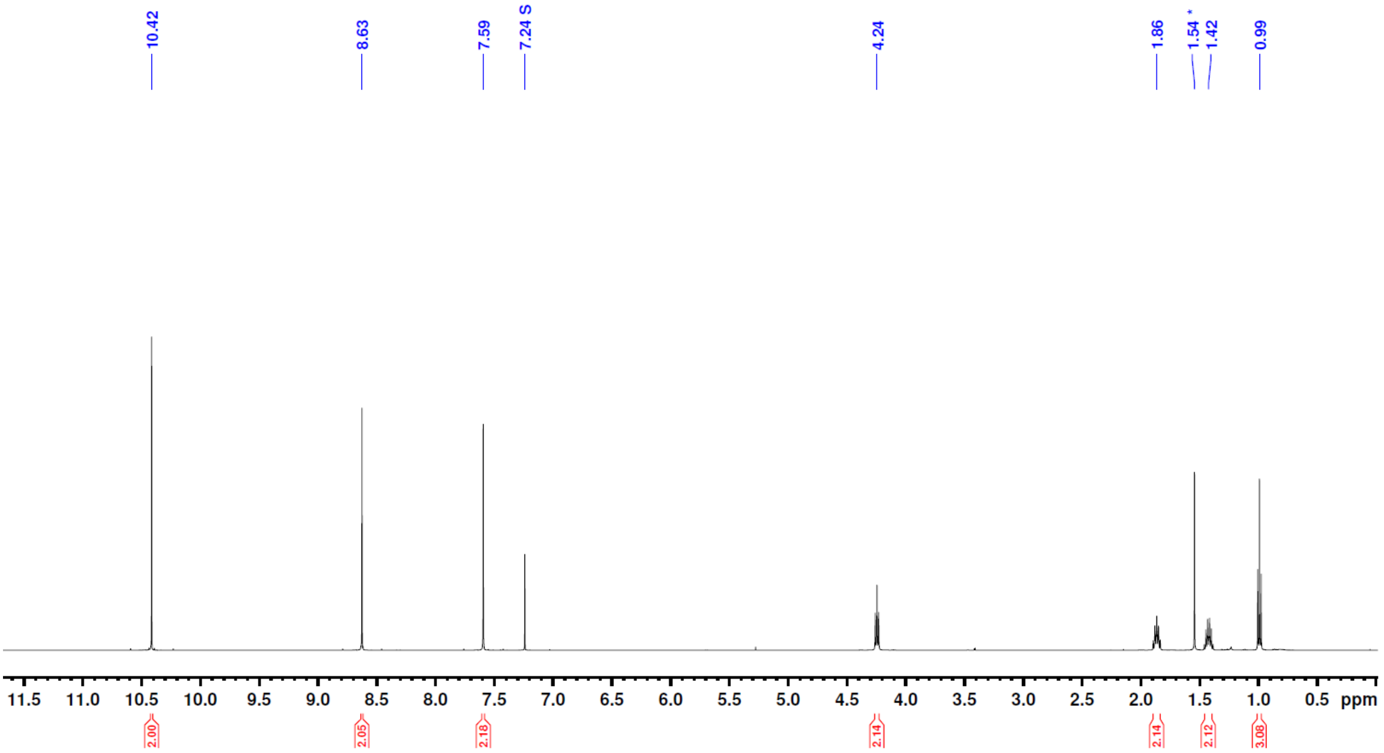

Figure S1. ^{1}H NMR spectrum of compound **3a** (500 MHz, chloroform-*d*, 300 K).

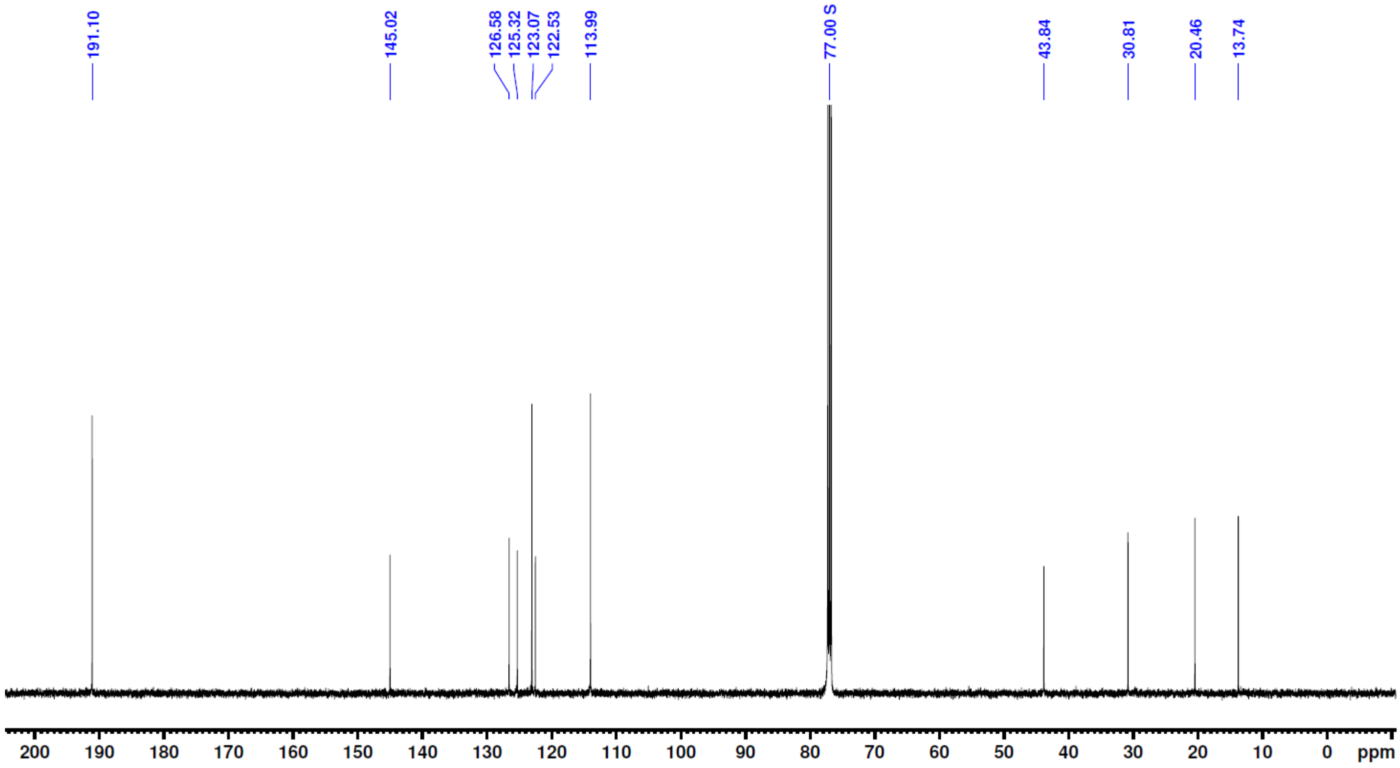

Figure S2. ^{13}C NMR spectrum of compound **3a** (125 MHz, chloroform-*d*, 300 K).

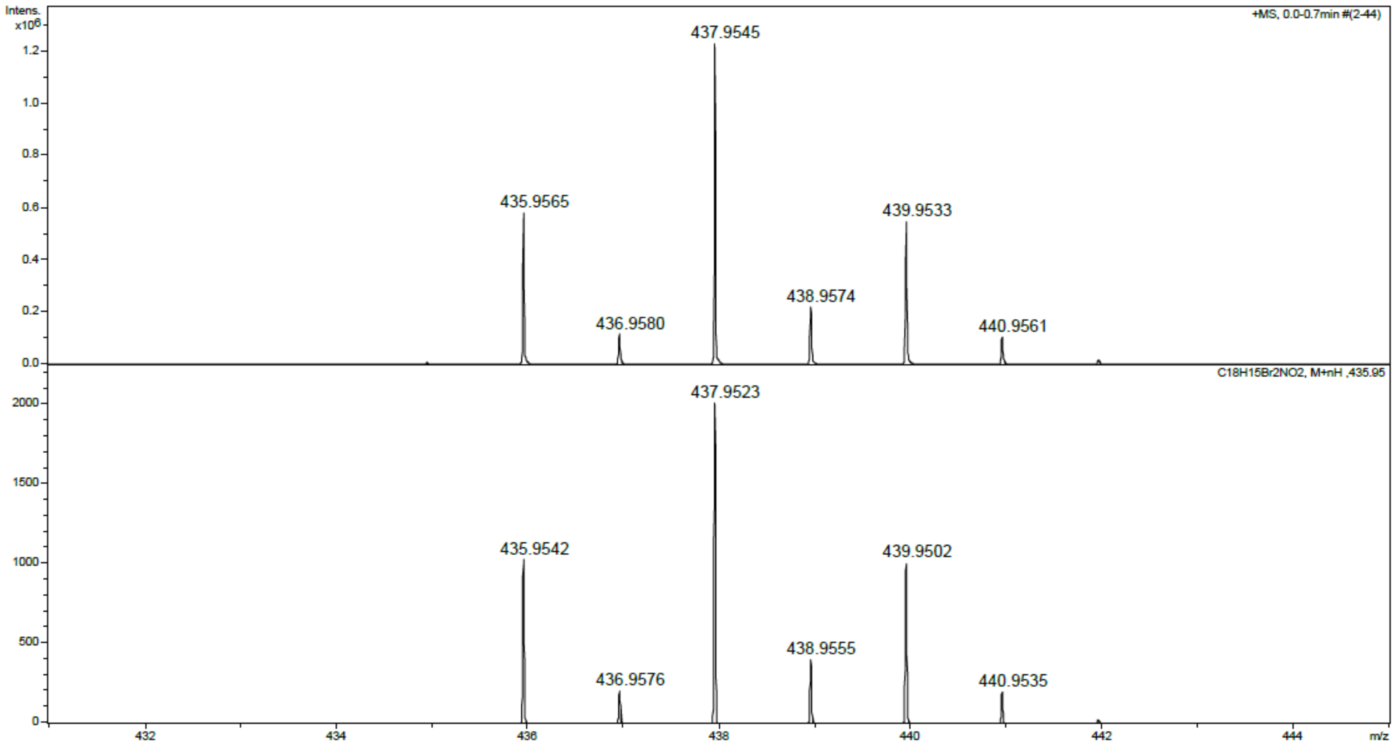

Figure S3. High resolution mass spectrum of compound **3a** (APCI–TOF, top: experimental, bottom: simulated).

9-Butyl-2,7-diphenyl-9*H*-carbazole-3,6-dicarbaldehyde (**4a**)

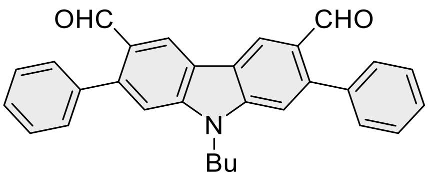

Compound **3a** (400 mg, 0.92 mmol) and phenyl boronic acid (293.6 mg, 2.29 mmol) was dissolved in 100 mL of dioxane and the solution was purged with nitrogen for 15 minutes. The degassed solution of sodium carbonate

(776 g, 7.32 mmol) in 10 mL of water was added to the mixture followed by the addition of tetrakis(triphenylphosphine)palladium(0) (106 mg, 0.09 mmol) The mixture was purged with nitrogen for few minutes and heated at 100 °C for 24 hours. After cooling to room temperature, water was added and extracted with dichloromethane. The organic layer was washed with brine, dried over anhydrous sodium sulfate(VI) and the solvent was removed on rotary evaporator. The crude mixture was purified via silica column chromatography using 70% dichloromethane in *n*-hexane as an eluent to give compound **4a** (359 mg, 91%) as a colorless solid.

^{1}H NMR (500 MHz, chloroform-*d*, 300 K): δ 10.02 (2H, s), 8.88 (2H, s), 7.49 (10H, m), 7.37 (2H, s), 4.34 (2H, t, ^3J = 7.3 Hz), 1.88 (2H, m), 1.41 (2H, m), 0.95 (3H, t, ^3J = 7.4 Hz).

^{13}C NMR (125 MHz, chloroform-*d*, 300 K): δ 191.60, 145.27, 144.46, 138.76, 130.37, 128.40, 128.09, 127.40, 122.76, 121.58, 110.55, 43.56, 30.93, 20.48, 13.79.

HRMS (ESI–TOF): *m*/*z*: [M + K]$^+$ Calcd for $C_{30}H_{25}NO_2$: 470.1517; Found 470.1510.

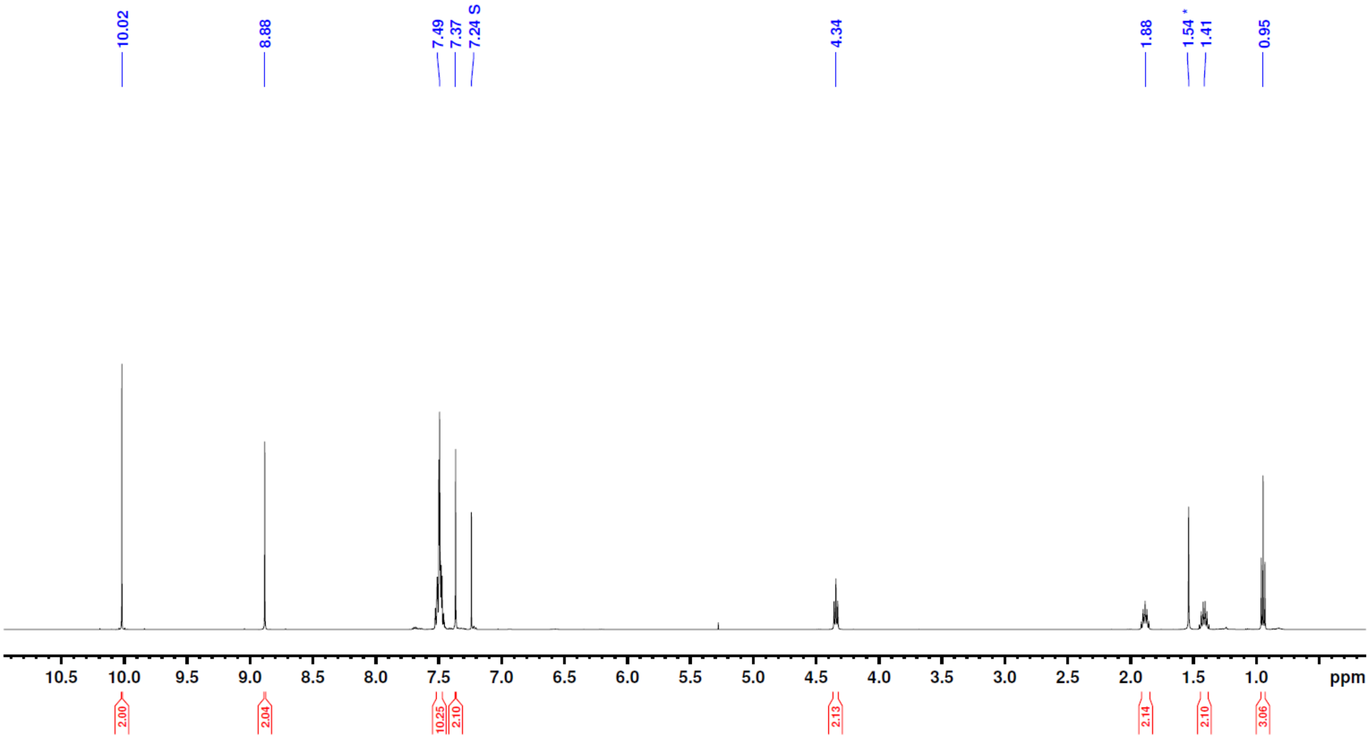

Figure S4. ^{1}H NMR spectrum of compound **4a** (500 MHz, chloroform-*d*, 300 K).

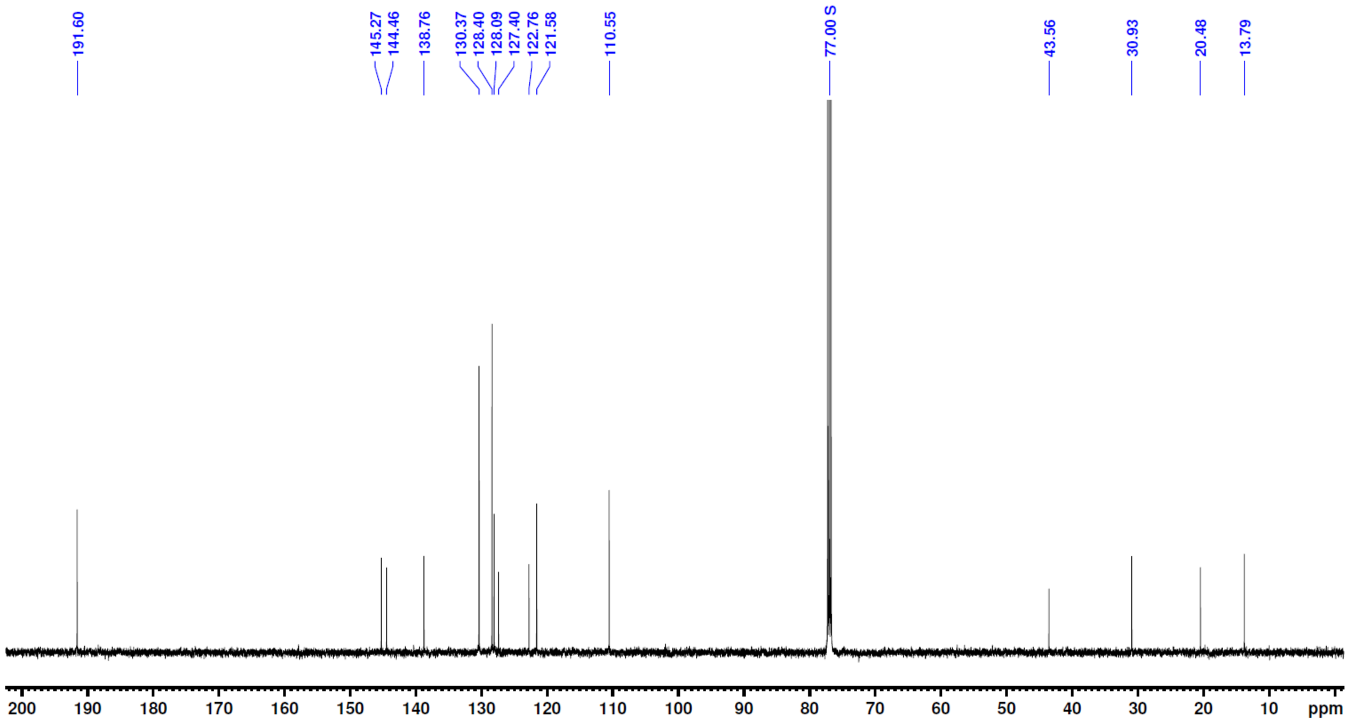

Figure S5. ^{13}C NMR spectrum of compound **4a** (125 MHz, chloroform-*d*, 300 K).

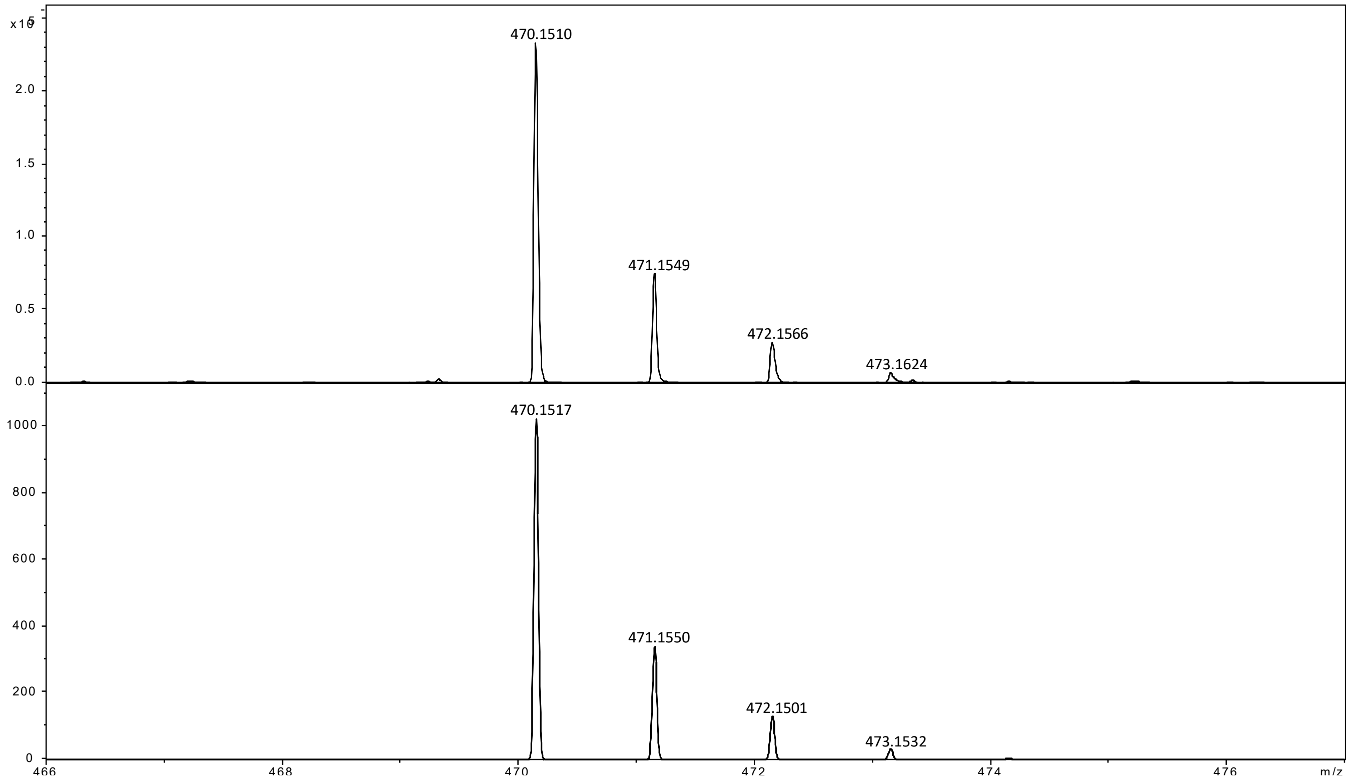

Figure S6. High resolution mass spectrum of compound **4a** (ESI–TOF, top: experimental, bottom: simulated).

6-Butyl-12,15-bis(2,4,6-triisopropylphenyl)-12,15-dihydro-6*H*-diindeno[1,2-*b:2'*,1'-*h*]carbazole (**5a**)

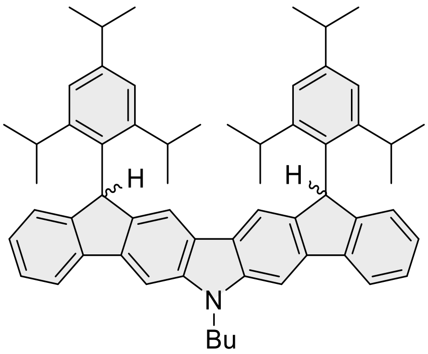

Compound **4a** (150 mg, 0.38 mmol) was dissolved in dry tetrahydrofuran (40 mL) in a flame-dried Schlenk flask and purged with nitrogen for 15 minutes. TipMgBr (4.6 mL, 0.5 M in tetrahydrofuran, 2.29 mmol) was added dropwise at room temperature and stirred overnight under nitrogen. The mixture was quenched with water, extracted with dichloromethane, dried over anhydrous sodium sulfate(VI) and evaporated to dryness. The resulting solid was dissolved in freshly distilled dichloromethane (60 mL) and purged with nitrogen for 15 minutes. 0.6 mL of boron trifluoride diethyl etherate were added slowly and stirred for 15 minutes at room temperature. The mixture was quenched with water, treated with saturated sodium bicarbonate, and extracted with dichloromethane. The organic layer was washed with brine, dried over anhydrous sodium sulfate(VI) and the solvent was removed on a rotary evaporator. The crude mixture was purified via silica column chromatography using 15% dichloromethane in *n*-hexane as an eluent to give compound **5a** as a colorless solid (237 mg, 77%, mixture of stereoisomers).

^{1}H NMR (500 MHz, chloroform-*d*, 300 K): δ 7.98 (2H, d, ^3J = 7.7 Hz), 7.84 (2H, d, ^3J = 3.2 Hz), 7.81 (2H, d, ^3J = 6.1 Hz) 7.42 (2H, m), 7.25 (4H, m), 7.19 (2H, d, ^3J = 6.6 Hz), 6.86 (2H, d, ^3J = 19.3 Hz), 5.67 (2H, s), 4.53 (2H, m), 3.68 (2H, m), 2.93 (2H, m), 2.06 (2H, m), 1.67 (1H, m), 1.60 (1H, m), 1.52 (6H, t, ^3J = 6.8 Hz), 1.48 (6H, m), 1.30 (12H, m), 1.06 (3H, m), 0.41 (6H, m), 0.36 (3H, d, ^3J = 6.9 Hz), 0.25 (3H, d, ^3J =6.9 Hz).

^{13}C NMR (125 MHz, chloroform-*d*, 300 K): δ 150.21, 150.20, 149.23, 149.06, 147.99, 147.88, 147.12, 140.91, 140.77, 140.75, 138.37, 138.33, 132.12, 131.94, 126.67, 126.63, 126.44, 124.34, 122.91, 122.85, 120.68, 120.56, 119.82, 119.79, 115.67, 115.52, 99.90, 99.84, 77.00, 47.74, 47.69, 43.21, 33.93, 33.86, 31.09, 30.87, 30.82, 28.45, 28.41, 25.13, 24.93, 24.69, 24.67, 23.99, 23.96, 23.95, 23.86, 23.50, 23.39, 23.18, 20.69, 13.98.

HRMS (ESI–TOF): *m/z*: [M + Na]$^+$ Calcd for $C_{60}H_{69}N$: 826.5322; Found 826.5305.

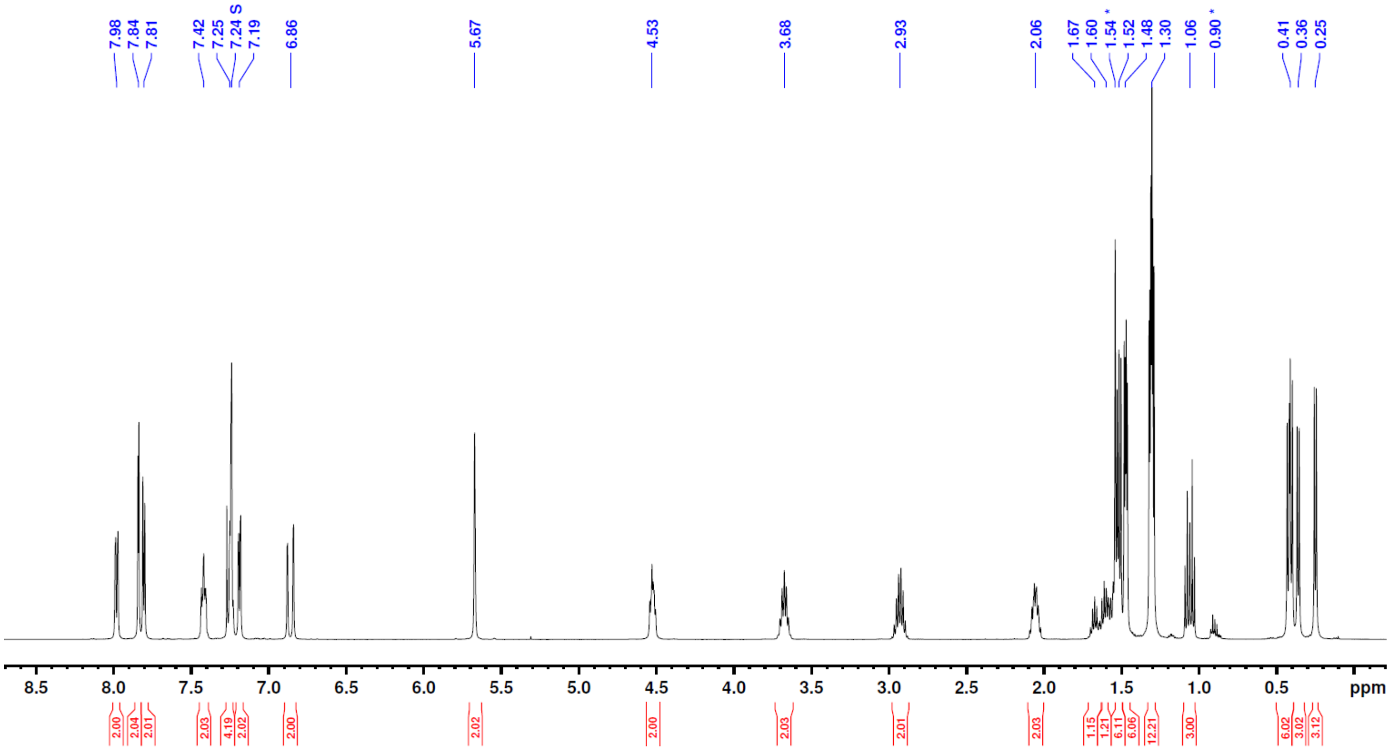

Figure S7. ^{1}H NMR spectrum of compound **5a** (500 MHz, chloroform-*d*, 300 K).

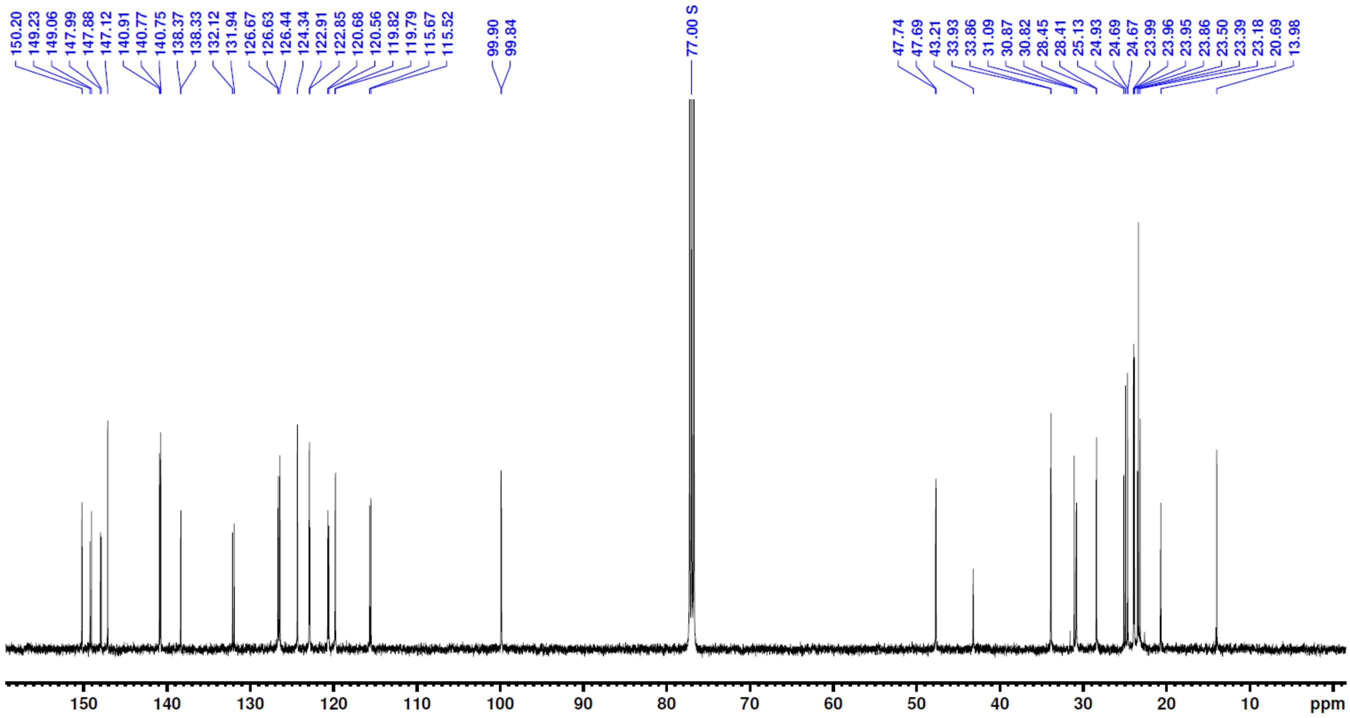

Figure S8. ^{13}C NMR spectrum of compound **5a** (125 MHz, chloroform-*d*, 300 K).

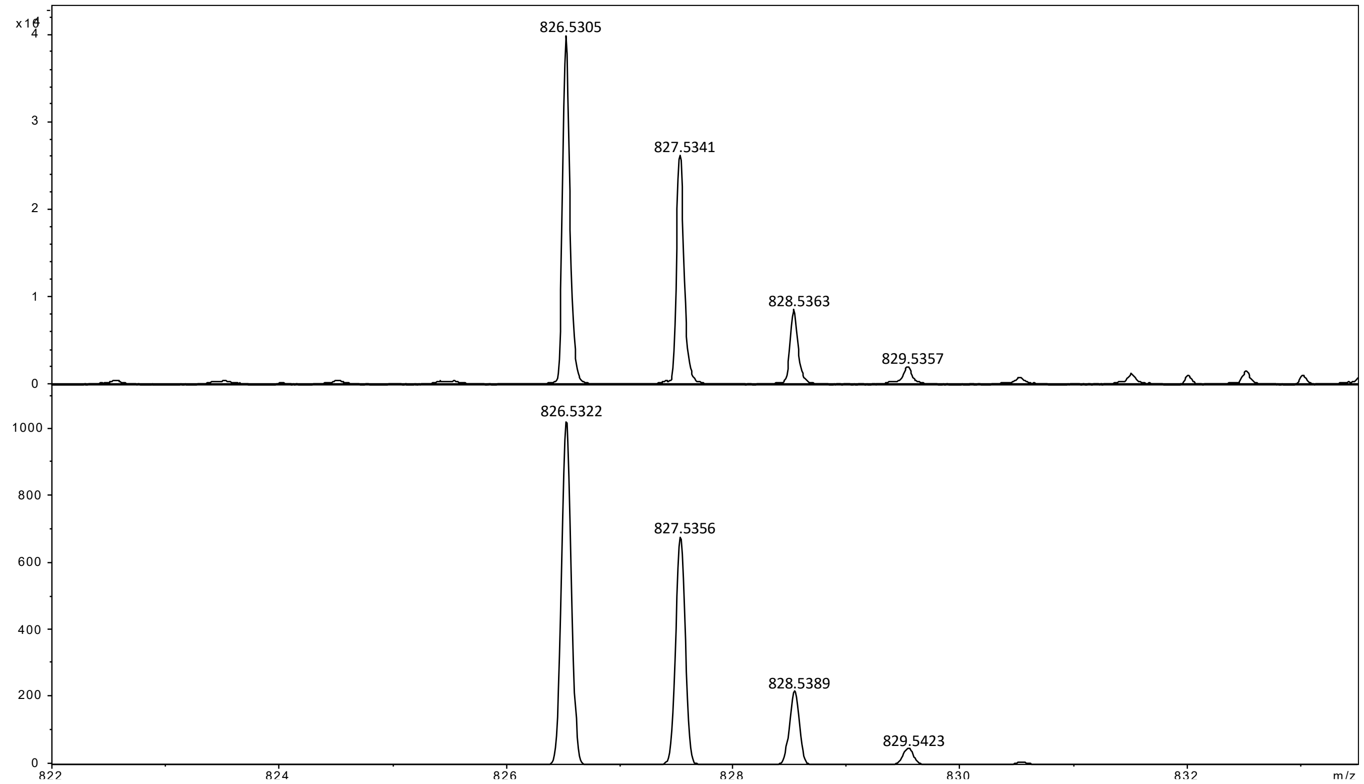

Figure S9. High resolution mass spectrum of compound **5a** (ESI–TOF, top: experimental, bottom: simulated).

6-Butyl-12,15-bis(2,4,6-triisopropylphenyl)-6*H*-diindeno[1,2-*b*:2',1'-*h*]carbazole (**1a**)

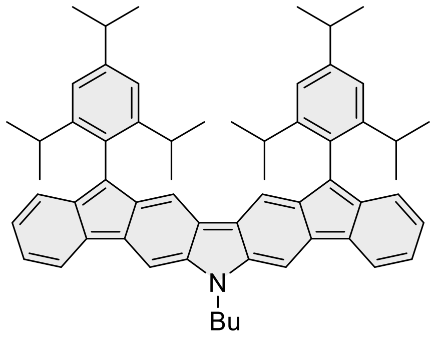

Compound **5a** (50 mg, 0.06 mmol) was dissolved in dry tetrahydrofuran (20 mL) in a flame-dried Schlenk flask and purged with nitrogen for 15 minutes. Potassium *tert*-butoxide solution (2 mol/L in 2-methyltetrahydrofuran, 187 μL, 0.37 mmol) was added slowly to the vigorously stirred solution at room temperature. The mixture was stirred for 5 minutes, and a solid copper iodide (48 mg, 0.25 mmol) was added under nitrogen. The mixture was stirred at room temperature for 15 minutes. The crude mixture was passed through celite pad and washed with methanol to afford compound **1a** as a green solid (39 mg, 78%).
HRMS (MALDI–TOF): *m/z*: $[M]^+$ Calcd for $C_{60}H_{67}N$: 801.5274; Found 801.5269.

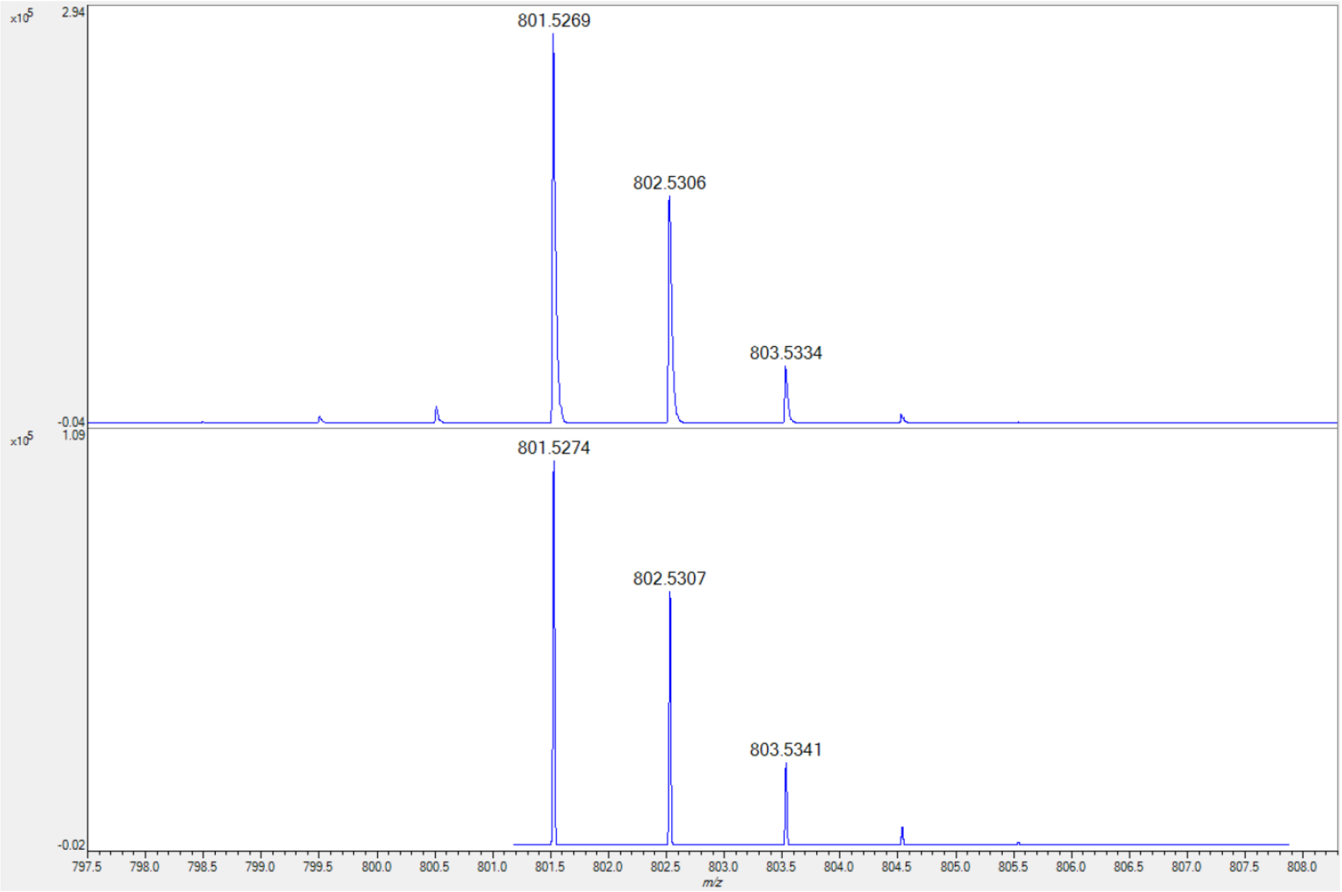

Figure S10. High resolution mass spectrum of compound **1a** (MALDI–TOF, top: experimental, bottom: simulated).

3,7-Dibromodibenzo[*b,d*]thiophene-2,8-dicarbaldehyde (**3b**)

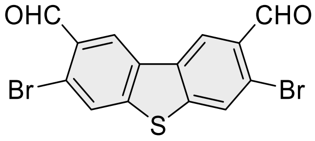

Compound **S2** (1 g, 1.68 mmol) was dissolved in 80 mL of dry tetrahydrofuran and purged with nitrogen for 15 minutes. The solution under nitrogen was cooled to -20 °C and a solution of isopropyl magnesium chloride (1.85 mL, 3.70 mmol, 2 M solution in tetrahydrofuran) was added dropwise keeping the temperature below -20 °C. The mixture was stirred for an hour under that temperature and an anhydrous *N,N* -dimethylformamide (1.3 mL, 16.80 mmol) was added dropwise. The reaction mixture was warmed to an ambient temperature and stirred for an additional 12 hours. The reaction was then quenched with aqueous ammonium chloride solution and extracted with chloroform. The organic layer was washed with brine, dried over anhydrous sodium sulfate(VI) and the solvent was removed on a rotary evaporator. The crude mixture was purified via silica column chromatography using 50% dichloromethane in *n*-hexane as an eluent to give compound **3b** (223 mg, 33%) as a beige solid with a poor solubility in organic solvents.

^{1}H NMR (500 MHz, chloroform-*d*, 300 K): δ 10.47 (2H, s), 8.74 (2H, s), 8.13 (2H, s) .

^{13}C NMR (125 MHz, chloroform-*d*, 300 K): δ 190.97, 146.34, 134.42, 130.76, 127.53, 125.00, 123.32.

HRMS (APCI–TOF): *m/z*: $[M + H]^+$ Calcd for $C_{14}H_6Br_2O_2S$: 398.8508; Found 398.8533.

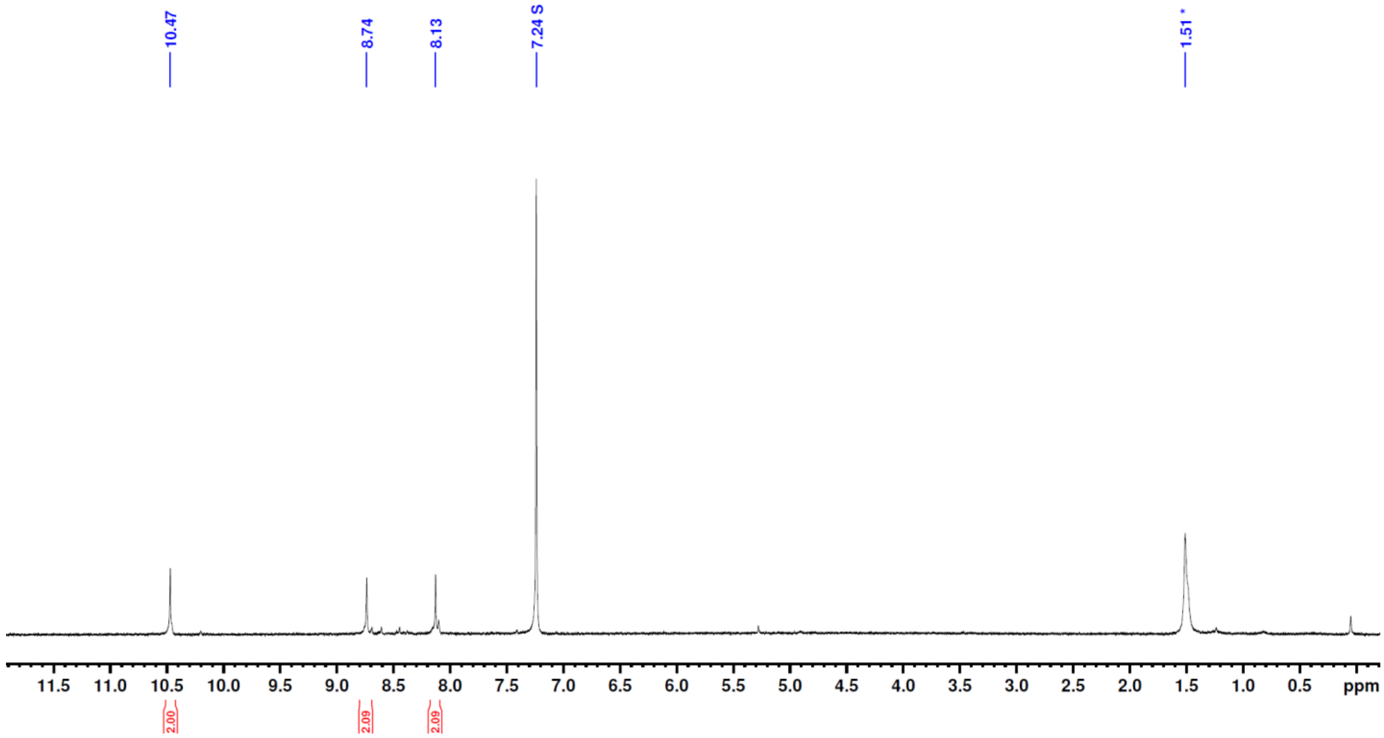

Figure S11. ^{1}H NMR spectrum of compound **3b** (500 MHz, chloroform-*d*, 300 K).

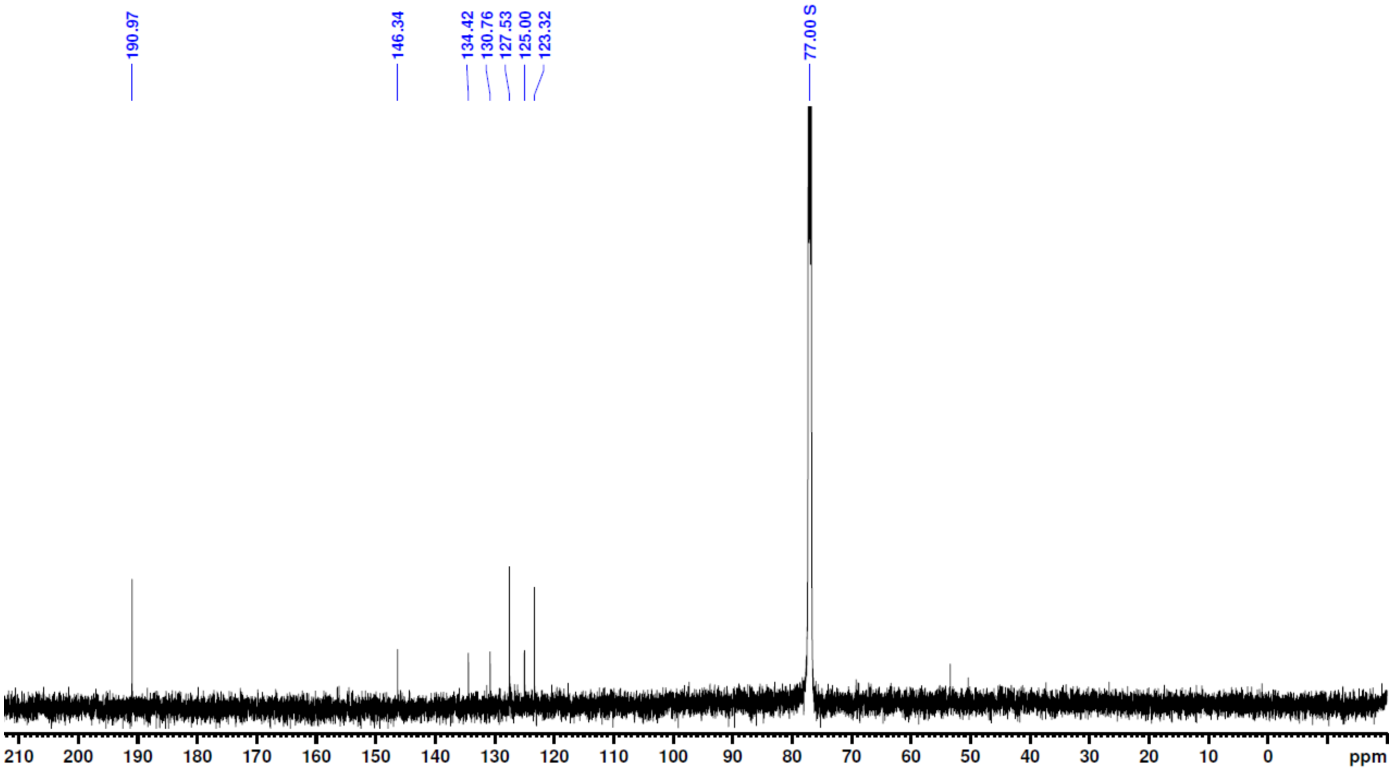

Figure S12. ^{13}C NMR spectrum of compound **3b** (125 MHz, chloroform-*d*, 300 K).

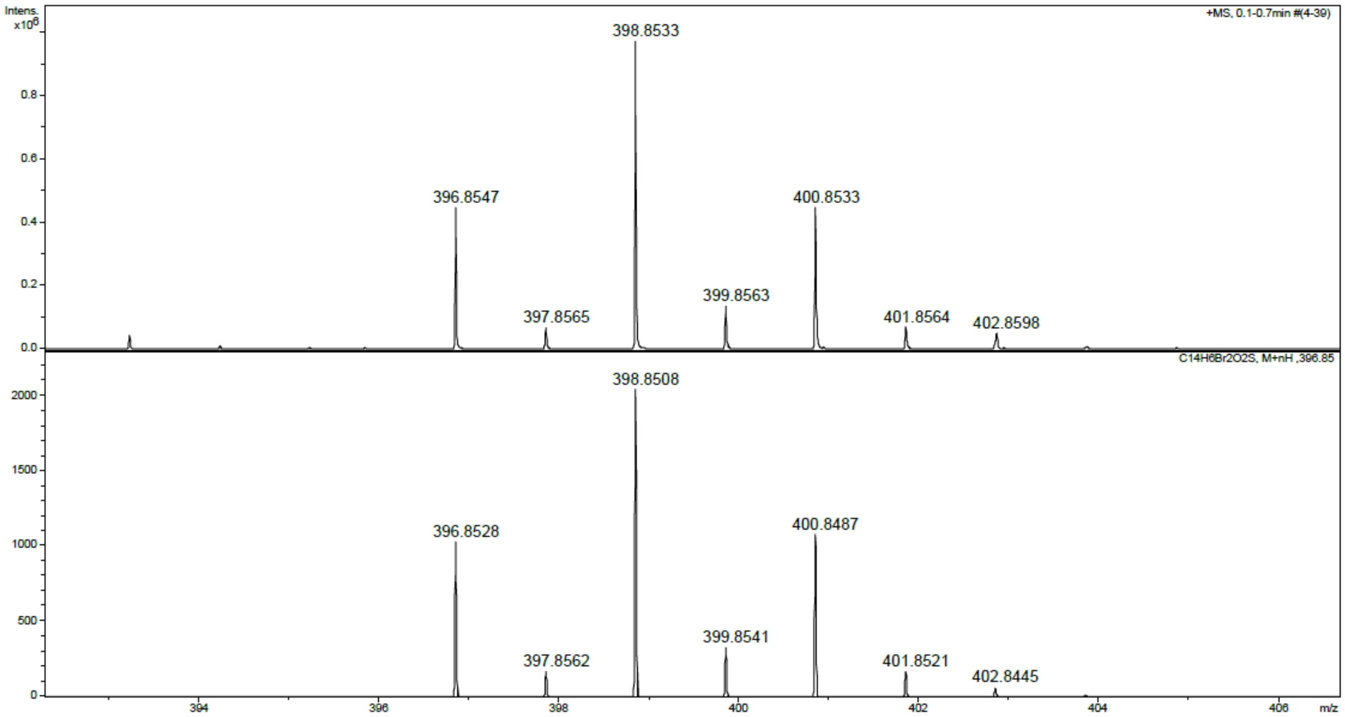

Figure S13. High resolution mass spectrum of compound **3b** (APCI–TOF, top: experimental, bottom: simulated).

3,7-Diphenyldibenzo[*b,d*]thiophene-2,8-dicarbaldehyde (**4b**)

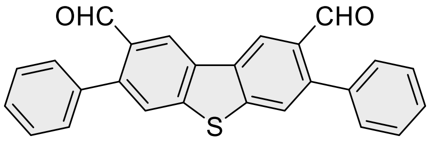

Compound **3b** (100 mg, 0.25 mmol) and phenyl boronic acid (80.61 mg, 0.63 mmol) was dissolved in 20 mL of dioxane and the solution was purged with nitrogen for 15 minutes. The degassed solution of sodium carbonate (277.75 mg, 2.01 mmol) in 2 mL of water was added to the mixture followed by the addition of tetrakis(triphenylphosphine)palladium(0) (29 mg, 0.003 mmol) The mixture was purged with nitrogen for few minutes and heated at 100 °C for 24 hours. After cooling to room temperature, water was added and extracted with dichloromethane. The organic layer was washed with brine, dried over anhydrous sodium sulfate(VI) and the solvent was removed on rotary evaporator. The crude mixture was purified via silica column chromatography using 60% dichloromethane in *n*-hexane as an eluent to give compound **4b** (83 mg, 84%) as a beige solid.

^{1}H NMR (500 MHz, chloroform-*d*, 300 K): δ 10.08 (2H, s), 8.92 (2H, s), 7.91 (2H, s), 7.48 (10H, m).

^{13}C NMR (125 MHz, chloroform-*d*, 300 K): δ 191.64, 145.19, 144.60, 137.42, 134.67, 131.37, 130.23, 128.61, 128.45, 124.66, 121.68.

HRMS (APCI–TOF): *m/z*: $[M + H]^+$ Calcd for $C_{26}H_{16}O_2S$: 393.0944; Found 393.0974.

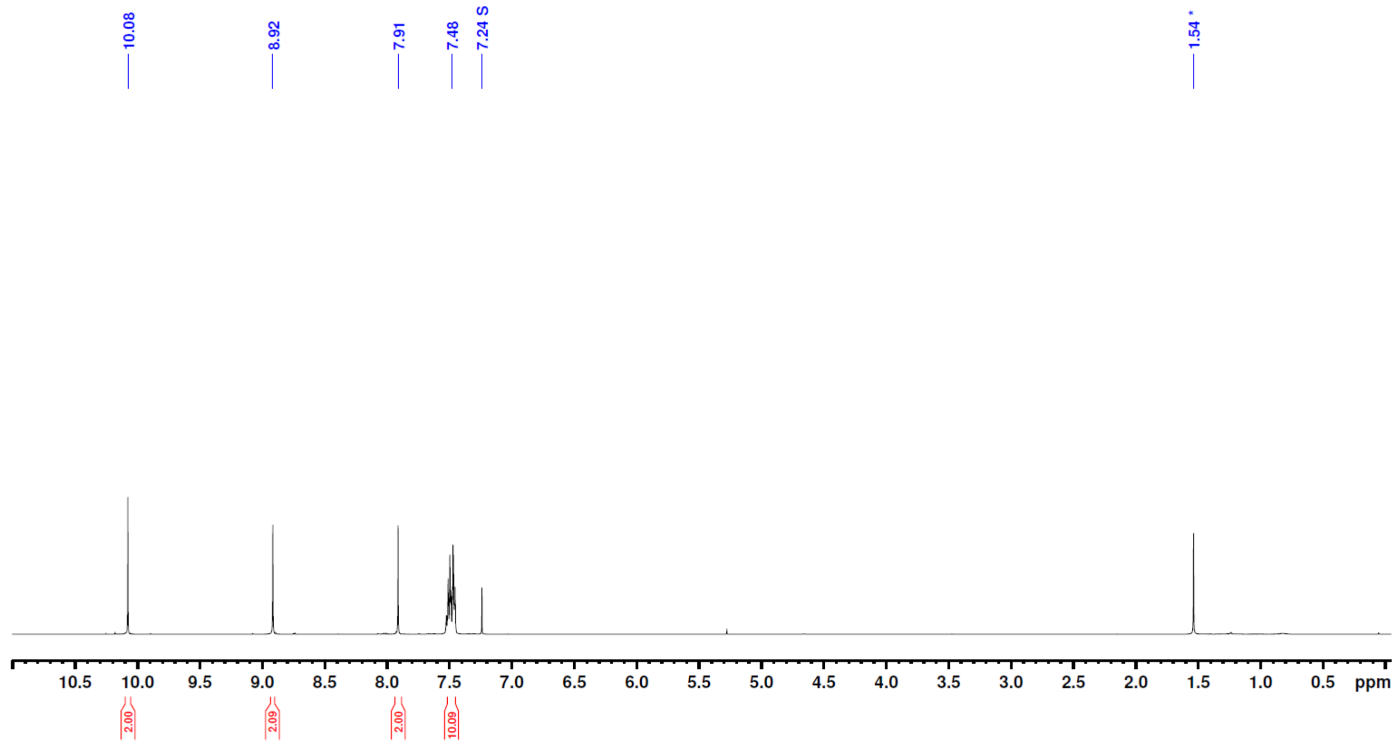

Figure S14. ^{1}H NMR spectrum of compound **4b** (500 MHz, chloroform-*d*, 300 K).

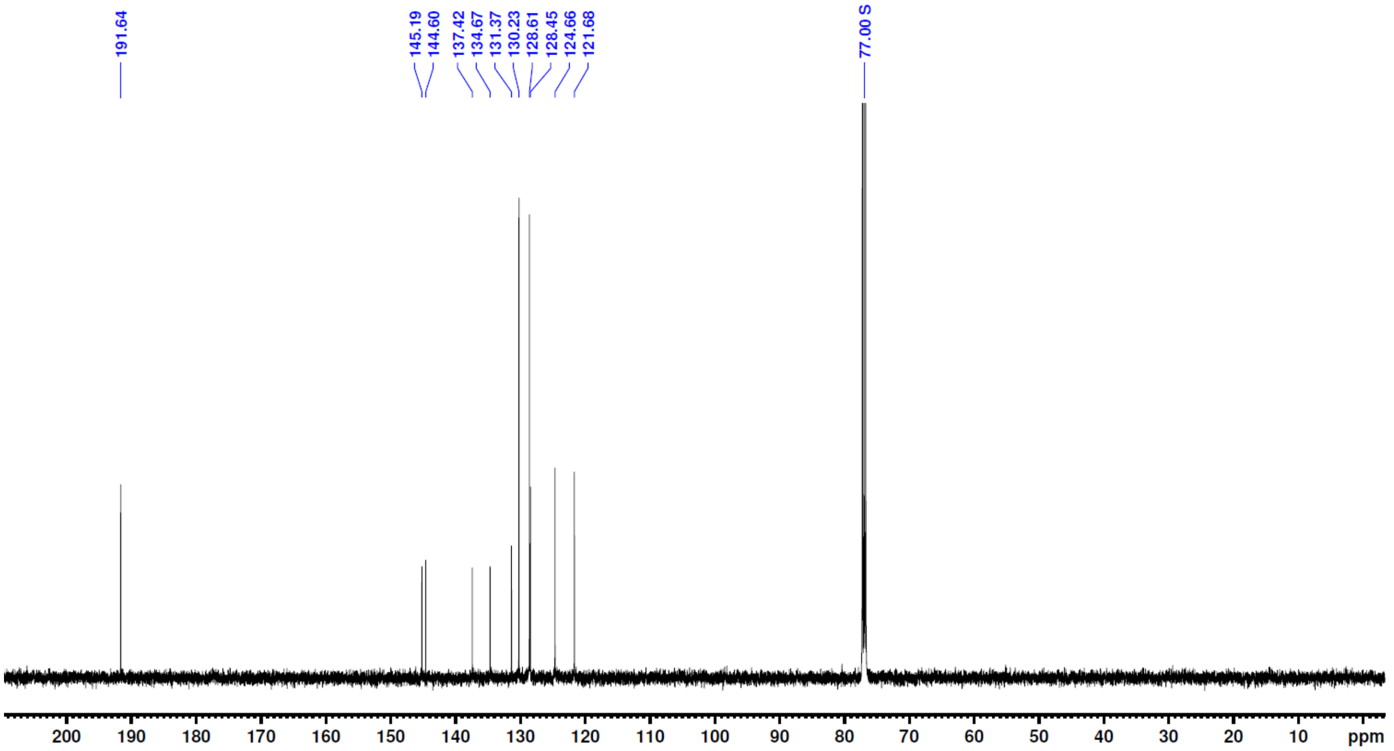

Figure S15. ^{13}C NMR spectrum of compound **4b** (125 MHz, chloroform-*d*, 300 K).

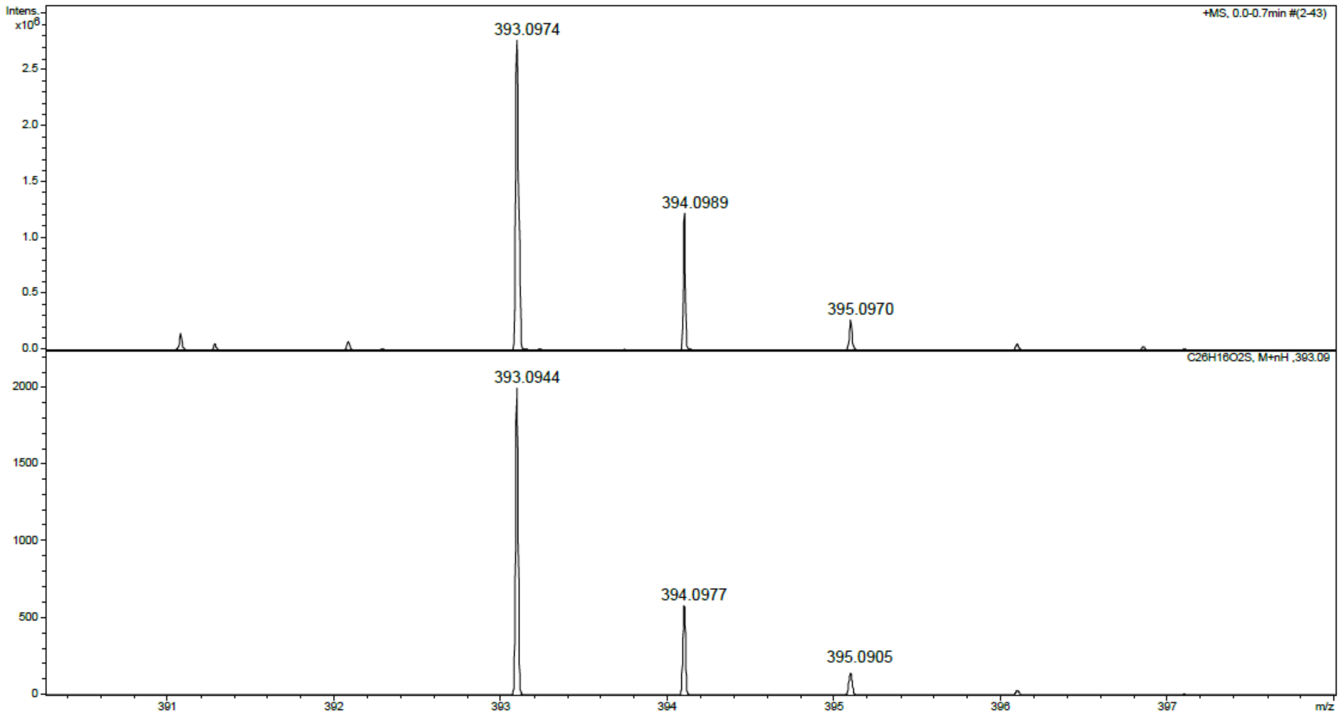

Figure S16. High resolution mass spectrum of compound **4b** (APCI–TOF, top: experimental, bottom: simulated).

12,15-Bis(2,4,6-triisopropylphenyl)-12,15-dihydrodifluoreno[3,2-*b*:2',3'-*d*]thiophene (**5b**)

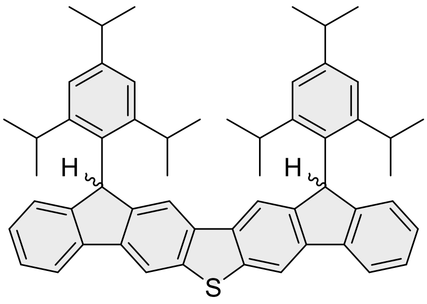

Compound **4b** (70 mg, 0.18 mmol) was dissolved in dry tetrahydrofuran (15 mL) in a flame-dried Schlenk flask and purged with nitrogen for 15 minutes. TipMgBr (2.1 mL, 0.5 M in tetrahydrofuran, 1.07 mmol) was added dropwise at room temperature and stirred overnight under nitrogen. The mixture was quenched with water, extracted with dichloromethane, dried over anhydrous sodium sulfate(VI) and evaporated to dryness. The resulting solid was dissolved in freshly distilled dichloromethane (20 mL) and purged with nitrogen for 15 minutes. 0.2 mL of boron trifluoride diethyl etherate were added slowly and stirred for 15 minutes at room temperature. The mixture was quenched with water, treated with saturated sodium bicarbonate, and extracted with dichloromethane. The organic layer was washed with brine, dried over anhydrous sodium sulfate(VI) and the solvent was removed on a rotary evaporator The crude mixture was purified via silica column chromatography using 20% dichloromethane in *n*-hexane as an eluent to give compound **5b** as a colorless solid (104 mg, 76 %, mixture of stereoisomers).

^{1}H NMR (500 MHz, chloroform-*d*, 300 K): δ 8.28 (2H, d, ^3J = 2.3 Hz), 7.89 (4H, m), 7.39 (2H, m), 7.24 (2H, m), 7.18 (4H, m), 6.84 (2H, dd, ^3J = 14.4 Hz, ^4J = 1.8 Hz), 5.62 (2H, s), 3.60 (2H, m), 2.91 (2H, m), 1.56 (2H, m), 1.47 (6H, d, ^3J = 6.9 Hz), 1.43 (6H, t, ^3J = 5.9 Hz), 1.27 (12H, m), 0.37 (9H, m), 0.28 (3H, d, ^3J = 6.9 Hz).

^{13}C NMR (125 MHz, chloroform-*d*, 300 K): δ 149.89, 149.16, 149.06, 147.98, 147.89, 147.48, 147.43, 146.61, 146.46, 139.59, 139.49, 138.57, 134.92, 131.36, 131.16, 127.30, 126.7,7 124.42, 124.40, 123.09, 122.96, 120.95, 120.79, 120.25, 120.18, 116.9,8 116.95, 114.28, 114.26, 48.18, 48.12, 33.96, 33.81, 30.91, 30.81, 28.63, 28.60, 25.18, 24.98, 24.61, 24.51, 24.01, 23.86, 23.71, 23.47, 23.42, 23.33, 23.31.

HRMS (MALDI–TOF): *m/z*: $[M]^+$ Calcd for $C_{56}H_{60}S$: 764.4416; Found 764.4406.

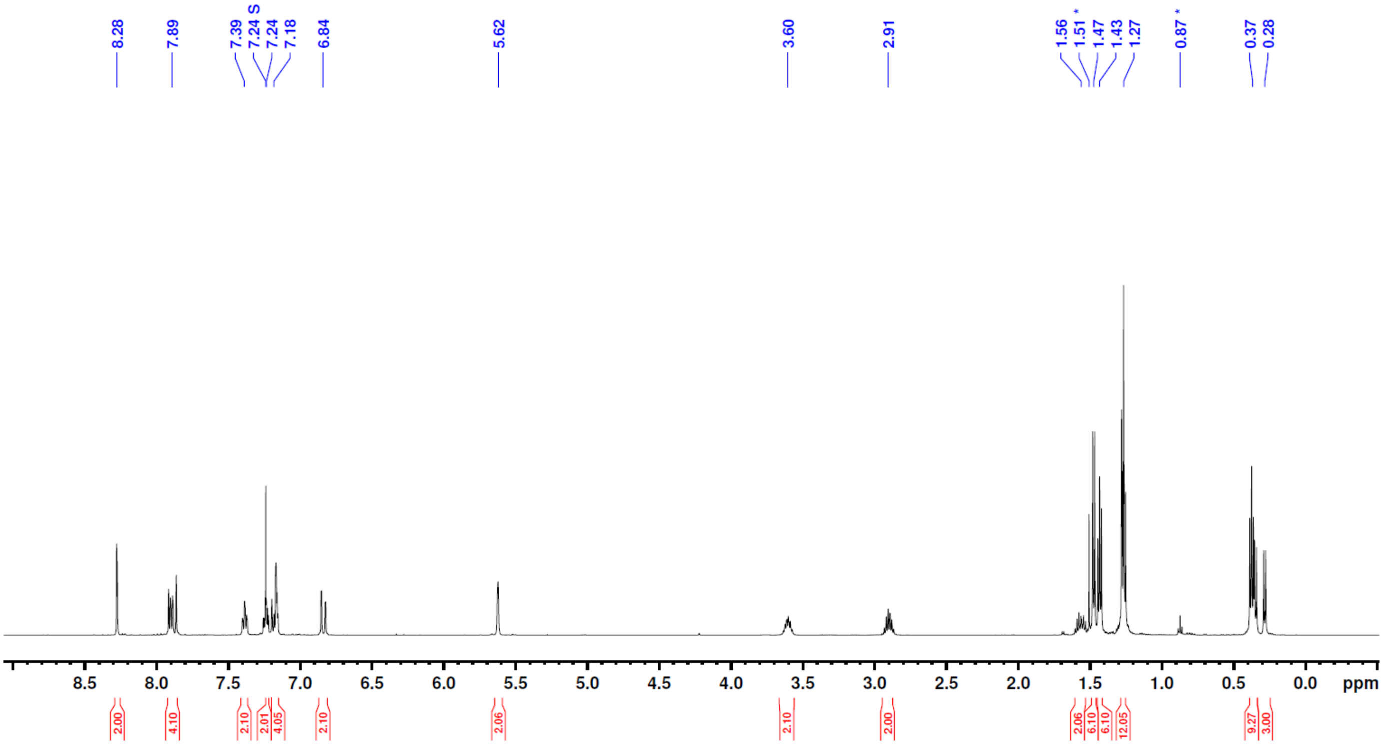

Figure S17. ^{1}H NMR spectrum of compound **5b** (500 MHz, chloroform-*d*, 300 K).

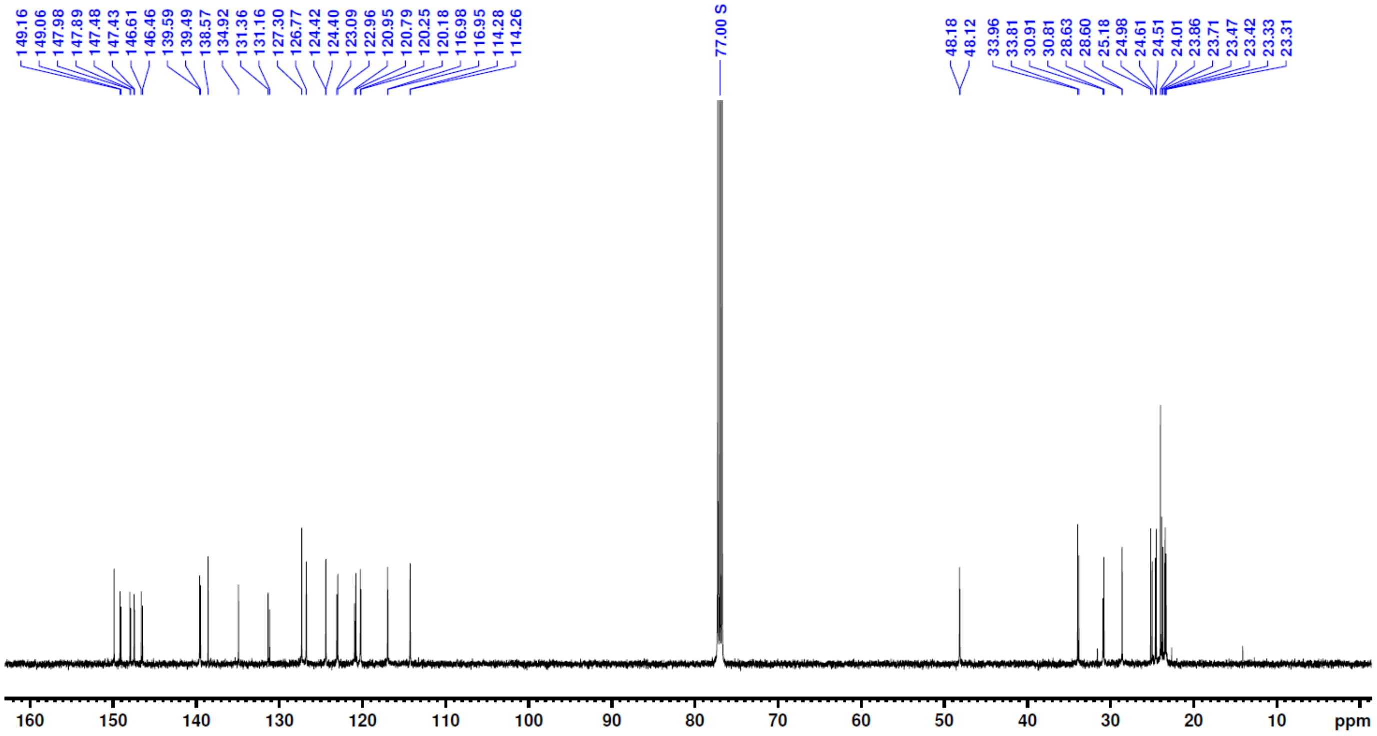

Figure S18. ^{13}C NMR spectrum of compound **5b** (125 MHz, chloroform-*d*, 300 K).

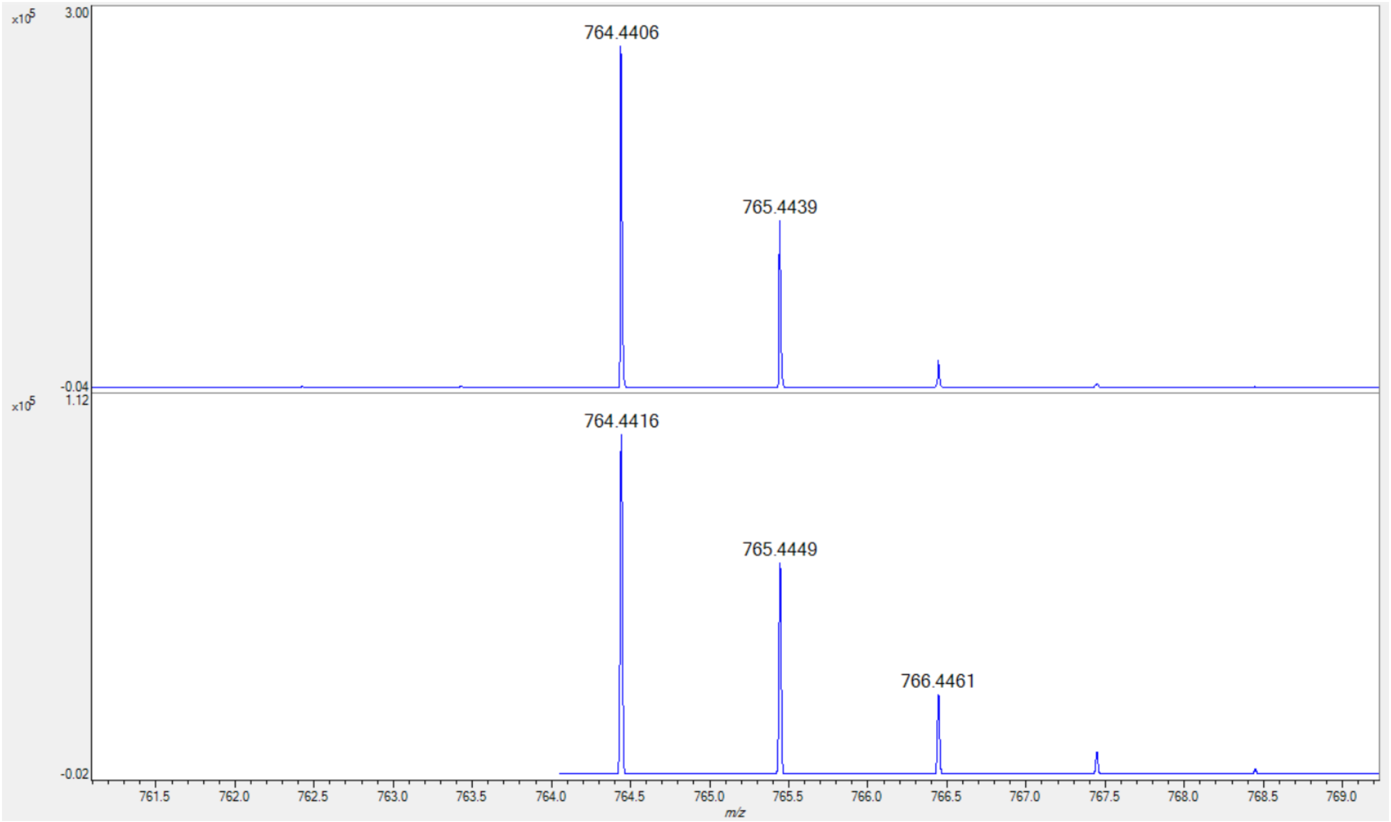

Figure S19. High resolution mass spectrum of compound **5b** (MALDI–TOF, top: experimental, bottom: simulated).

12,15-Bis(2,4,6-triisopropylphenyl) difluoreno[3,2-*b*:2',3'-*d*]thiophene (**1b**)

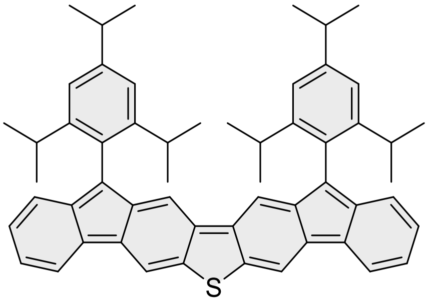

Compound **5b** (40 mg, 52 µmol) was dissolved in dry tetrahydrofuran (25 mL) in a flame-dried Schlenk flask and purged with nitrogen for 15 minutes. Potassium tert-butoxide solution (2 mol/L in 2-methyltetrahydrofuran, 160 µL, 0.31 mmol) was added slowly to the vigorously stirred solution at room temperature. The mixture was stirred for 5 minutes, and solid copper iodide (51 mg, 0.21 mmol) was added to the mixture under nitrogen. The dark-red mixture was stirred at room temperature for 15 minutes. The mixture was passed through a celite, and the solvent was removed on a rotary evaporator. The crude mixture was washed with methanol to afford compound **1b** as a brown solid (29 mg, 73%).

HRMS (MALDI–TOF): *m*/*z*: $[M]^+$ Calcd for $C_{56}H_{58}S$: 762.4259; Found 762.4280.

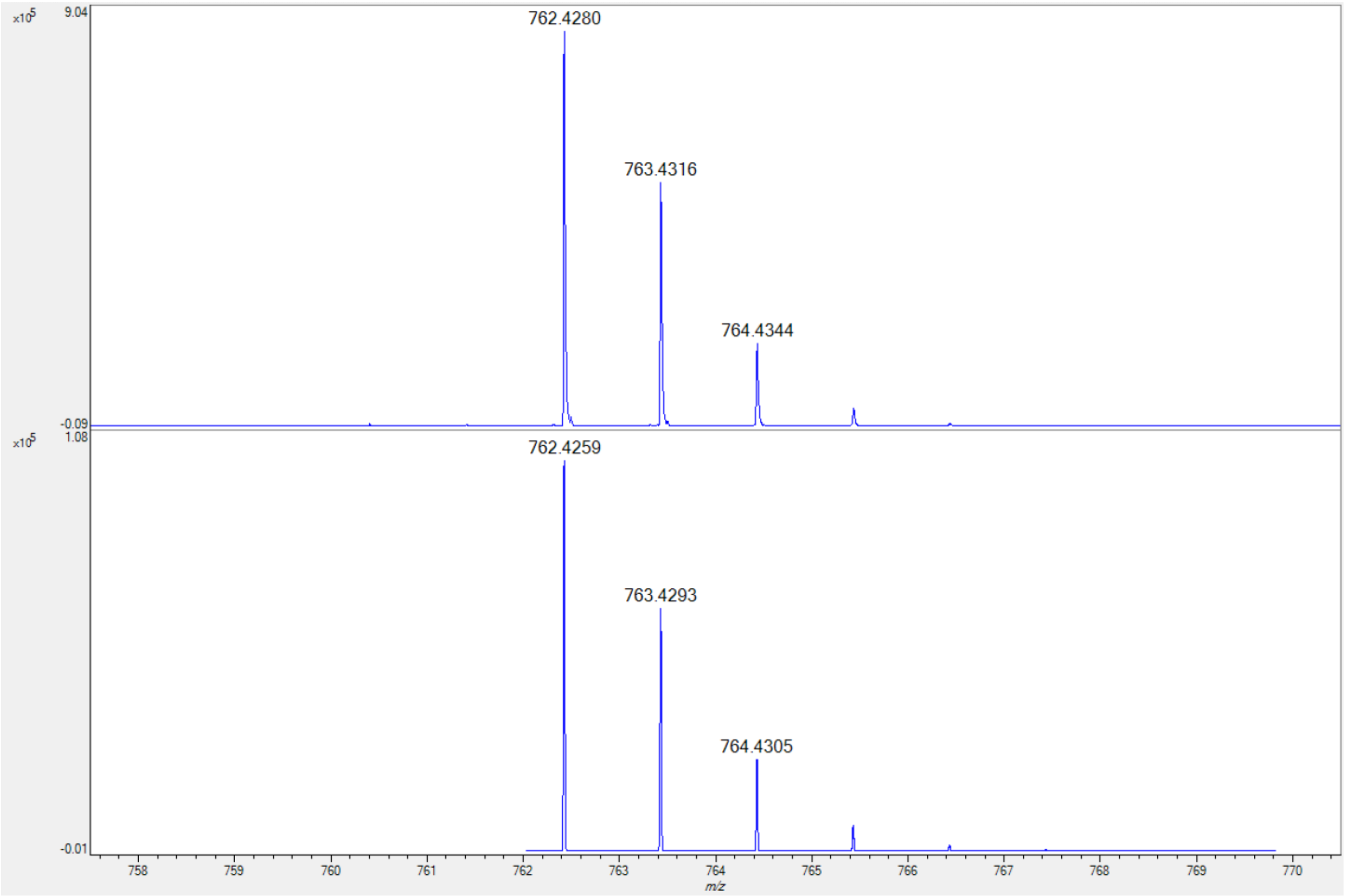

Figure S20. High resolution mass spectrum of compound **1b** (MALDI–TOF, top: experimental, bottom: simulated).

9-Butyl-3,6-diphenyl-9*H*-carbazole-2,7-dicarbaldehyde (**7a**)

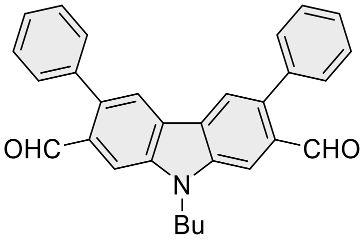

Compound **6a** (200 mg, 0.46 mmol) and phenyl boronic acid (146.81 mg, 1.14 mmol) was dissolved in 50 mL of dioxane and the solution was purged with nitrogen for 15 minutes. The degassed solution of sodium carbonate (387.95 g, 3.66 mmol) in 5 mL of water was added to the mixture followed by the addition of tetrakis(triphenylphosphine)palladium(0) (52.87 mg, 0.05 mmol) The mixture was purged with nitrogen for few minutes and heated at 100 °C for 24 hours. After cooling to room temperature, water was added and extracted with dichloromethane. The organic layer was washed with brine, dried over anhydrous sodium sulfate(VI) and the solvent was removed on rotary evaporator. The crude mixture was purified via silica column chromatography using 50% dichloromethane in *n*-hexane as an eluent to give compound **7a** (183 mg, 93%) as a yellow solid.

^{1}H NMR (500 MHz, chloroform-*d*, 300 K): δ 10.10 (2H, s), 8.17 (2H, s), 8.15 (2H, s), 7.46 (10H, m), 4.47 (2H, t, ^3J = 7.4 Hz), 1.93 (2H, m), 1.46 (2H, m), 0.98 (3H, t, ^3J = 7.4 Hz).

^{13}C NMR (125 MHz, chloroform-*d*, 300 K): δ 192.71, 141.37, 138.48, 137.58, 132.77, 130.62, 128.40, 127.68, 126.22, 123.36, 108.17, 43.58, 31.30, 20.56, 13.85.

HRMS (ESI–TOF): *m/z*: [M + H]$^+$ Calcd for $C_{30}H_{25}NO_2$: 432.1958; Found 432.1981.

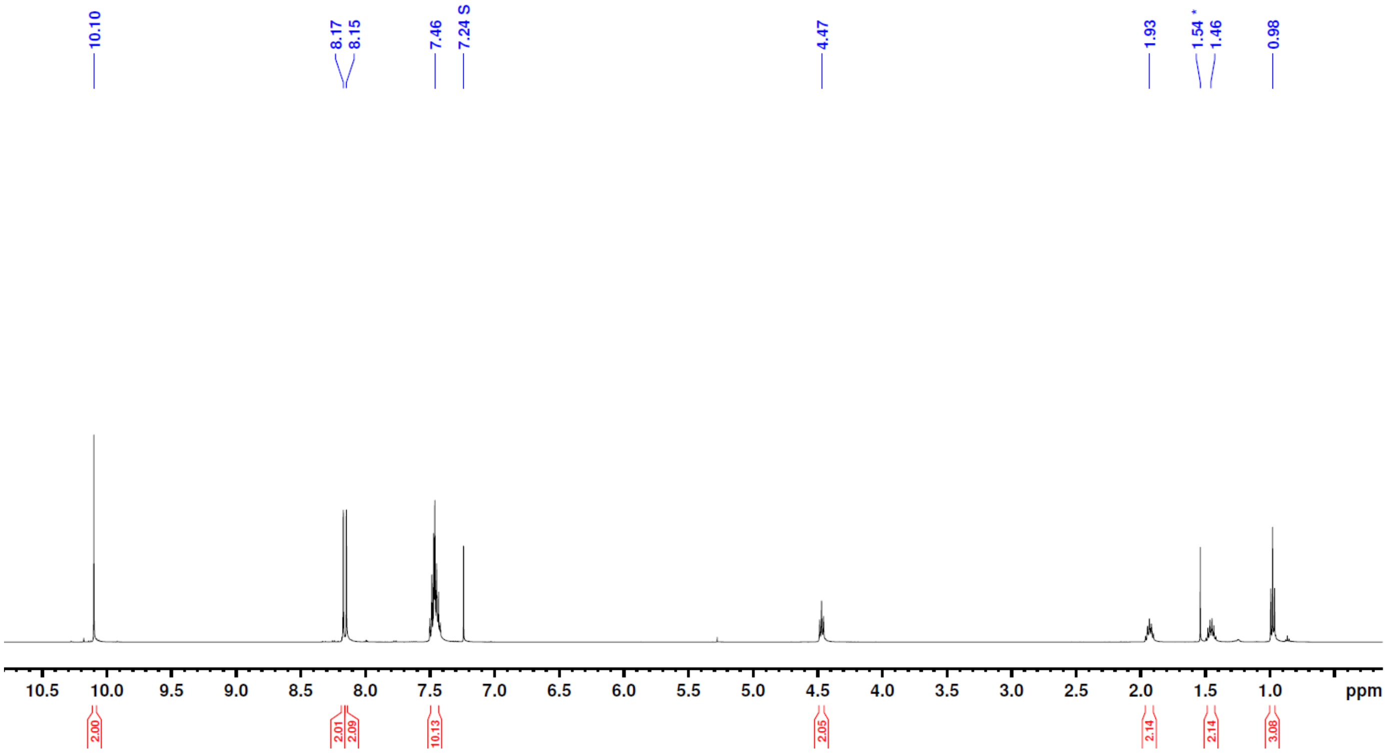

Figure S21. ^{1}H NMR spectrum of compound **7a** (500 MHz, chloroform-*d*, 300 K).

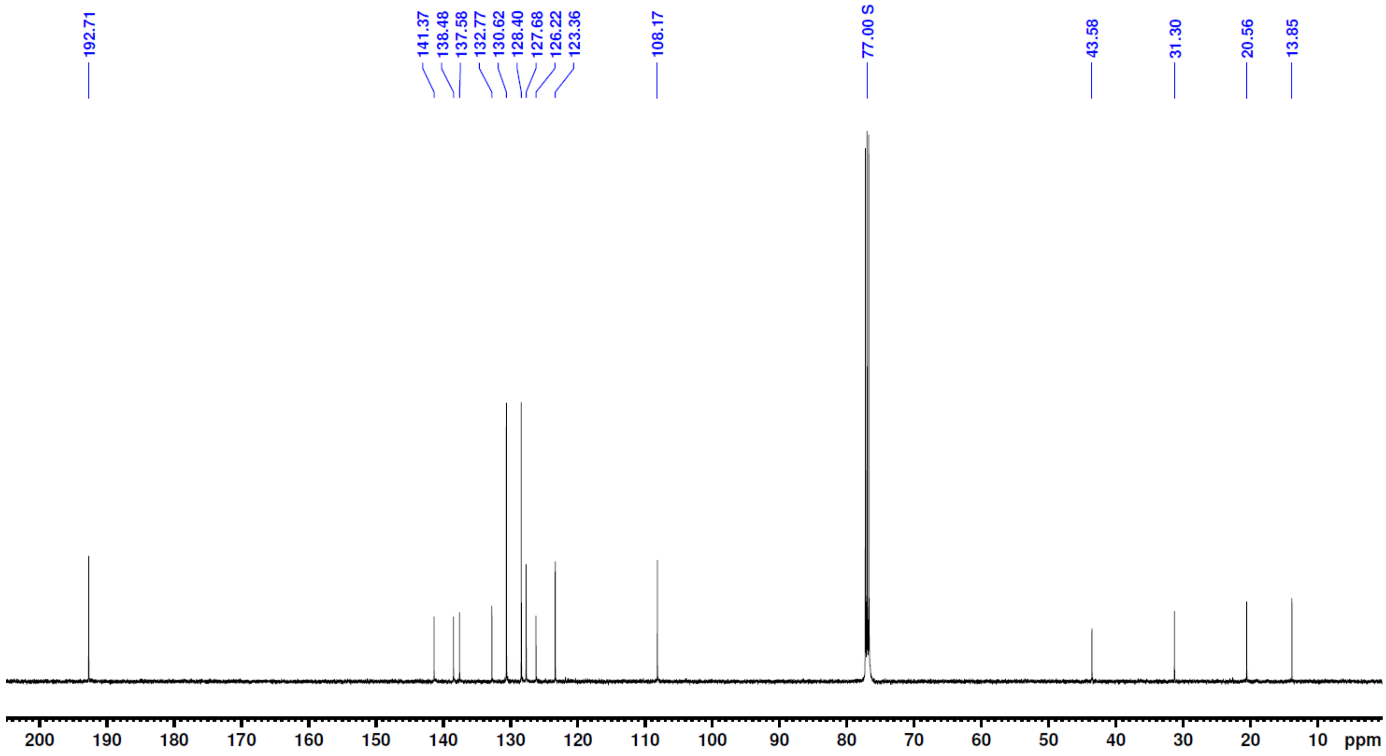

Figure S22. ^{13}C NMR spectrum of compound **7a** (125 MHz, chloroform-*d*, 300 K).

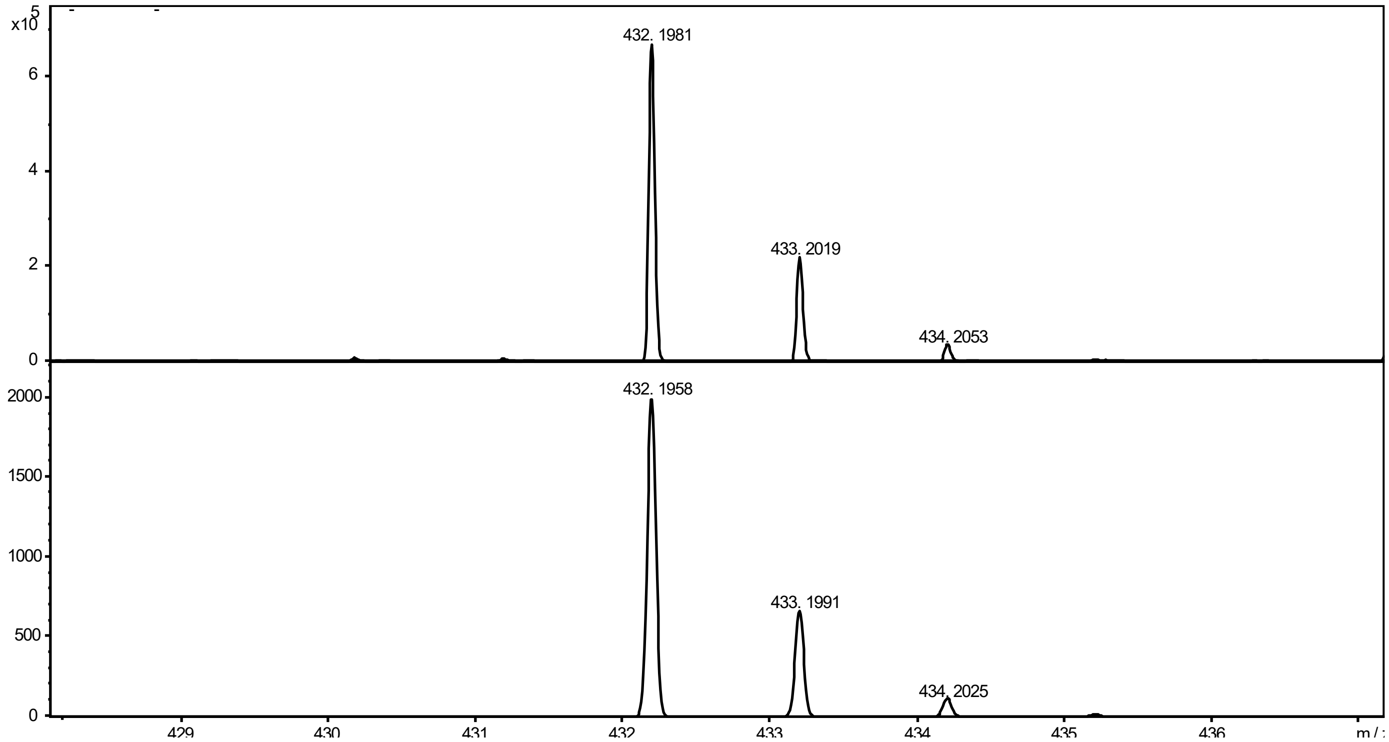

Figure S23. High resolution mass spectrum of compound **7a** (ESI–TOF, top: experimental, bottom: simulated).

7-Butyl-5,9-bis(2,4,6-triisopropylphenyl)-7,9-dihydro-5*H*-diindeno[2,1-*b*:1',2'-*h*]carbazole (**8a**)

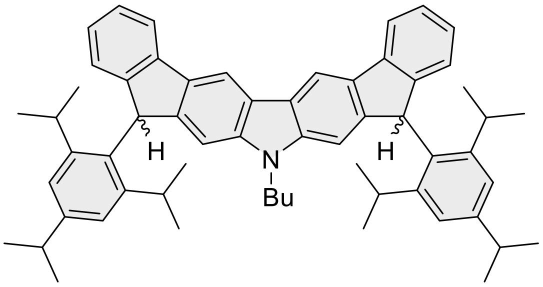

Compound **7a** (150 mg, 0.35 mmol) was dissolved in dry tetrahydrofuran (40 mL) in a flame-dried Schlenk flask and purged with nitrogen for 15 minutes. TipMgBr (4.2 mL, 0.5 M in tetrahydrofuran, 2.09 mmol) was added dropwise at room temperature and stirred overnight under nitrogen. The mixture was quenched with water, extracted with dichloromethane, dried over anhydrous sodium sulfate(VI) and evaporated to dryness. The resulting solid was dissolved in freshly distilled dichloromethane (60 mL) and purged with nitrogen for 15 minutes. 0.6 mL of boron trifluoride diethyl etherate were added slowly and stirred for 15 minutes at room temperature. The mixture was quenched with water, treated with saturated sodium bicarbonate, and extracted with dichloromethane. The organic layer was washed with brine, dried over anhydrous sodium sulfate(VI) and the solvent was removed on a rotary evaporator. The crude mixture was purified via silica column chromatography using 25 % dichloromethane in *n*-hexane as an eluent to give compound **8a** as a colorless solid (207 mg, 74 %, mixture of stereoisomers).

^{1}H NMR (500 MHz, chloroform-*d*, 300 K): δ 8.62 (2H, s), 7.97 (2H, t, ^3J = 6.7 Hz), 7.40 (2H, m), 7.18 (4H, t, ^3J = 4.3 Hz), 7.15 (2H, bs), 7.13 (2H, s), 6.86 (2H, bs), 5.66 (2H, s), 4.16 (2H, m), 3.63 (2H, m), 2.91 (2H, m), 1.70-1.64 (4H, m), 1.46 (12H, m), 1.28 (12H, d, ^3J = 6.7 Hz), 1.12 (2H, m), 0.70 (3H, m), 0.40 (6H, t, ^3J = 5.2 Hz), 0.38 (3H, d, ^3J = 6.7 Hz), 0.33 (3H, d, ^3J = 6.7 Hz).

^{13}C NMR (125 MHz, chloroform-*d*, 300 K): δ 149.34, 149.33, 148.77, 148.72, 148.31, 148.21, 147.91, 147.89, 147.23, 147.21, 141.20, 141.16, 140.89, 140.87, 132.26, 132.23, 131.97, 131.93, 126.61, 125.70, 125.67, 124.10, 122.93, 122.91, 122.35, 120.63, 119.37, 119.26, 111.52, 111.49, 104.34, 48.52, 48.49, 42.51, 33.98, 30.93, 30.86, 30.82, 28.54, 24.90, 24.82, 24.75, 24.64, 24.08, 23.88, 23.53, 23.39, 23.37, 23.33, 20.09, 20.07, 13.59, 13.55.

HRMS (MALDI–TOF): *m/z*: $[M]^+$ Calcd for $C_{60}H_{69}N$: 803.5430; Found 803.5483.

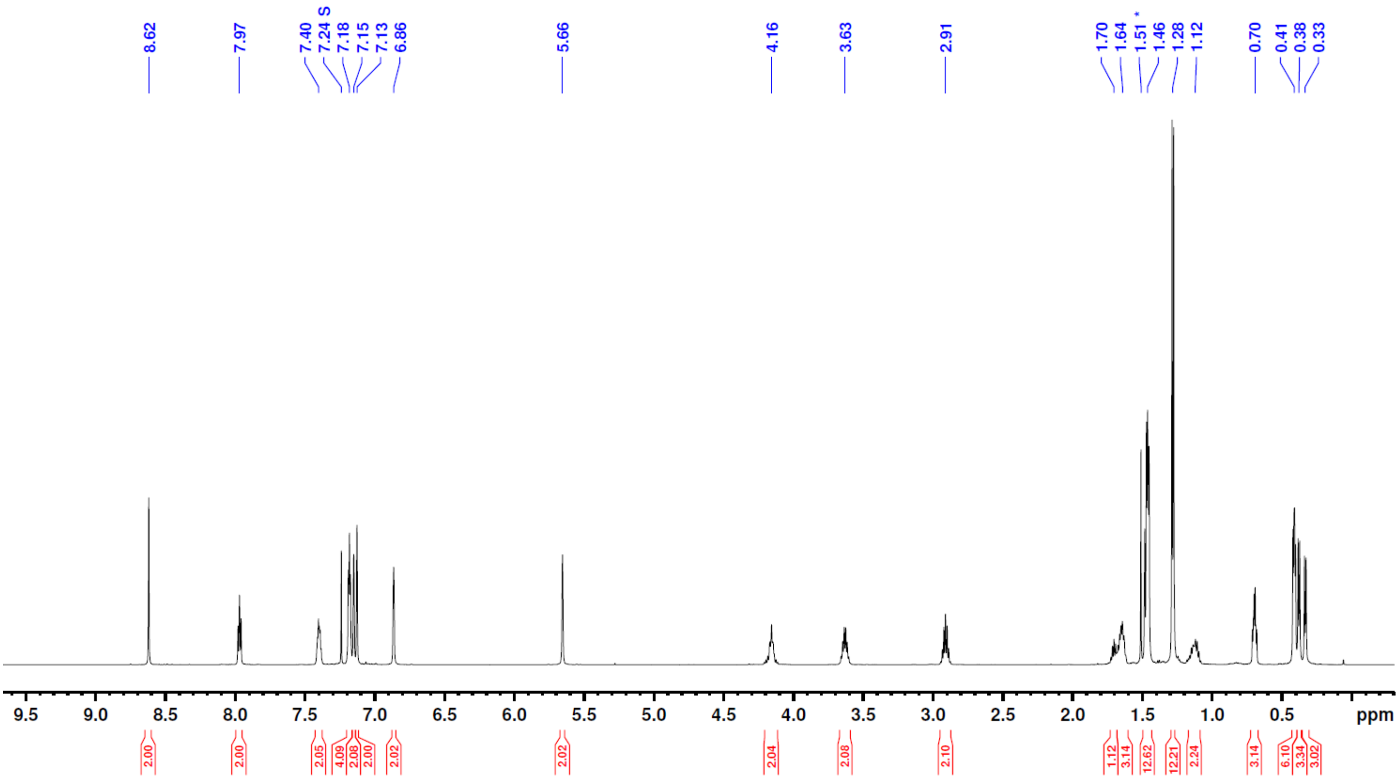

Figure S24. ^{1}H NMR spectrum of compound **8a** (500 MHz, chloroform-*d*, 300 K).

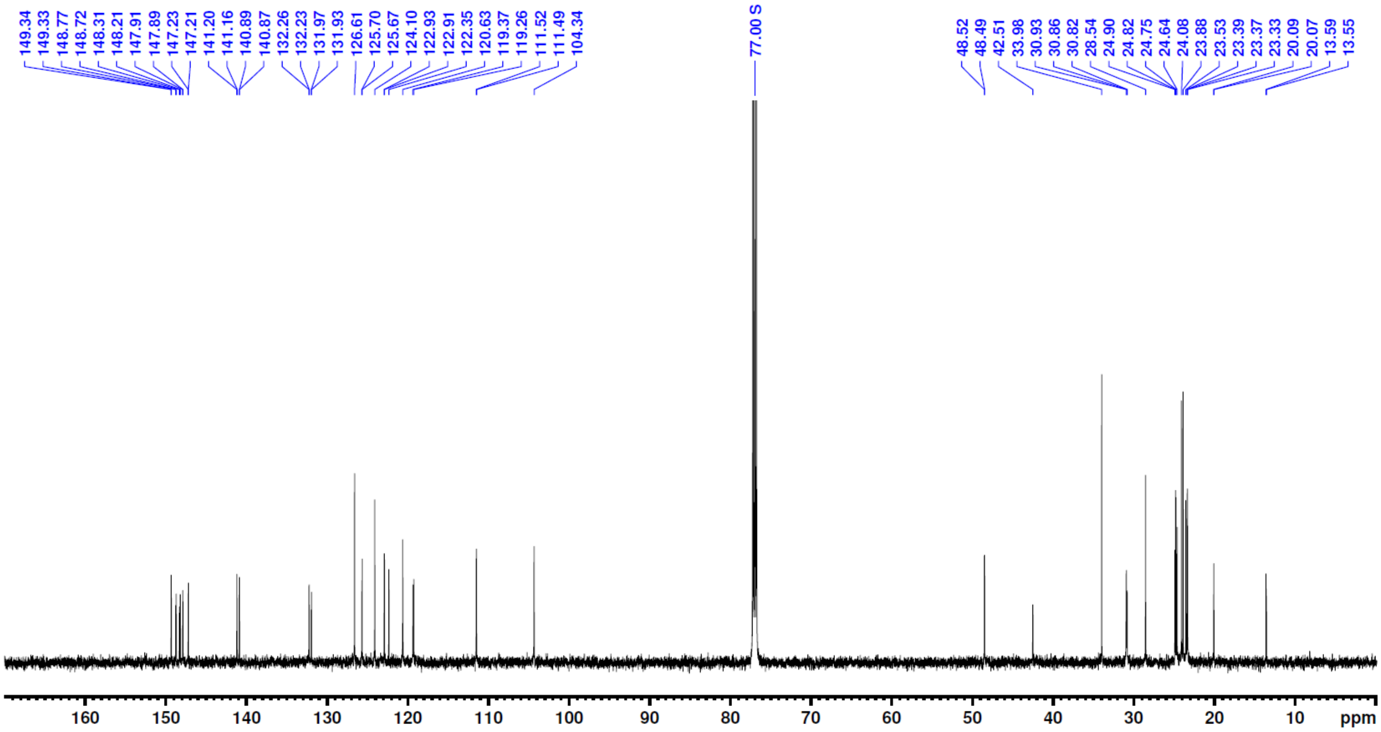

Figure S25. ^{13}C NMR spectrum of compound **8a** (125 MHz, chloroform-*d*, 300 K).

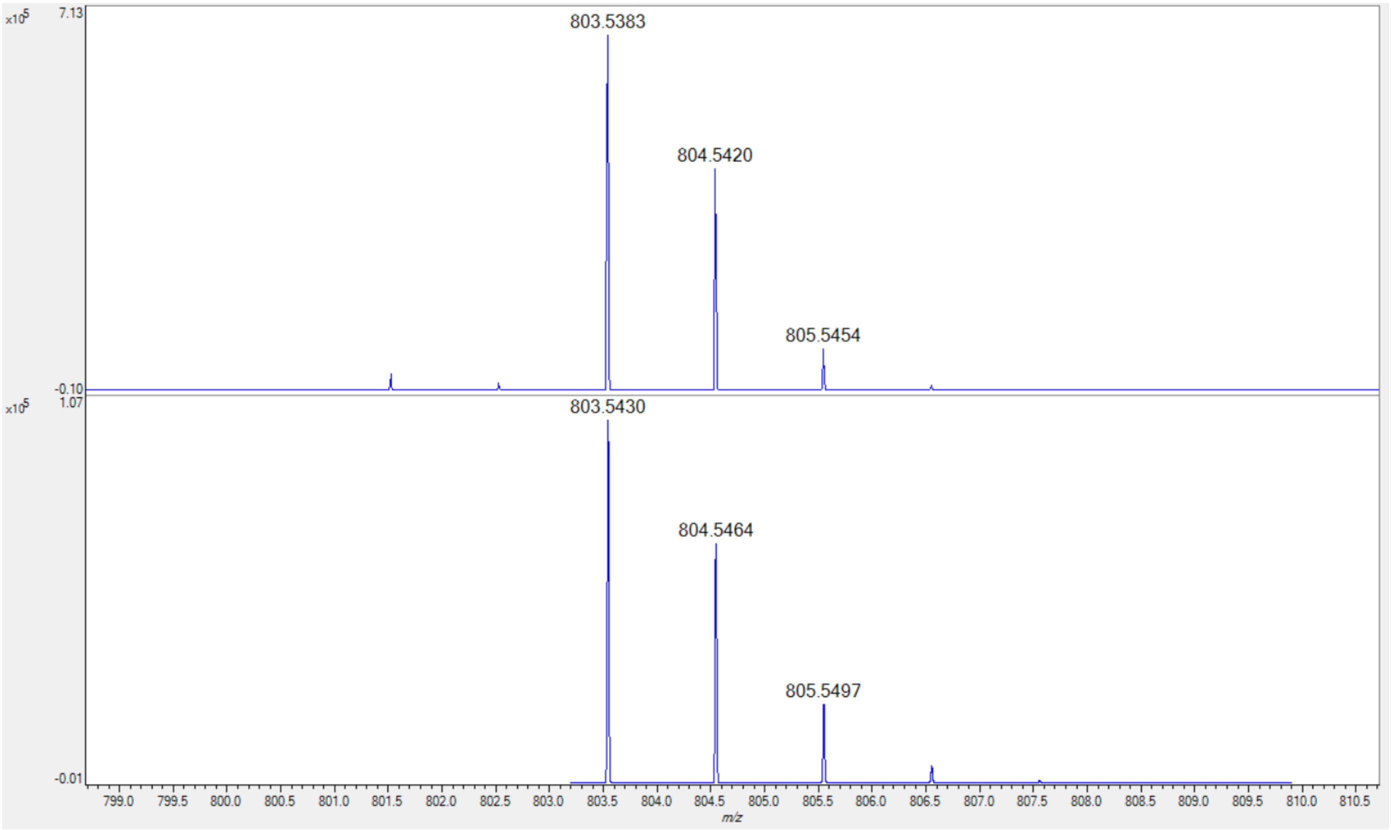

Figure S26. High resolution mass spectrum of compound **8a** (MALDI–TOF, top: experimental, bottom: simulated).

7-Butyl-5,9-bis(2,4,6-triisopropylphenyl)-7*H*-diindeno[2,1-*b*:1',2'-*h*]carbazole (**2a**)

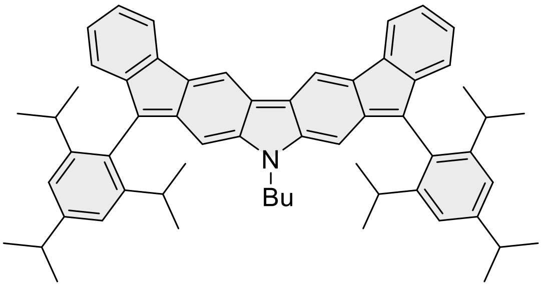

Compound **8a** (40 mg, 0.05 mmol) was dissolved in dry tetrahydrofuran (15 mL) in a flame-dried Schlenk flask and purged with nitrogen for 15 minutes. Potassium *tert*-butoxide solution (2 mol/L in 2-methyltetrahydrofuran, 150 μL, 0.3 mmol) was added slowly to the vigorously stirred solution at room temperature. The mixture was stirred for 5 minutes, and a solid copper (39 mg, 0.2 mmol) was added under nitrogen. The dark-red mixture was stirred at room temperature for 15 minutes. The mixture was passed through a celite, and the solvent was removed on a rotary evaporator. The crude mixture was purified via quick silica column chromatography using 1:4 dichloromethane/*n*-hexane as an eluent to afford compound **2a** as a red solid (29 mg, 76%).

^{1}H NMR (600 MHz, dichloromethane-d_2, 200 K): δ 7.61 (2H, s), 7.34 (2H, d, ^3J = 7.3 Hz), 7.08 (4H, s), 6.89 (2H, t, ^3J = 7.1 Hz), 6.83 (2H, t, ^3J = 7.4 Hz), 6.43 (2H, d, ^3J = 7.3 Hz), 5.81 (2H, s), 3.62 (2H, bs), 2.87 (6H, m), 1.40 (2H, m), 1.24 (12H, d, ^3J = 6.9 Hz), 1.09 (2H, m), 1.01 (24H, t, ^3J = 7.4 Hz), 0.65 (3H, t, ^3J = 7.4 Hz).

HRMS (MALDI–TOF): *m/z*: [M]$^+$ Calcd for $C_{60}H_{67}N$: 801.5268; Found 801.5268.

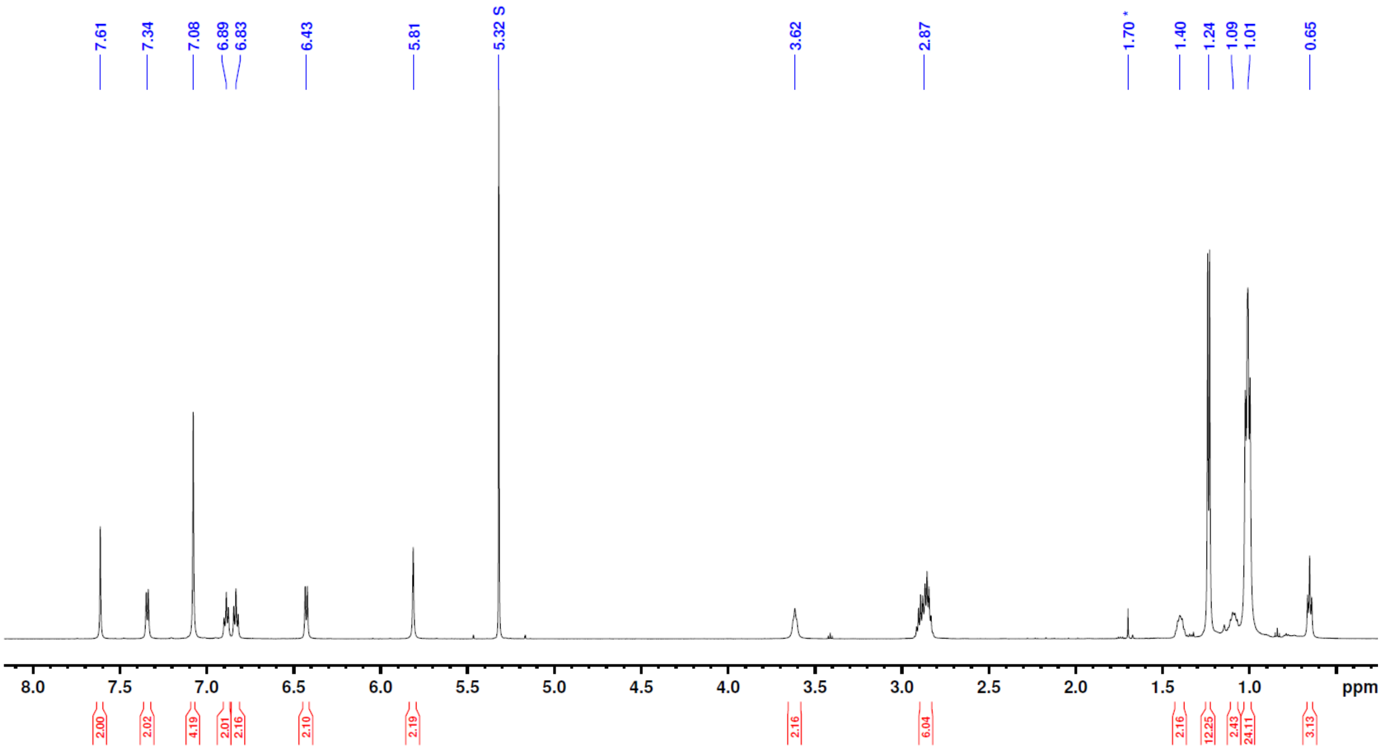

Figure S27. ^{1}H NMR spectrum of compound **2a** (600 MHz, dichloromethane-d_2, 200 K).

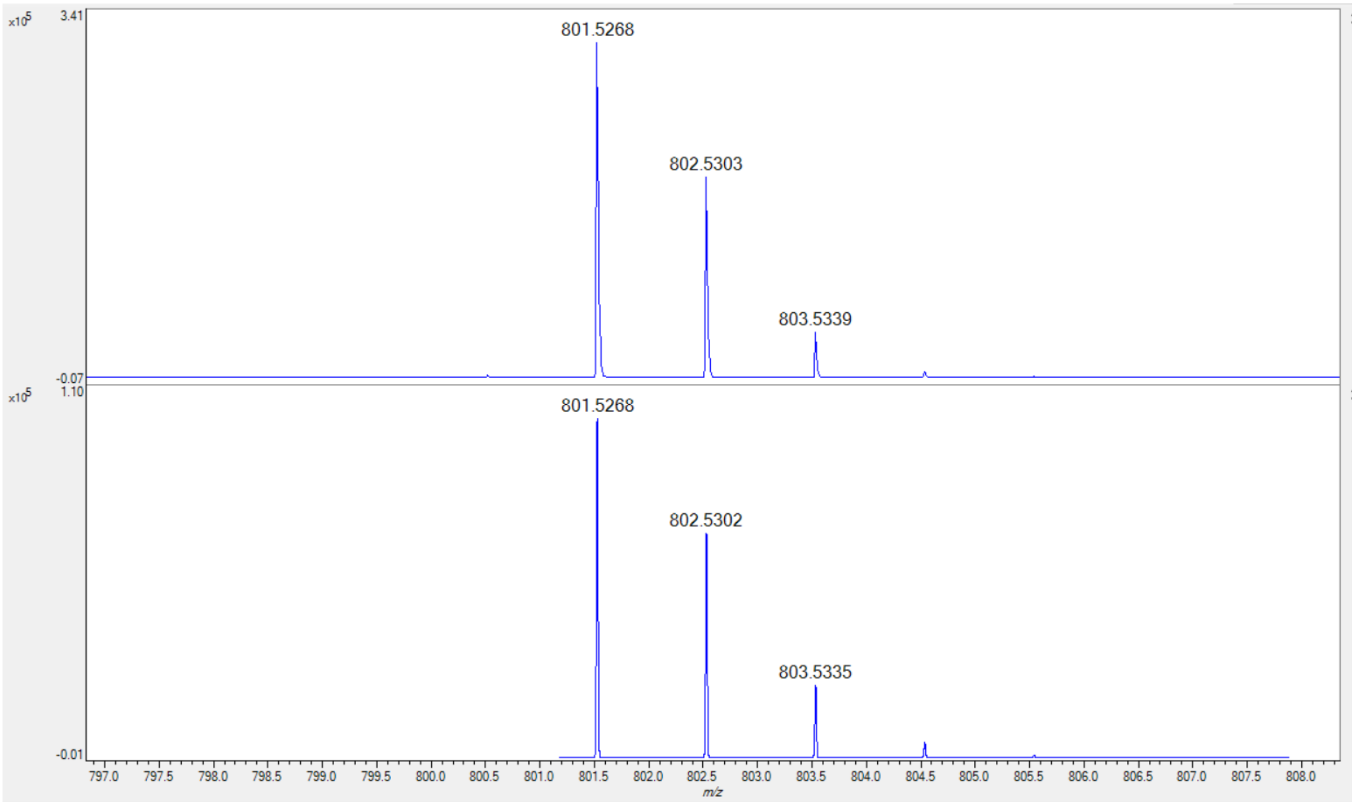

Figure S28. High resolution mass spectrum of compound **2a** (MALDI–TOF, top: experimental, bottom: simulated).

2,8-Dibromo-3,7-bis(dibromomethyl)dibenzo[*b,d*]thiophene (**S1**)

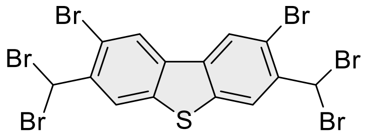

In pressure tube compound **6** (500 mg, 1.35 mmol), *N*-Bromosuccinimide (1.44 g, 8.11 mmol) and calcium carbonate (270 mg, 2.70 mmol) were dissolved in 20 mL of dichloromethane. The mixture was irradiated with blue LED light (400 nm) while stirring for 3 hours. The reaction mixture was cooled to room temperature and passed through the plug of celite. The solvent was removed on rotary evaporator. The crude mixture was purified via silica column chromatography using 20% dichloromethane in *n*-hexane as an eluent to give compound **S1** (703 mg, 76%) as a colorless solid.

^{1}H NMR (500 MHz, chloroform-*d*, 300 K): δ 8.51 (2H, s), 8.18 (2H, s), 7.17 (2H, s).

^{13}C NMR (125 MHz, chloroform-*d*, 300 K): δ 140.70, 139.73, 136.01, 125.79, 125.50, 116.45, 39.30.

HRMS (APCI–TOF): *m*/*z*: [M – Br]$^{+}$ Calcd for $C_{14}H_6Br_6S$: 606.6040; Found 606.6067.

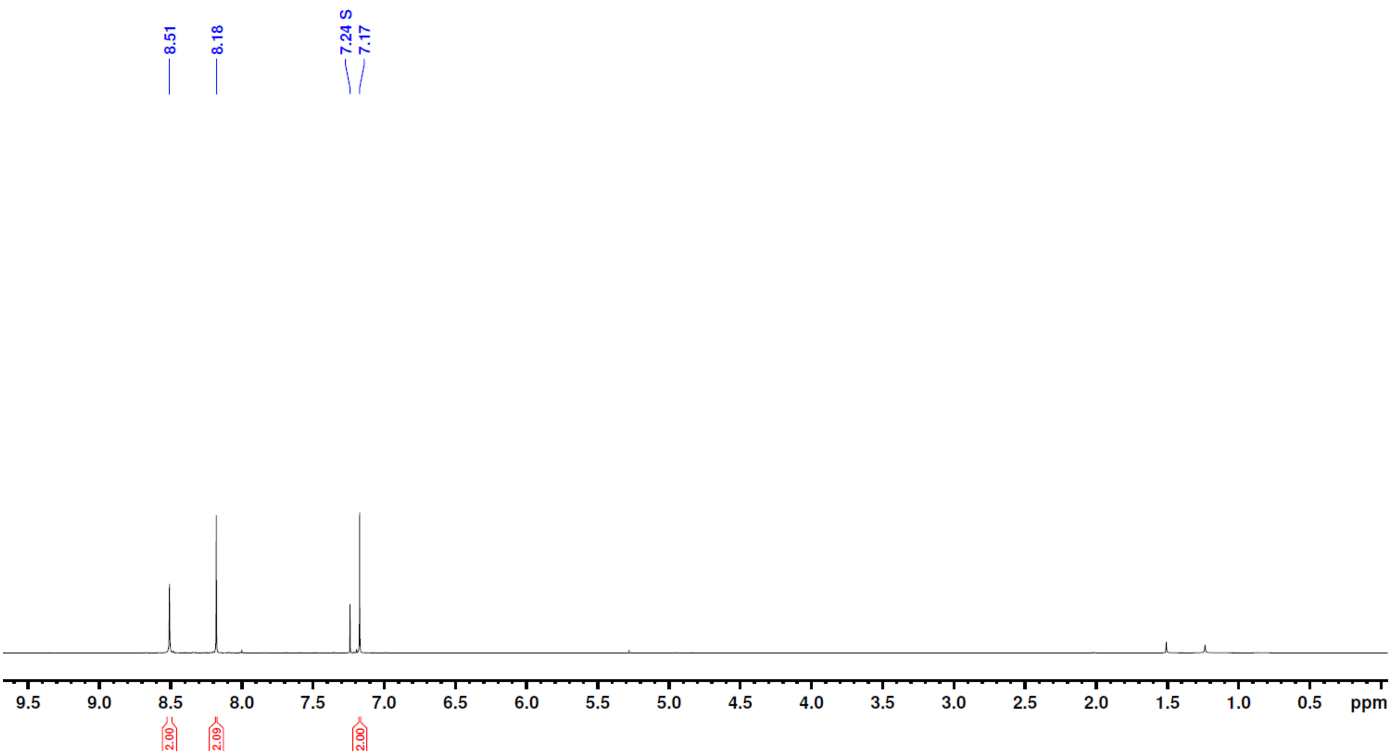

Figure S29. ^{1}H NMR spectrum of compound **S1** (500 MHz, chloroform-*d*, 300 K).

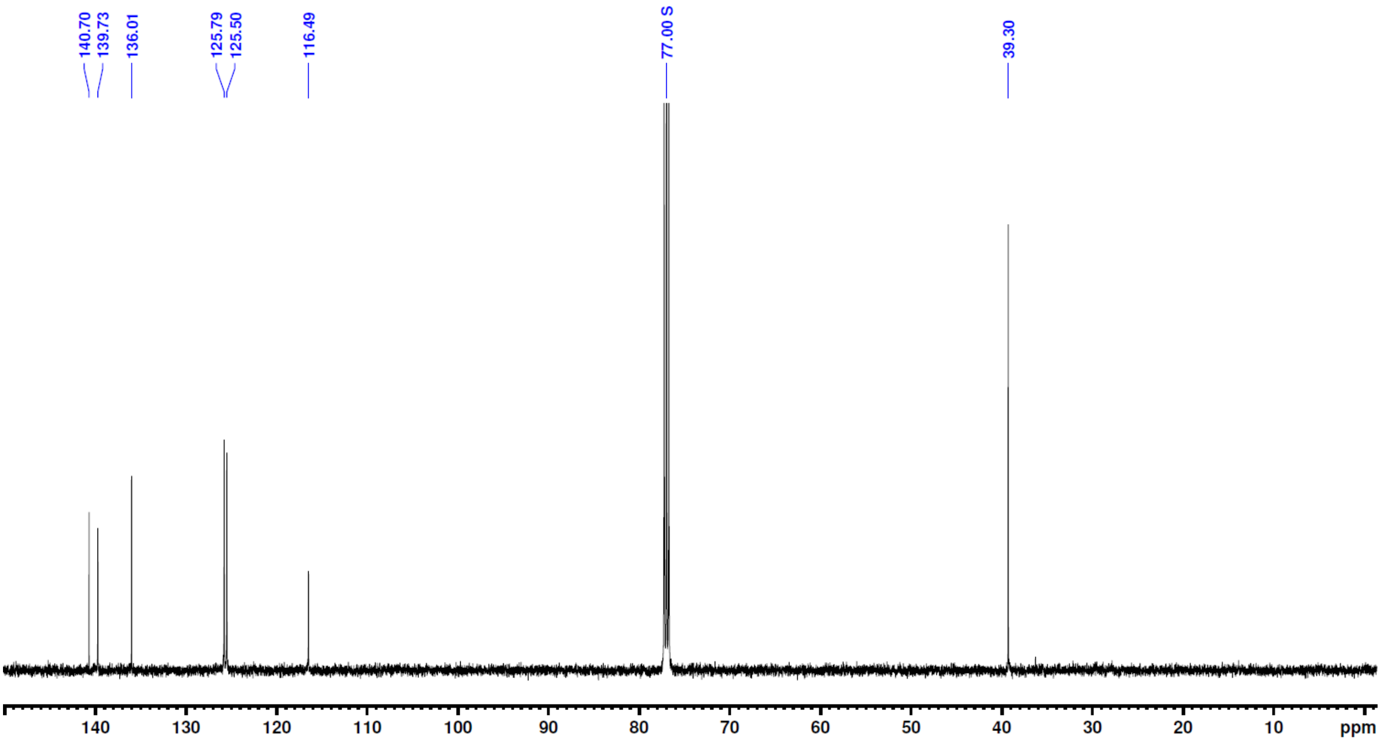

Figure S30. ^{13}C NMR spectrum of compound **S1** (125 MHz, chloroform-*d*, 300 K).

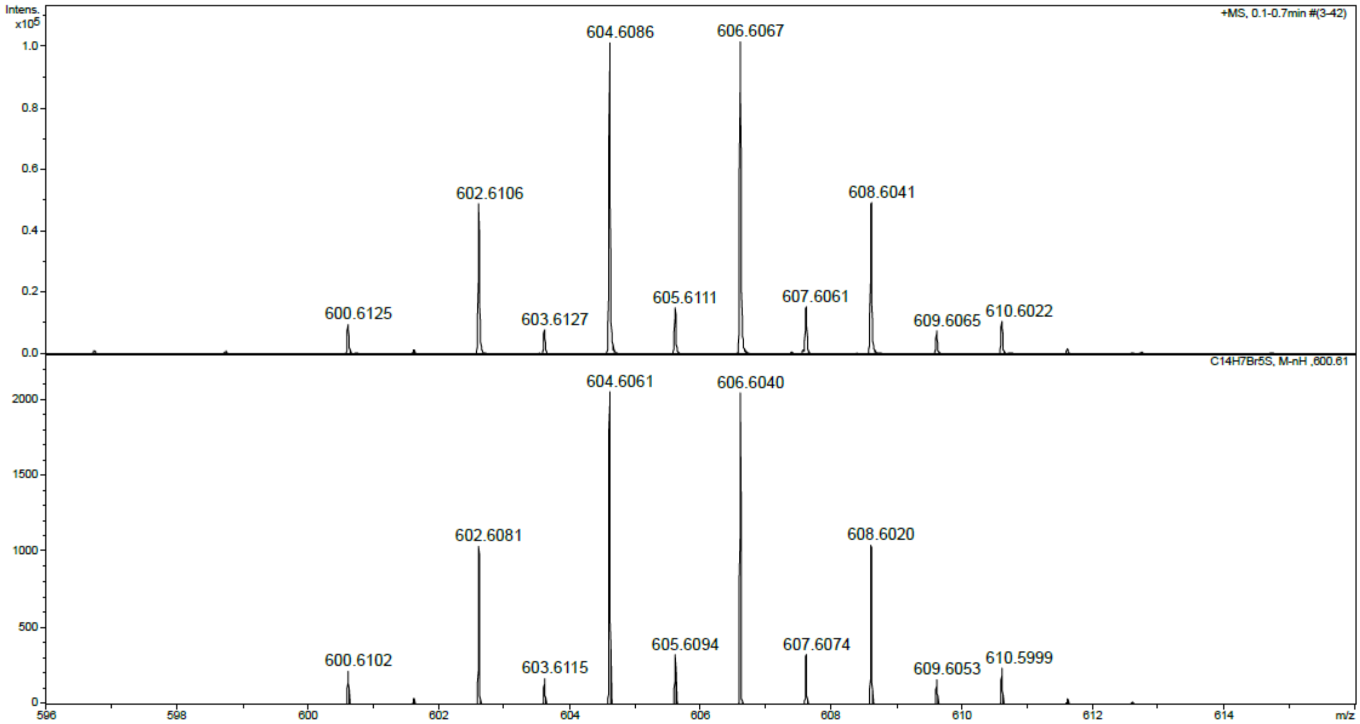

Figure S31. High resolution mass spectrum of compound **S1** (APCI–TOF, top: experimental, bottom: simulated).

2,8-Dibromodibenzo[*b,d*]thiophene-3,7-dicarbaldehyde (**6b**)

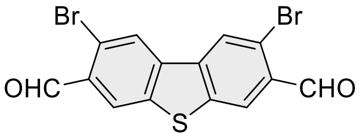

Compound **S1** (700 mg, 1.02 mmol) and $CaCO_3$ (816.70 mg, 8.17 mmol) were suspended in 3:1 *N,N*-dimethylformamide /water (28 mL) in a glass pressure tube. The tube was sealed, and the mixture was stirred at 110 °C for 12 hours. After cooling to room temperature, the reaction mixture was diluted with water and extracted with ethyl acetate. The organic phase was dried over anhydrous sodium sulfate(VI) and the solvent was removed on a rotary evaporator. The crude mixture was purified via silica column chromatography using 20% ethyl acetate in *n*-hexane as an eluent to give compound **6b** (346 mg, 85%) as a yellow solid.

^{1}H NMR (500 MHz, chloroform-*d*, 300 K): δ 10.49(2H, s), 8.43(2H, s), 8.43(2H, s).

^{13}C NMR (125 MHz, chloroform-*d*, 300 K): δ 190.95, 141.19, 138.94, 132.52, 127.73, 124.84, 122.39.

HRMS (APCI–TOF): *m*/*z*: $[M + H]^+$ Calcd for $C_{14}H_6Br_2O_2S$: 398.8508; Found 398.8534.

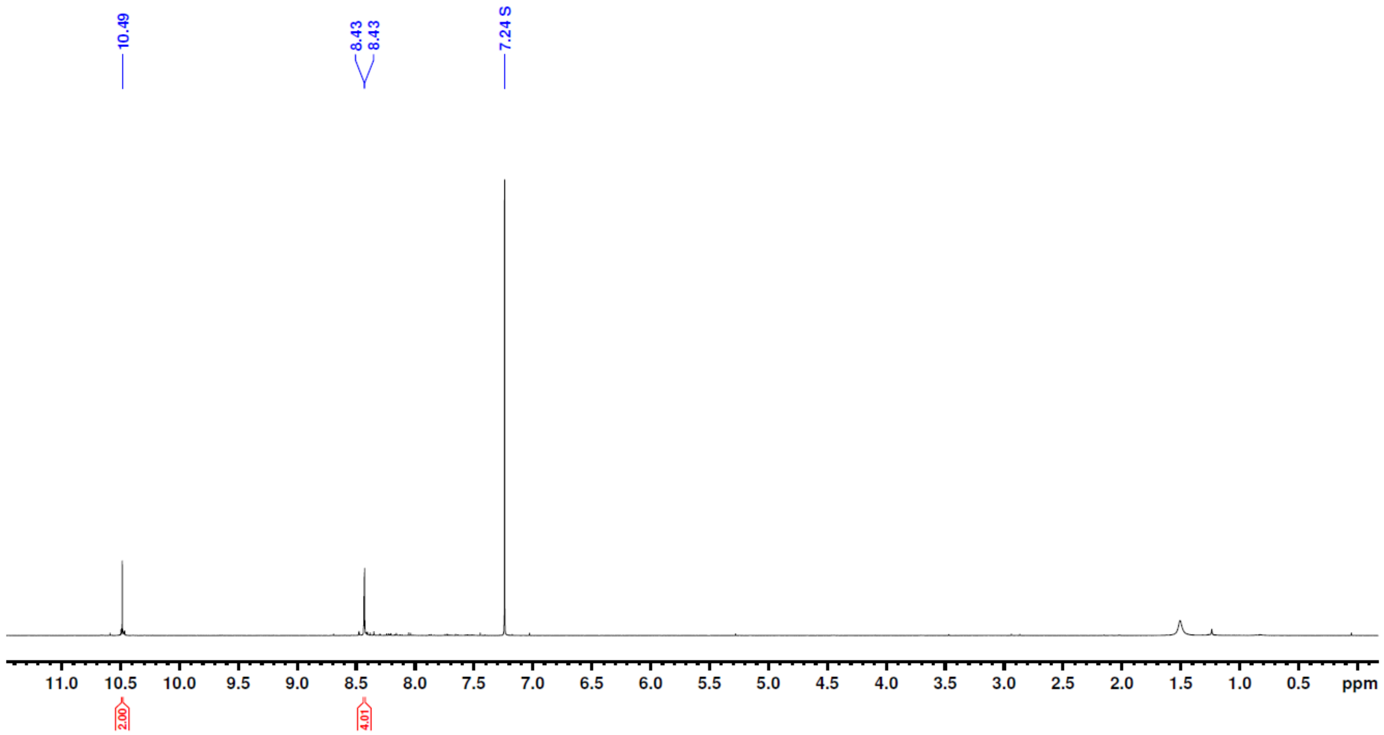

Figure S32. ^{1}H NMR spectrum of compound **6b** (500 MHz, chloroform-*d*, 300 K).

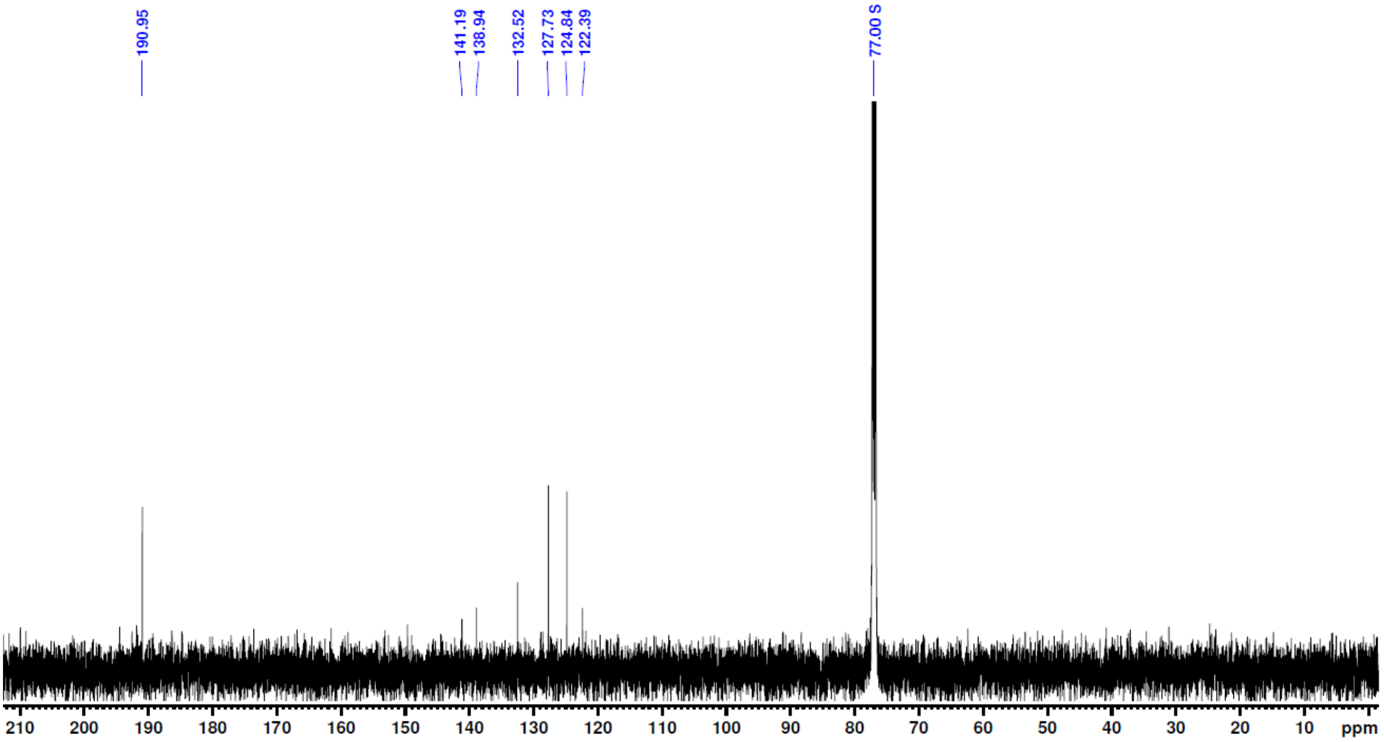

Figure S33. ^{13}C NMR spectrum of compound **6b** (125 MHz, chloroform-*d*, 300 K).

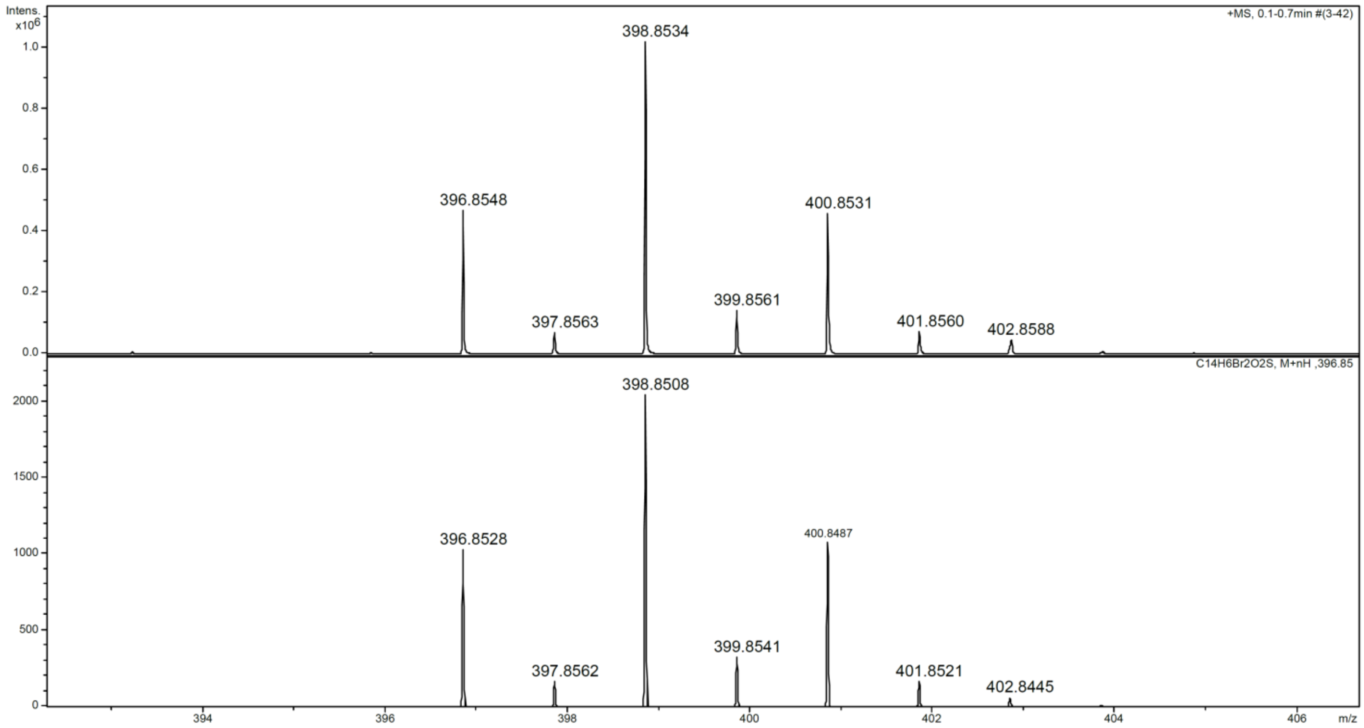

Figure S34. High resolution mass spectrum of compound **6b** (APCI–TOF, top: experimental, bottom: simulated).

2,8-Diphenyldibenzo[*b,d*]thiophene-3,7-dicarbaldehyde (**7b**)

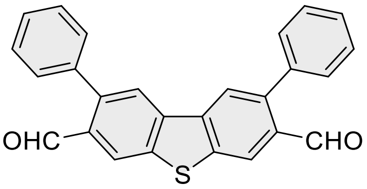

Compound **6b** (200 mg, 0.5 mmol) and phenyl boronic acid (161.21 mg, 1.26 mmol) was dissolved in 50 mL of dioxane and the solution was purged with nitrogen for 15 minutes. The degassed solution of sodium carbonate

(426 mg, 4.02 mmol) in 5 mL of water was added to the mixture followed by the addition of tetrakis (triphenylphosphinepalladium(0) (58 mg, 0.05 mmol) The mixture was purged with nitrogen for few minutes and heated at 100 °C for 24 hours. After cooling to room temperature, water was added and extracted with dichloromethane. The organic layer was washed with brine, dried over anhydrous sodium sulfate(VI) and the solvent was removed on rotary evaporator. The crude mixture was purified via silica column chromatography using 50% dichloromethane in *n*-hexane as an eluent to give compound **7b** (161 mg, 82%) as a yellow solid.
^{1}H NMR (500 MHz, chloroform-*d*, 300 K): δ 10.08 (2H, s), 8.57 (2H, s), 8.25 (2H, s), 7.48 (10H, m).
^{13}C NMR (125 MHz, chloroform-*d*, 300 K): δ 191.52, 142.30, 140.93, 138.47, 137.55, 130.28, 128.62, 124.65, 122.80 .
HRMS (APCI–TOF): *m/z*: [M + H]$^{+}$ Calcd for $C_{26}H_{16}O_{2}S$: 393.0944; Found 393.0961.

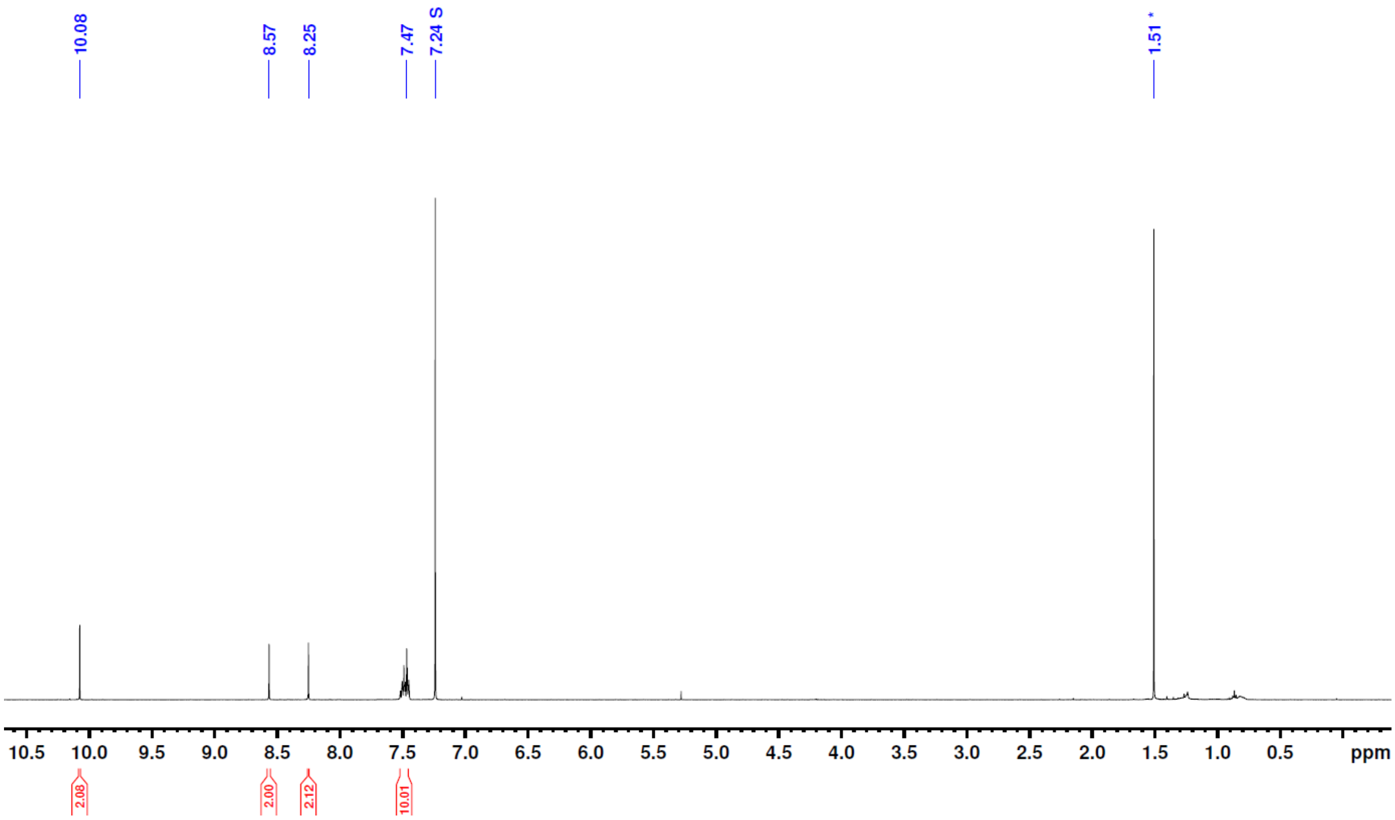

Figure S35. ^{1}H NMR spectrum of compound **7b** (500 MHz, chloroform-*d*, 300 K).

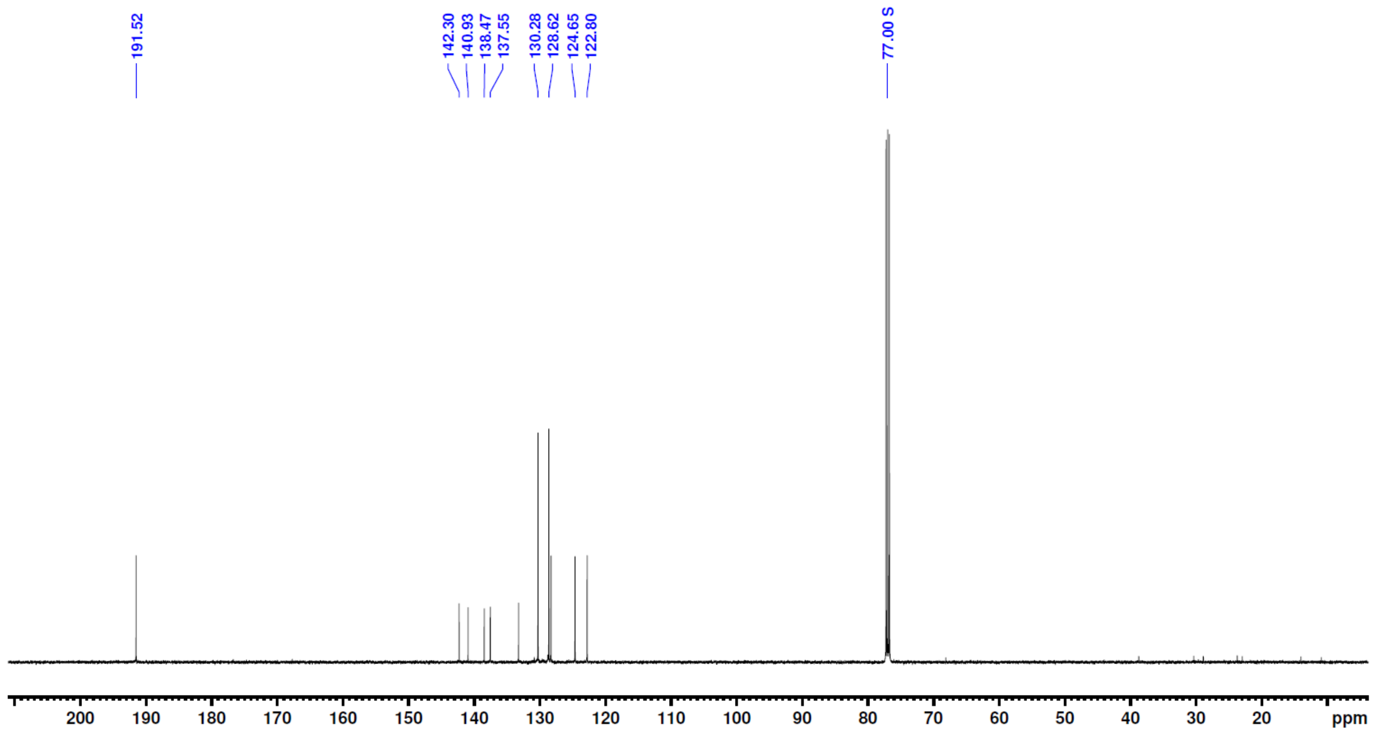

Figure S36. ^{13}C NMR spectrum of compound **7b** (125 MHz, chloroform-*d*, 300 K).

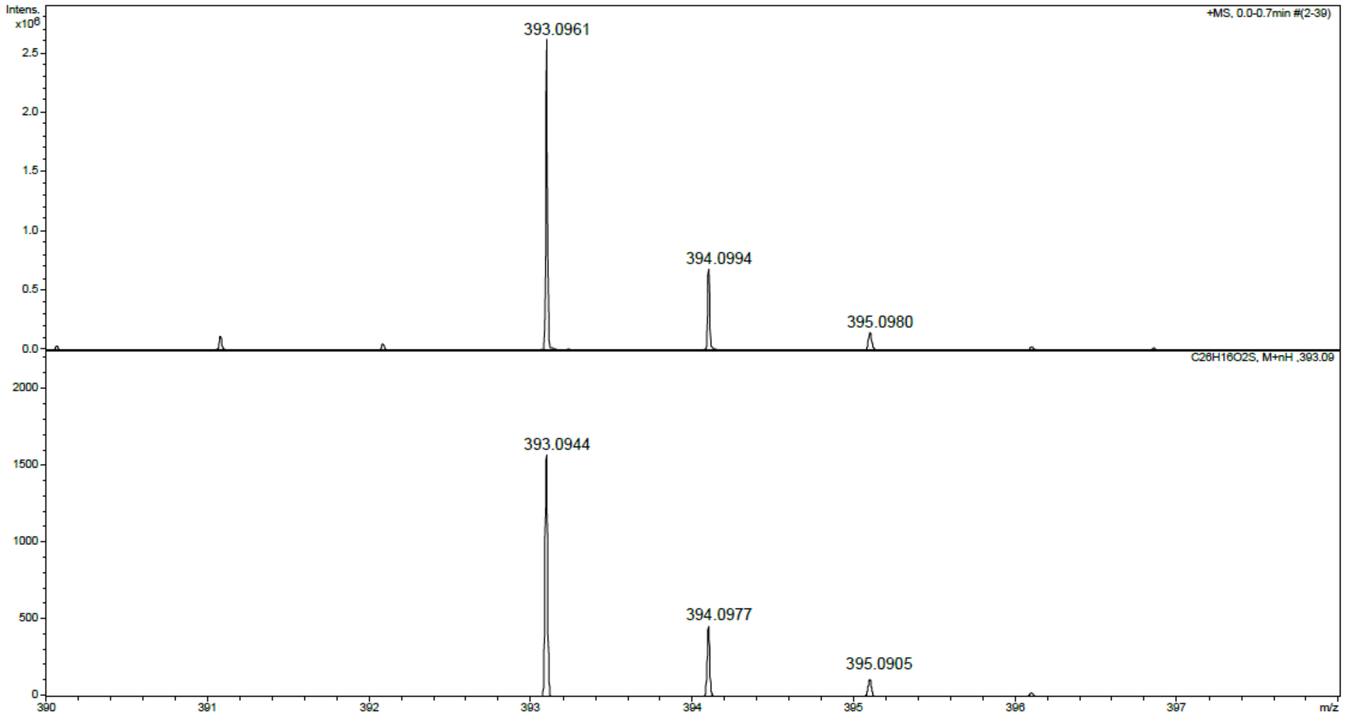

Figure S37. High resolution mass spectrum of compound **7b** (APCI–TOF, top: experimental, bottom: simulated).

5,9-Bis(2,4,6-triisopropylphenyl)-5,9-dihydrodifluoreno[2,3-*b*:3',2'-*d*]thiophene (**8b**)

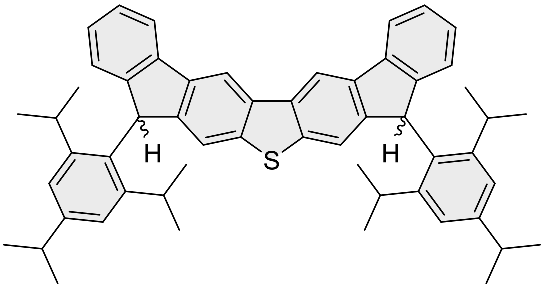

Compound **7b** (50 mg, 0.13 mmol) was dissolved in dry tetrahydrofuran (15 mL) in a flame-dried Schlenk flask and purged with nitrogen for 15 minutes. TIPMgBr (1.6 mL, 0.5 M in tetrahydrofuran, 0.8 mmol) was added dropwise at room temperature and stirred overnight under nitrogen. The mixture was quenched with water, extracted with dichloromethane, dried over anhydrous sodium sulfate(VI) and evaporated to dryness. The resulting solid was dissolved in freshly distilled dichloromethane (25 mL) and purged with nitrogen for 15 minutes. 0.2 mL of boron trifluoride diethyl etherate were added slowly and stirred for 15 minutes at room temperature. The mixture was quenched with water, treated with saturated sodium bicabonate, and extracted with dichloromethane. The organic layer was washed with brine, dried over anhydrous sodium sulfate(VI) and the solvent was removed on a rotary evaporator. The crude mixture was purified via silica column chromatography using 20% dichloromethane in *n*-hexane as an eluent to give compound **8b** as a colorless solid (81 mg, 83%, mixture of stereoisomers).

^{1}H NMR (500 MHz, chloroform-*d*, 300 K): δ 8.73 (2H, d, ^3J = 2.96 Hz), 8.05 (2H, m), 7.63 (2H, d, ^3J = 3.1 Hz), 7.45 (2H, m), 7.27 (4H, m), 7.14(2H, s), 6.86 (2H, s), 5.66 (2H, s), 3.61(2H, m), 2.89 (2H, m), 1.61 (2H, m), 1.51 (1H, s), 1.45 (12H, m), 1.27 (12H, d, ^3J = 6.91 Hz), 1.25 (1H, s), 0.42 (9H, m), 0.37 (3H, d, ^3J = 6.84 Hz).

^{13}C NMR (125 MHz, chloroform-*d*, 300 K): δ 149.44, 149.40, 149.09, 149.08, 148.82, 148.72, 147.96, 147.94, 147.53, 147.52, 139.93, 139.91, 138.92, 138.86, 137.74, 137.66, 134.56, 134.51, 131.11, 131.07, 127.12, 127.08, 126.78, 124.37, 123.00, 120.81, 120.16, 120.07, 118.29, 118.24, 112.74, 112.71, 48.22, 48.19, 34.02, 30.99, 30.95, 29.70, 28.70, 24.86, 24.81, 24.72, 23.99, 23.95, 23.67, 23.42, 23.37, 23.35.

HRMS (MALDI–TOF): *m/z*: [M]$^+$ Calcd for $C_{56}H_{60}S$: 764.4416; Found 764.3237.

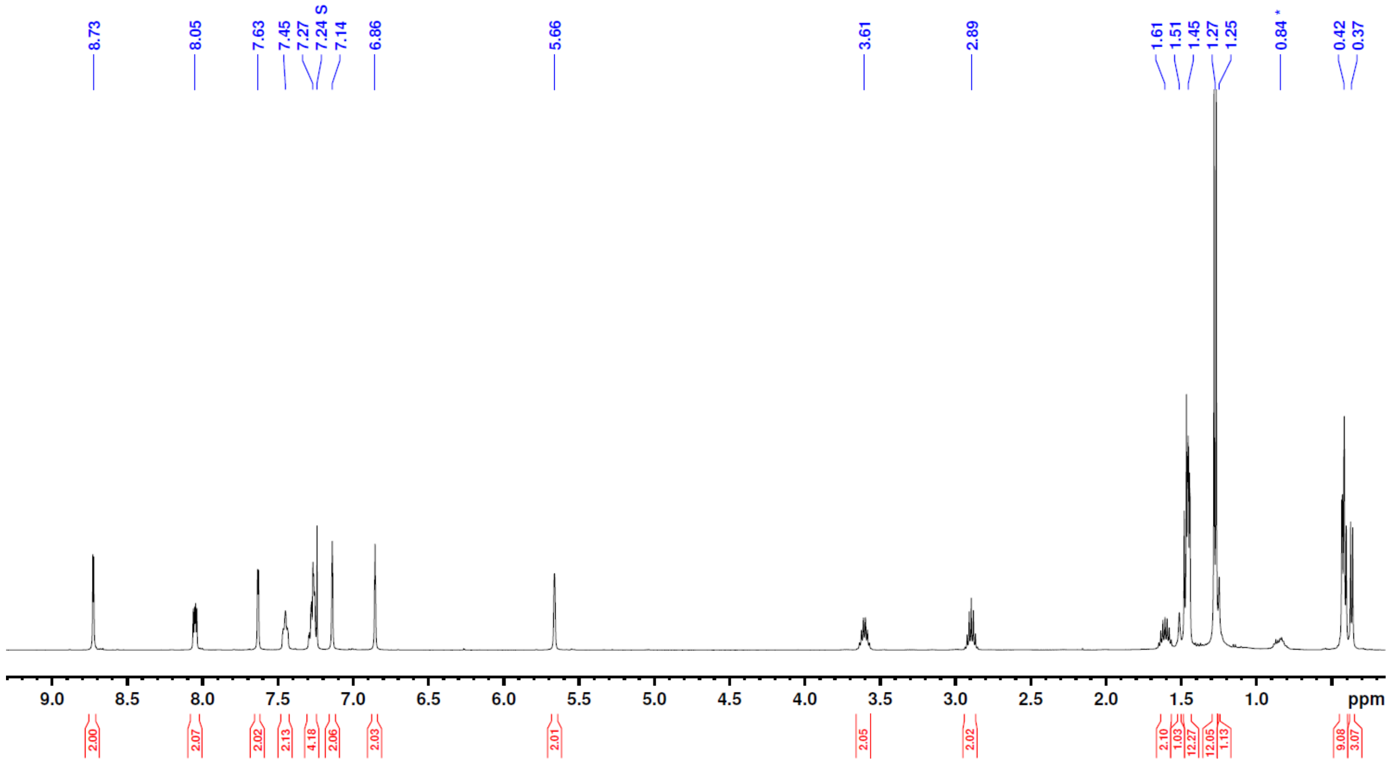

Figure S38. ^{1}H NMR spectrum of compound **8b** (500 MHz, chloroform-*d*, 300 K).

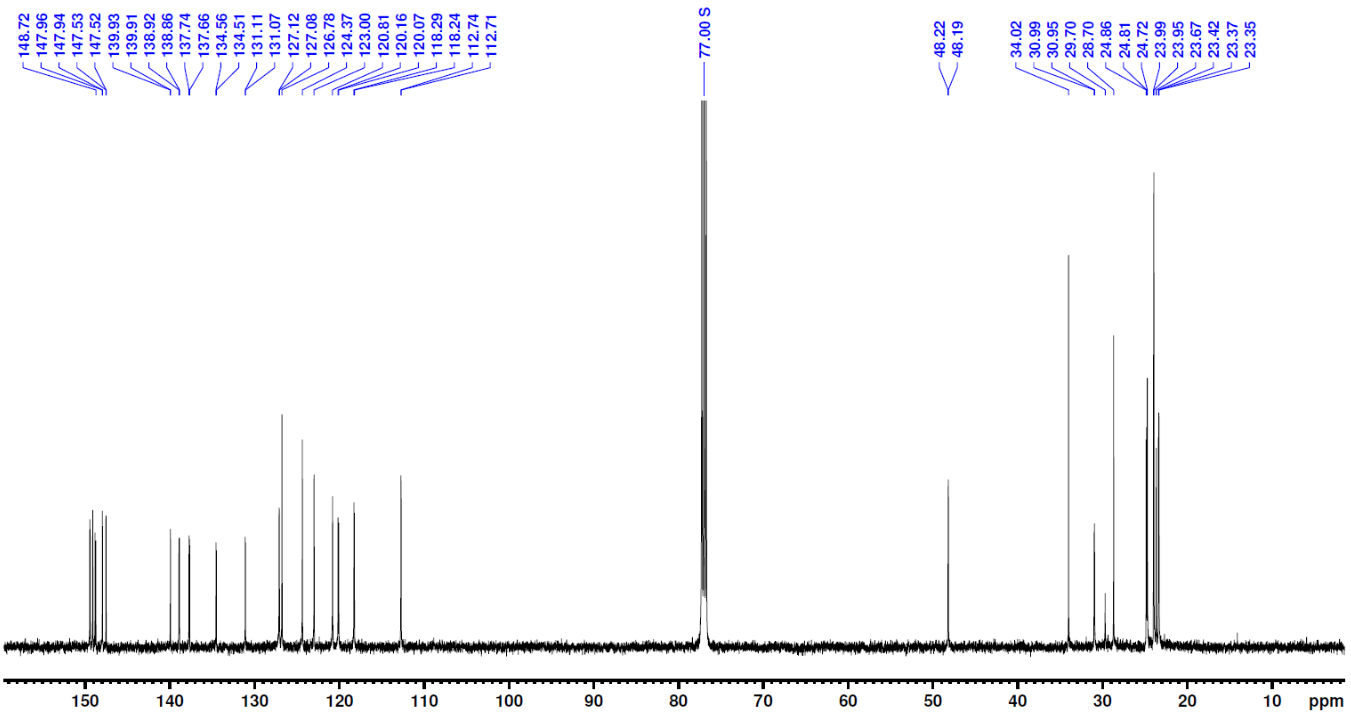

Figure S39. ^{13}C NMR spectrum of compound **8b** (125 MHz, chloroform-*d*, 300 K).

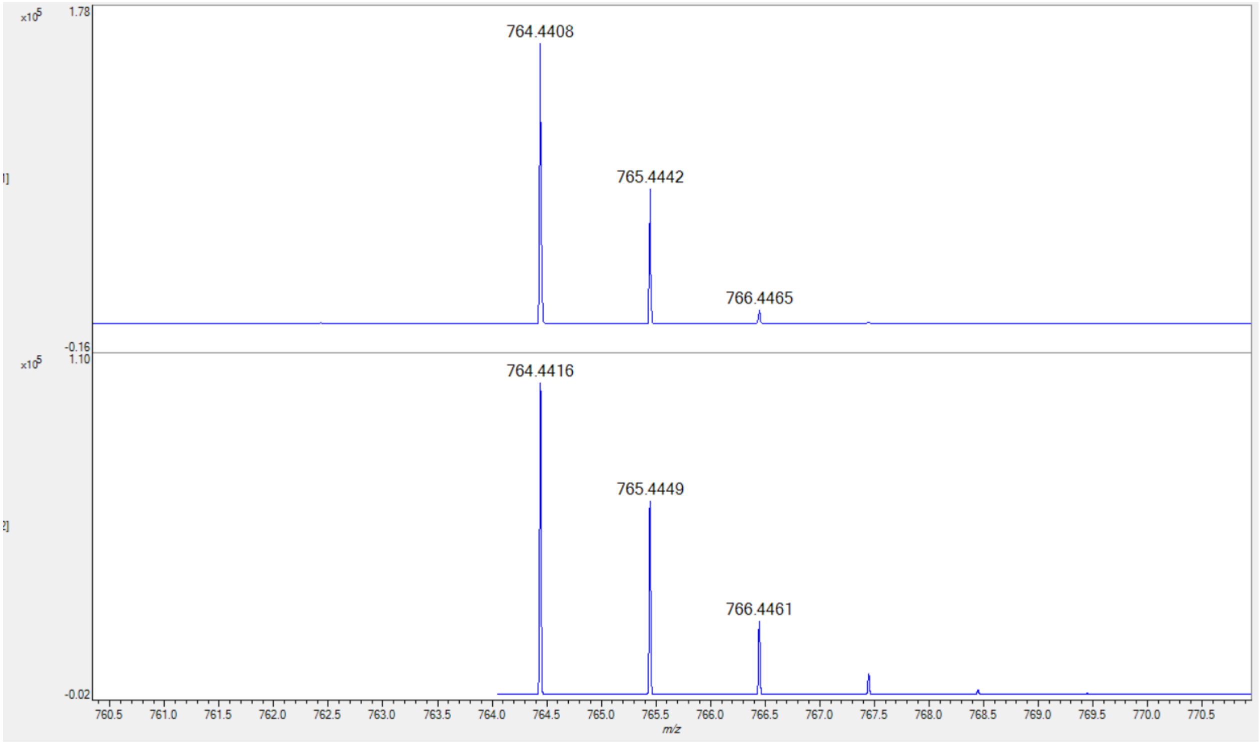

Figure S40. High resolution mass spectrum of compound **8b** (MALDI–TOF, top: experimental, bottom: simulated).

5,9-Bis(2,4,6-triisopropylphenyl)difluoreno[2,3-*b*:3',2'-*d*]thiophene (**2b**)

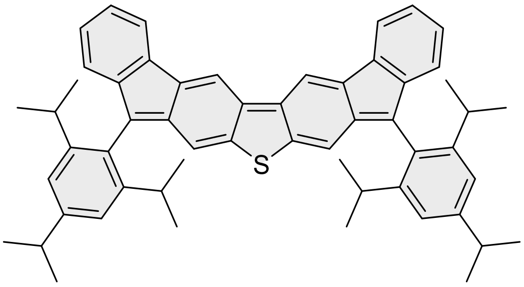

Compound **8b** (50 mg, 65 μmol) was dissolved in dry tetrahydrofuran (20 mL) in a flame-dried Schlenk flask and purged with nitrogen for 15 minutes. Potassium *tert*-butoxide solution (2 mol/L in 2-methyltetrahydrofuran, 200 μL, 0.39 mmol) was added slowly to the vigorously stirred solution at room temperature. The mixture was stirred for 5 minutes, and solid copper iodide (51 mg, 0.26 mmol) was added to the mixture under nitrogen. The dark-red mixture was stirred at room temperature for 15 minutes. The mixture was passed through a celite, and the solvent was removed on a rotary evaporator. The crude mixture was purified via quick silica column chromatography using 1:5 dichloromethane/*n*-hexane as an eluent to afford compound **2b** as a pink solid (41 mg, 82%).

^{1}H NMR (600 MHz, dichloromethane-d_2, 200 K): δ 8.06 (2H, s), 7.66 (2H, d, ^3J = 7.28 Hz), 7.12 (2H, t, ^3J = 7.15 Hz), 7.08 (4H, s), 7.05 (2H, t, ^3J = 7.45 Hz), 6.75 (2H, d, ^3J = 7.4 Hz), 6.72 (2H, s), 2.89 (2H, m), 2.72 (4H, m), 1.23 (12H, d, ^3J = 6.96 Hz), 0.99 (24H, bs).

HRMS (MALDI–TOF): *m/z*: [M]$^+$ Calcd for $C_{56}H_{58}S$: 762.4259; Found 762.4220.

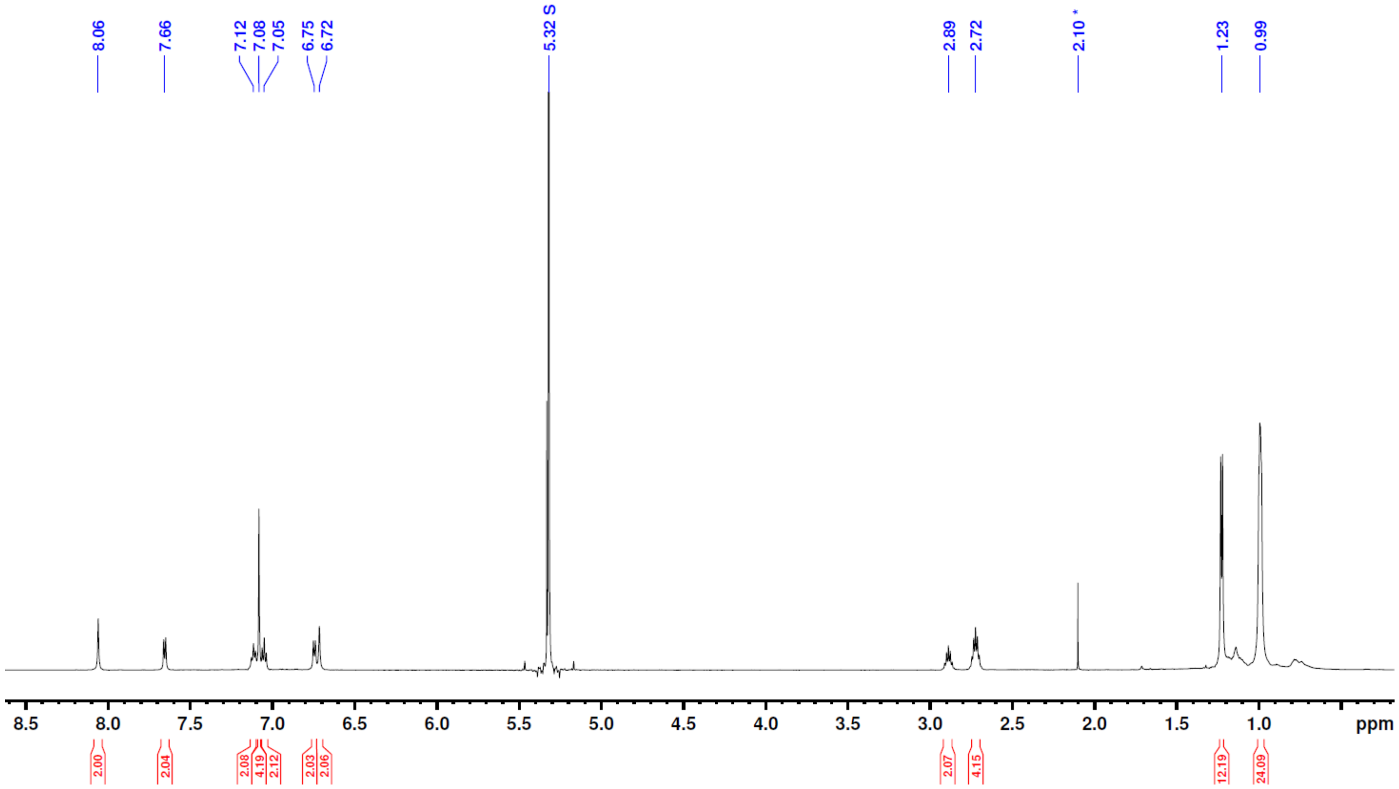

Figure S41. ^{1}H NMR spectrum of compound **2b** (600 MHz, dichloromethane-d_2, 200 K).

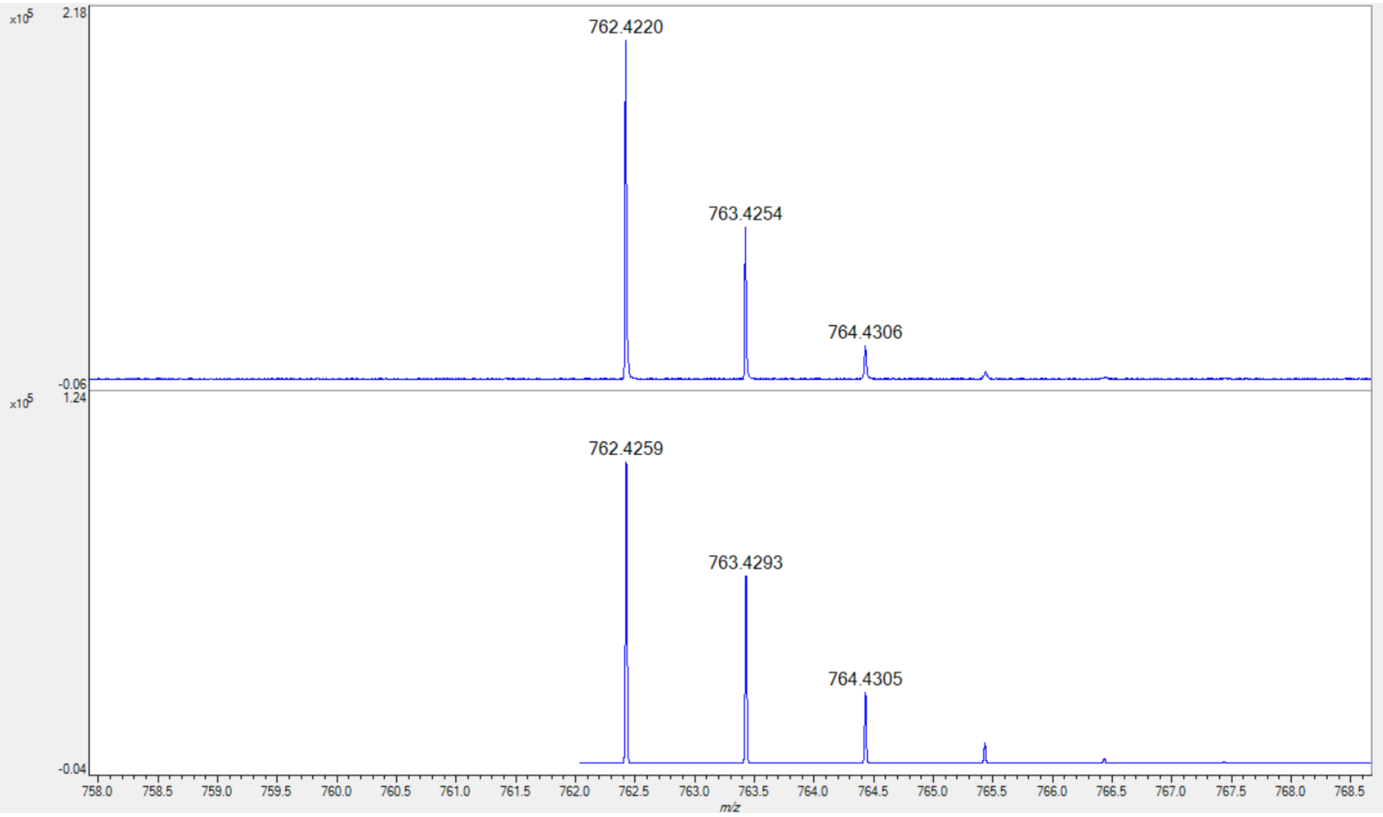

Figure S42. High resolution mass spectrum of compound **2b** (MALDI–TOF, top: experimental, bottom: simulated).

3. Aditional Schemes

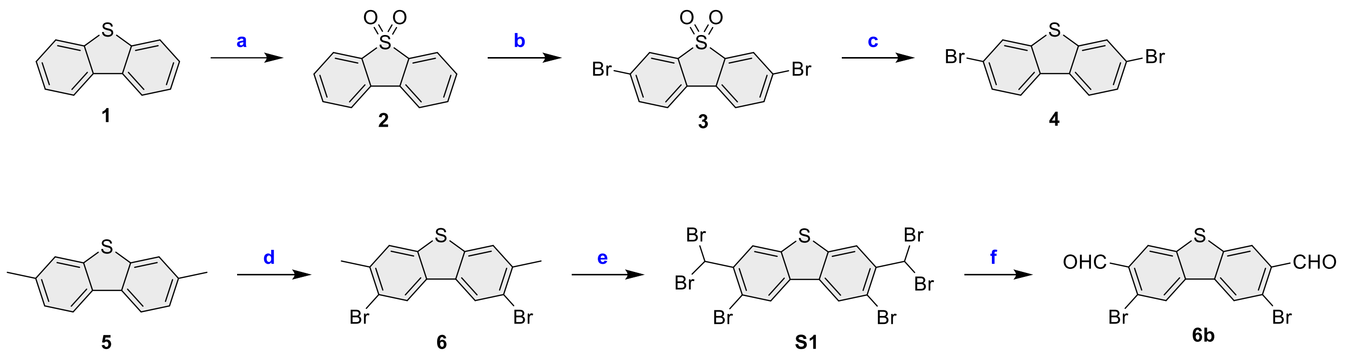

Scheme S1. Synthesis of compound **6b**. Reagents and conditions: a) H_2O_2 (5.0 equiv.), AcOH; b) NBS (3.0 equiv.), H_2SO_4; c) $LiAlH_4$ (5.0 equiv.), Et_2O; d) Br_2 (2.2 equiv.), $CHCl_3$; e) NBS (6.0 equiv.), $CaCO_3$ (2equiv.), DCM; f) CaCO3 (8.0 equiv.), DMF/H_2O 3:1;.

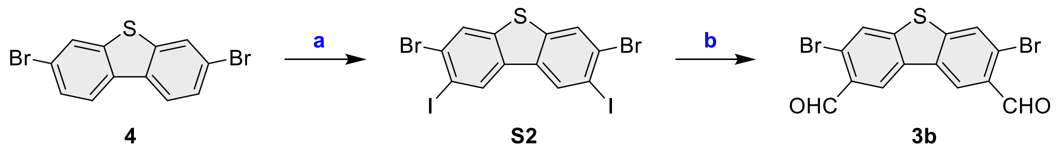

Scheme S2. Synthesis of compound **3b**. Reagents and conditions: a)Br_2 (5.0 equiv.), Iron (0.05 equiv.), $CHCl_3$/H_2SO_4; b) 1. *i*-PrMgCl (2.2 equiv.), THF, 2. DMF (10 equiv.); .

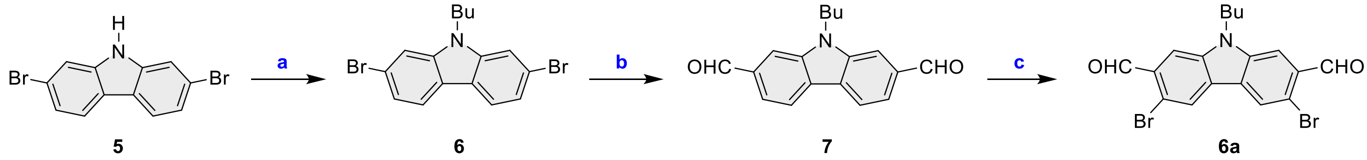

Scheme S3. Synthesis of compound **6a**. Reagents and conditions: a) C_4H_9Br (1.2 equiv.), NaOH, NBu_4ClO_4, 2-butanone; b) *n*- BuLi (4 equiv.), DMF (6 equiv.), THF; c) Br_2 (2.2 equiv.), CH_2Cl_2; .

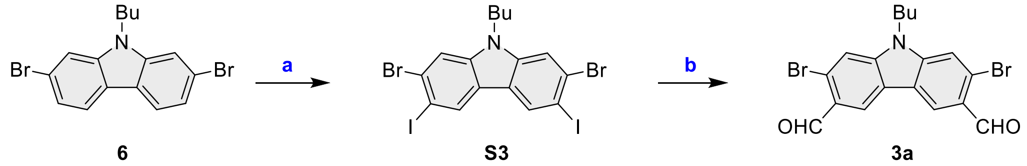

Scheme S4. Synthesis of compound **3a**. Reagents and conditions: a)KI (2.5 equiv.), KIO_3 (1.5 equiv.), AcOH; b) 1. *i*-PrMgCl (2.2 equiv.), THF, 2. DMF (10 equiv.); .

4. Experimental Data

4.1. X-ray Crystallography

Table S1. Crystal data and structure refinement for **2b**·1.5C_7H_8. .

Identification code	tw19ca
Empirical formula	$C_{66.5}H_{70}S$
Formula weight	913.35
Temperature/K	100(2)
Crystal system	triclinic
Space group	P-1
a/Å	9.2015(19)
b/Å	15.281(4)
c/Å	20.007(3)
α/°	104.66(2)
β/°	97.26(2)
γ/°	102.40(2)
Volume/Å^3	2609.0(10)
Z	2
ρ_{calc}g/cm^3	1.163
μ/mm^{-1}	0.843
F(000)	970.0
Crystal size/mm^3	0.390 × 0.040 × 0.020
Radiation	Cu Kα (λ = 1.54184)
2θ range for data collection/°	4.652 to 147.966
Index ranges	$-11 \leq h \leq 7$, $-18 \leq k \leq 18$, $-24 \leq l \leq 24$
Reflections collected	39794
Independent reflections	10083 [R_{int} = 0.0552, R_{sigma} = 0.0388]
Data/restraints/parameters	10083/3/665
Goodness-of-fit on F^2	1.087
Final R indexes [I>=2σ (I)]	R_1 = 0.0575, wR_2 = 0.1654
Final R indexes [all data]	R_1 = 0.0666, wR_2 = 0.1725
Largest diff. peak/hole / e Å^{-3}	0.60/-0.55

.

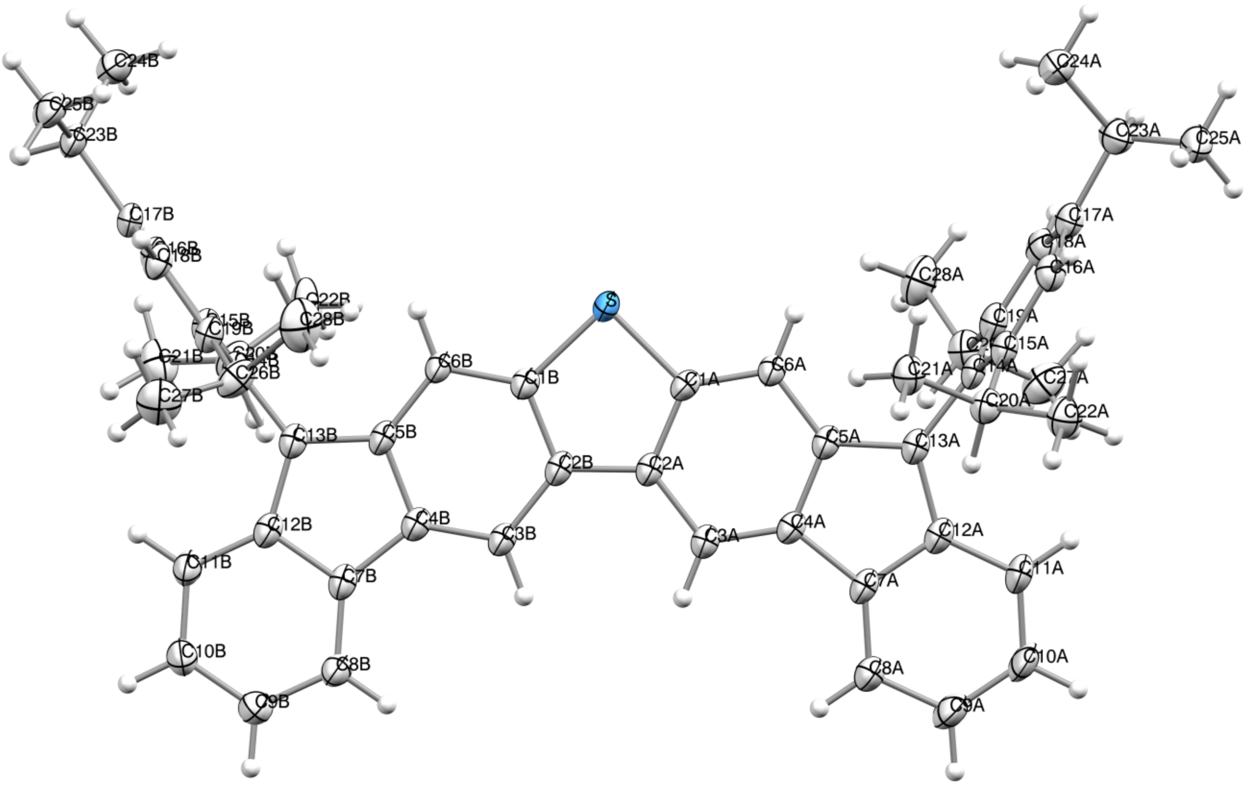

Figure S43. ORTEP plot for **2b**·$1.5C_7H_8$.Thermal ellipsoids are at the 50% probability level. Solvent molecules and disorder positions are removed for clarity.

Table S2. Crystal data and structure refinement for **2a**. .

Identification code	tw044a
Empirical formula	$C_{60}H_{67}N$
Formula weight	802.14
Temperature/K	100(2)
Crystal system	triclinic
Space group	P-1
a/Å	9.141(2)
b/Å	16.305(4)
c/Å	16.996(5)
α/°	105.22(4)
β/°	91.86(4)
γ/°	99.67(3)
Volume/Å^3	2401.8(12)
Z	2
ρ_{calc}g/cm^3	1.109
μ/mm^{-1}	0.467
F(000)	868.0
Crystal size/mm^3	0.4 × 0.03 × 0.013
Radiation	Cu Kα (λ = 1.54184)
2θ range for data collection/°	5.406 to 148.824
Index ranges	$-11 \leq h \leq 11, -20 \leq k \leq 18, -20 \leq l \leq 19$
Reflections collected	31595
Independent reflections	9269 [R_{int} = 0.0560, R_{sigma} = 0.0713]
Data/restraints/parameters	9269/0/567
Goodness-of-fit on F^2	1.031
Final R indexes [I>=2σ (I)]	R_1 = 0.1176, wR_2 = 0.3186
Final R indexes [all data]	R_1 = 0.1595, wR_2 = 0.3408
Largest diff. peak/hole / e Å^{-3}	0.44/-0.49

.

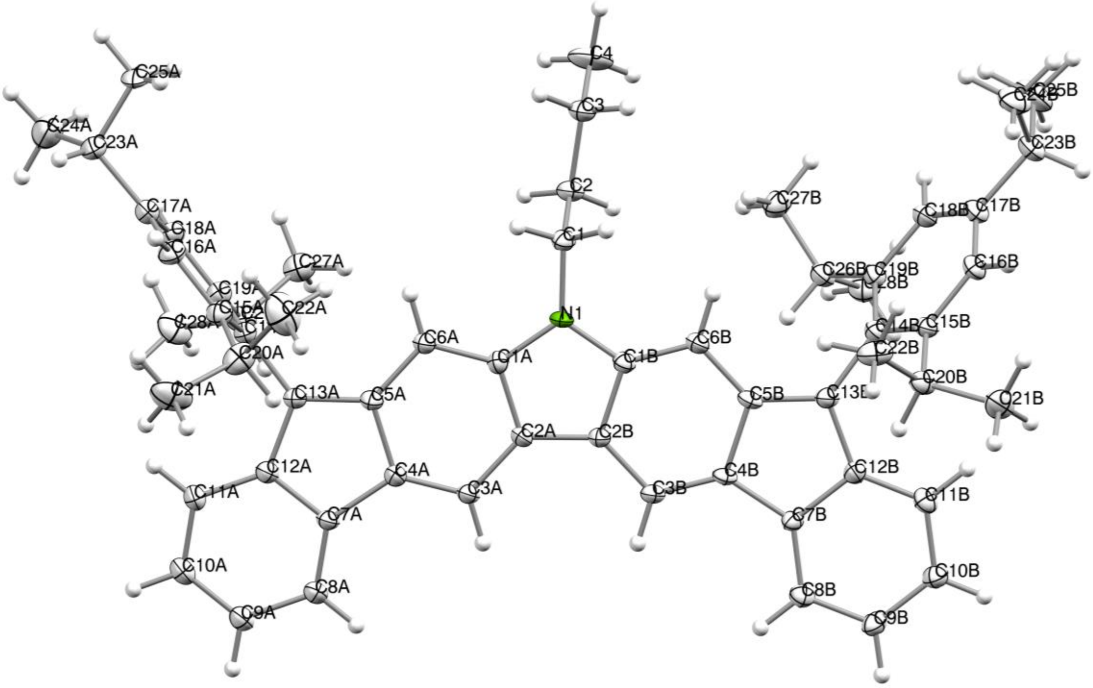

Figure S44. ORTEP plot for **2a**.Thermal ellipsoids are at the 30% probability level.

Table S3. Crystal data and structure refinement for **1a**. .

Identification code	tw043a
Empirical formula	$C_{60}H_{67}N$
Formula weight	802.207
Temperature/K	100.15
Crystal system	monoclinic
Space group	$P2_1/n$
a/Å	9.742(2)
b/Å	15.560(4)
c/Å	30.552(12)
α/°	90
β/°	93.27(4)
γ/°	90
Volume/Å^3	4624(2)
Z	4
ρ_{calc}g/cm^3	1.152
μ/mm^{-1}	0.485
F(000)	1740.7
Crystal size/mm^3	0.19 × 0.03 × 0.02
Radiation	Cu Kα (λ = 1.54184)
2θ range for data collection/°	5.8 to 148.96
Index ranges	$-9 \leq h \leq 12$, $-19 \leq k \leq 18$, $-37 \leq l \leq 37$
Reflections collected	32984
Independent reflections	9022 [R_{int} = 0.0940, R_{sigma} = 0.0914]
Data/restraints/parameters	9022/0/563
Goodness-of-fit on F^2	1.020
Final R indexes [I>=2σ (I)]	R_1 = 0.0633, wR_2 = 0.1556
Final R indexes [all data]	R_1 = 0.1197, wR_2 = 0.1911
Largest diff. peak/hole / e Å^{-3}	0.50/-0.48

.

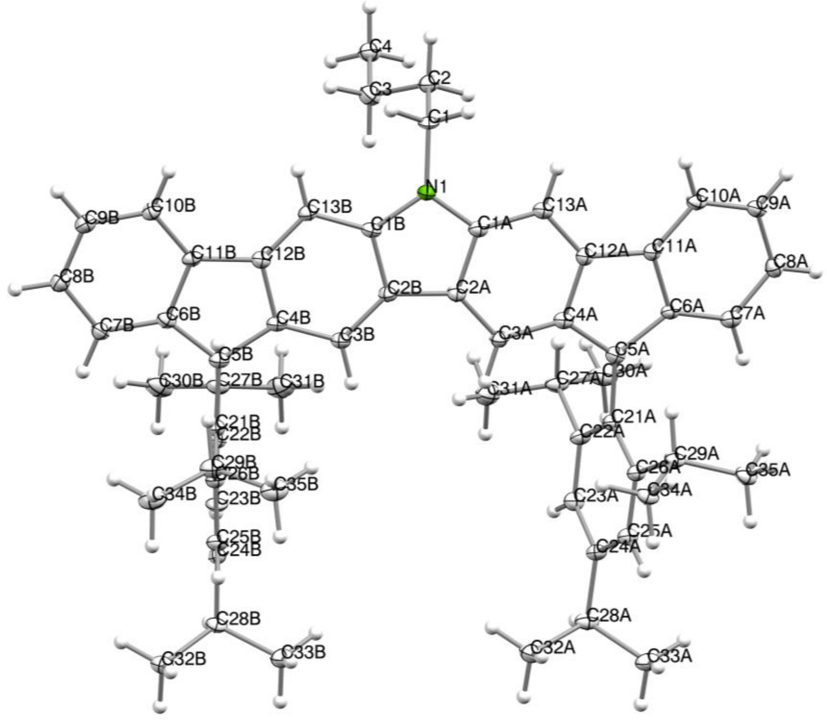

Figure S45. ORTEP plot for **1a**.Thermal ellipsoids are at the 30% probability level.

4.2. NMR Spectroscopy

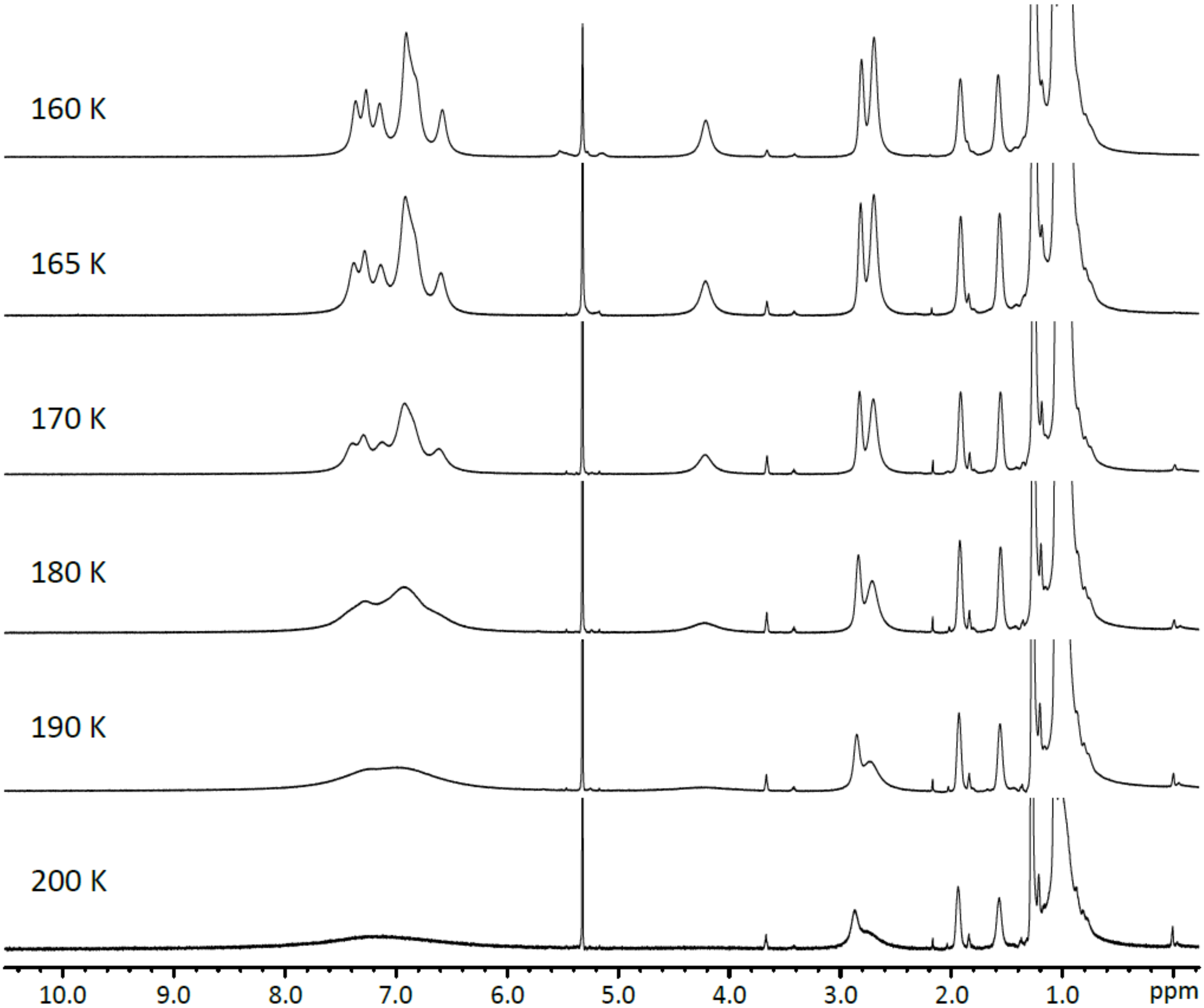

Figure S46. Variable temperature ^{1}H NMR spectra of **1a** (600 MHz, carbon disulfide/dichloromethane-d_2, 200-160 K).

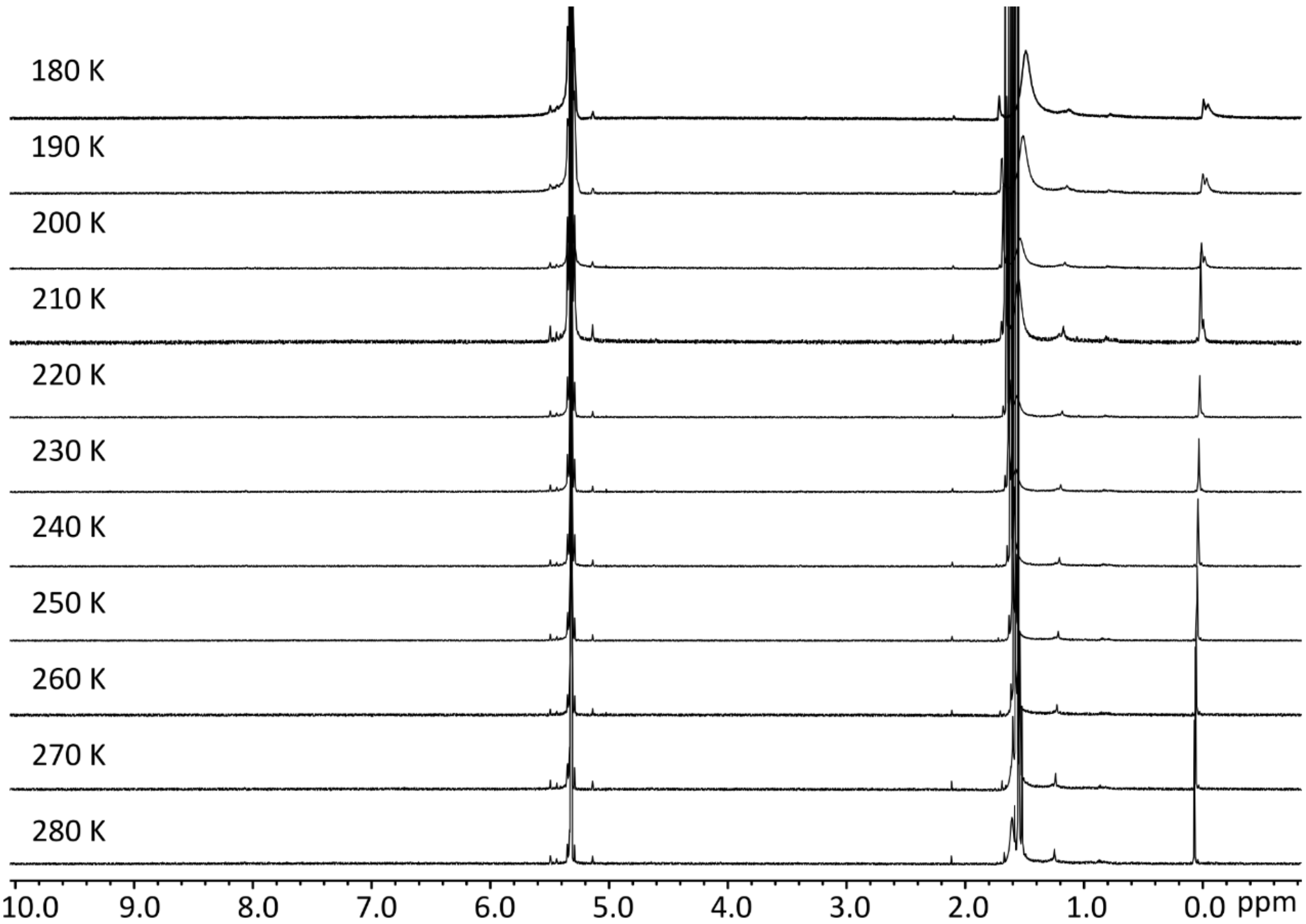

Figure S47. Variable temperature ^{1}H NMR spectra of **1b** (500 MHz, dichloromethane-d_2, 280-180 K).

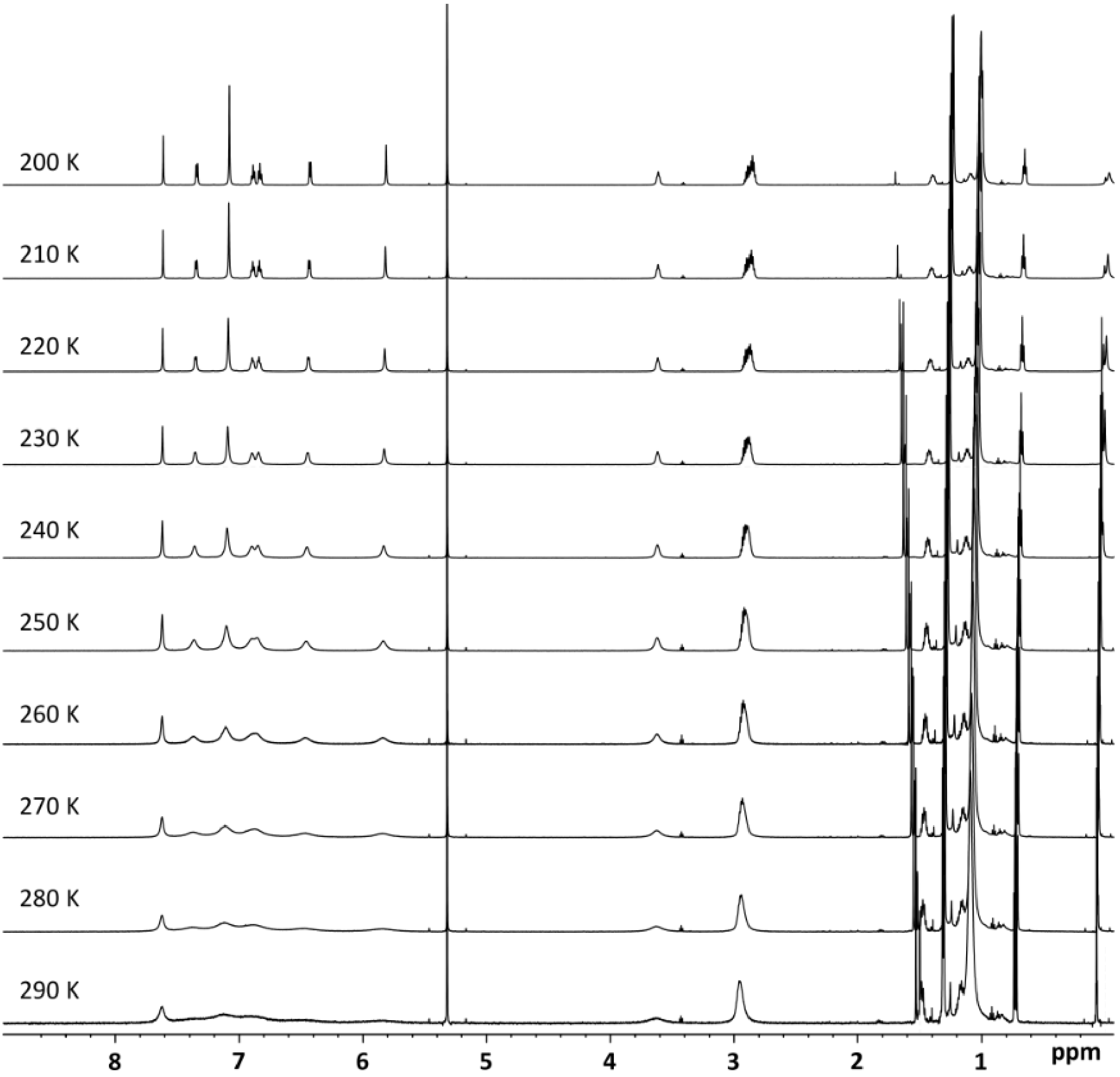

Figure S48. Variable temperature ^{1}H NMR spectra of **2a** (600 MHz, dichloromethane-d_2, 290-200 K).

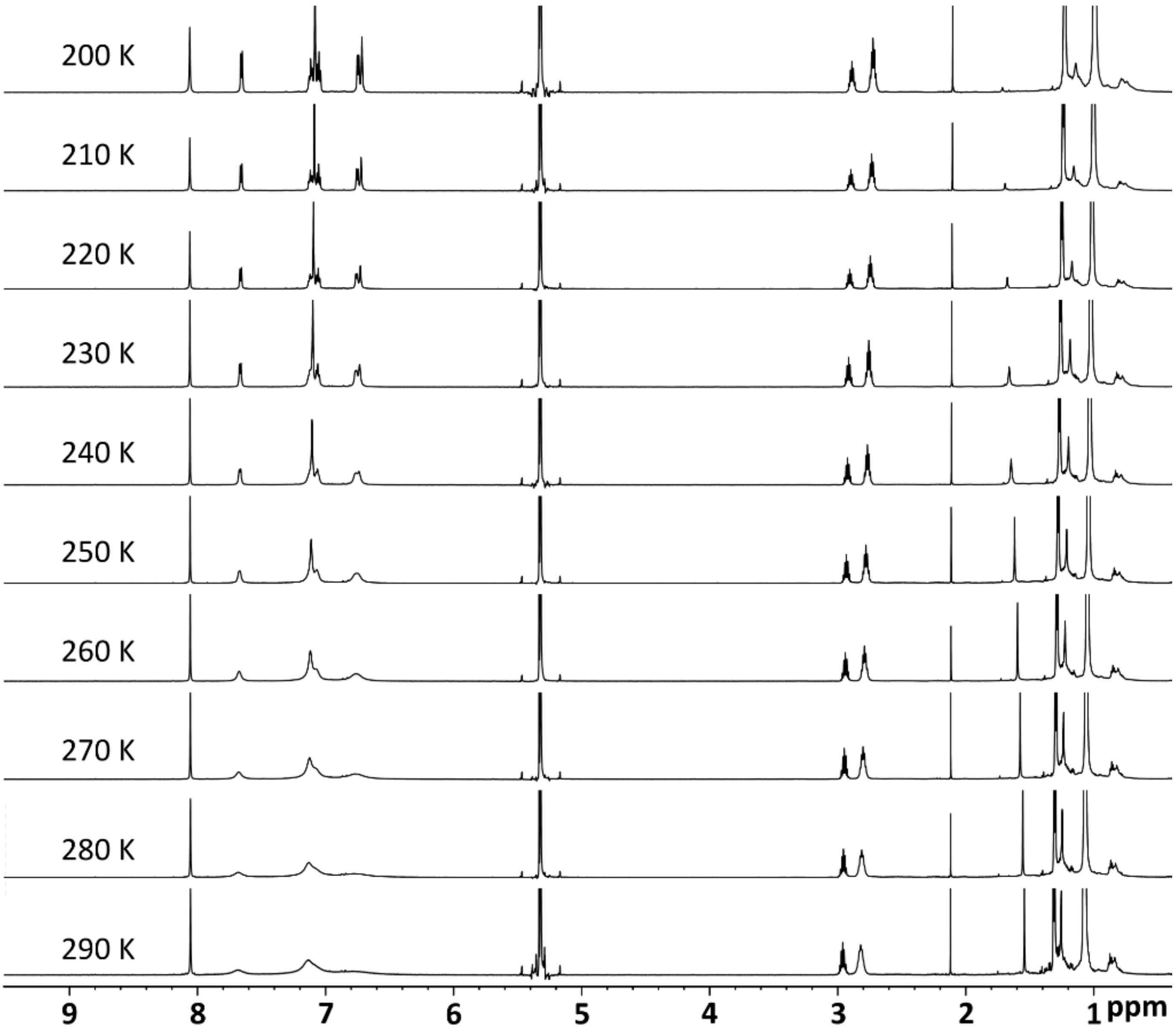

Figure S49. Variable temperature ^{1}H NMR spectra of **2b** (600 MHz, dichloromethane-d_2, 290-200 K).

4.3. ESR Spectroscopy

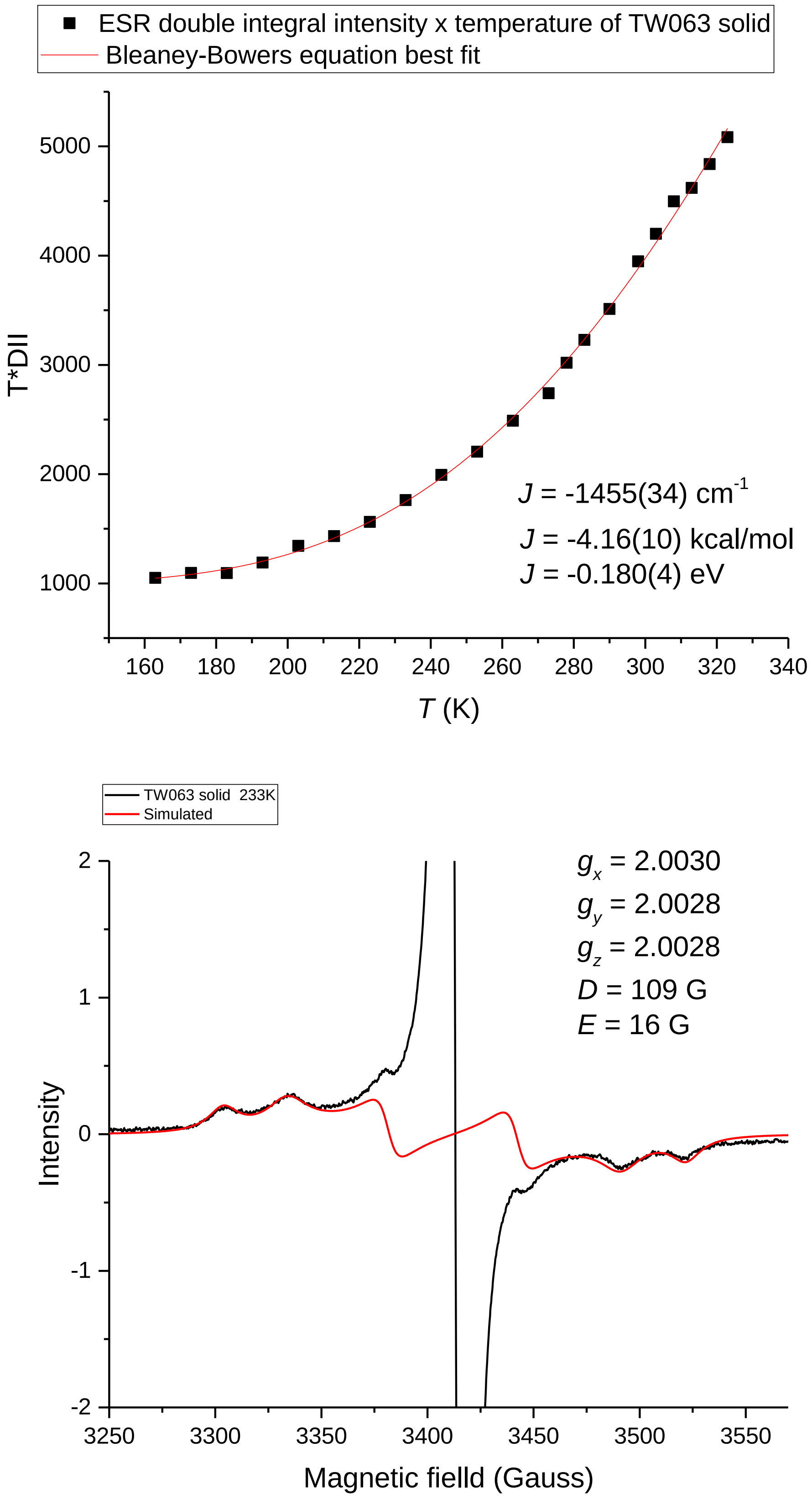

Figure S50. ESR data for **1a** .

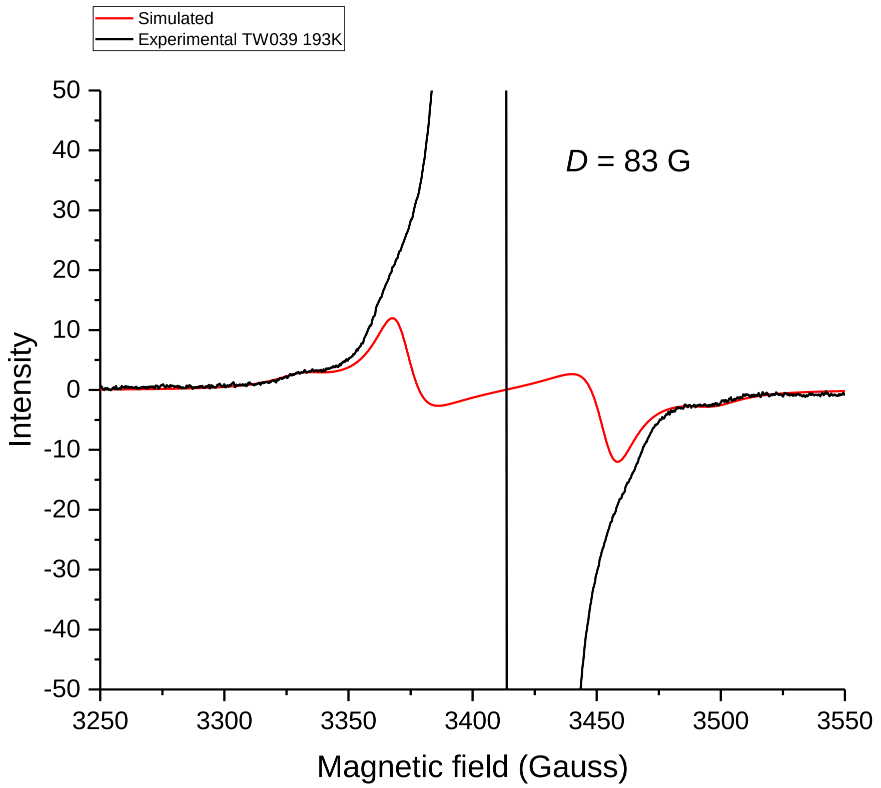

Figure S51. ESR spectrum of **2b**.

4.4. Magnetometry

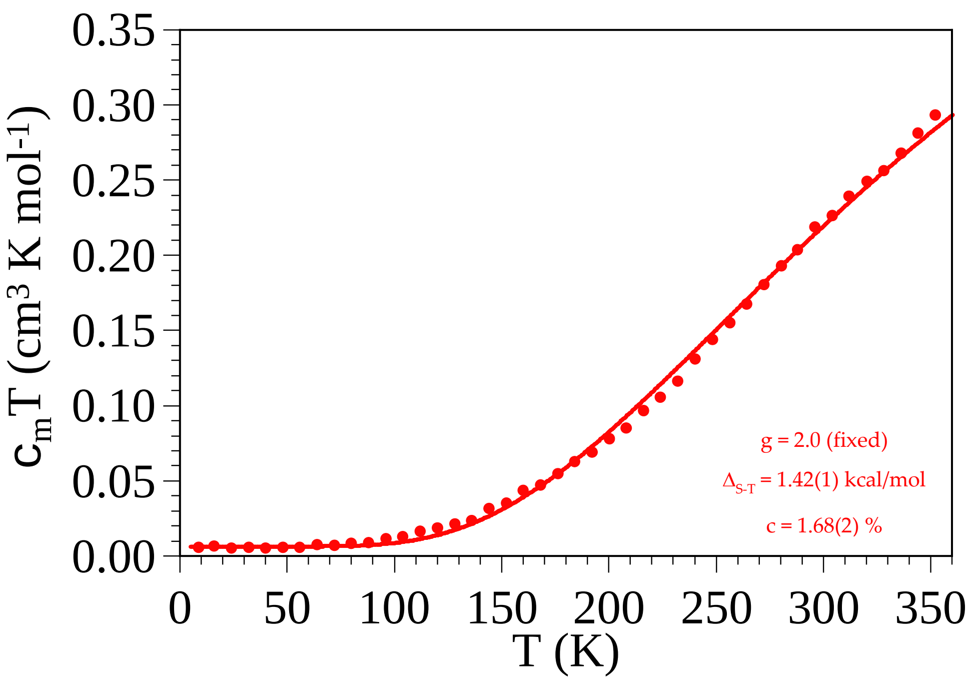

Figure S52. Temperature dependence of magnetic susceptibility of **2b** determined using (SQUID) magnetometry.

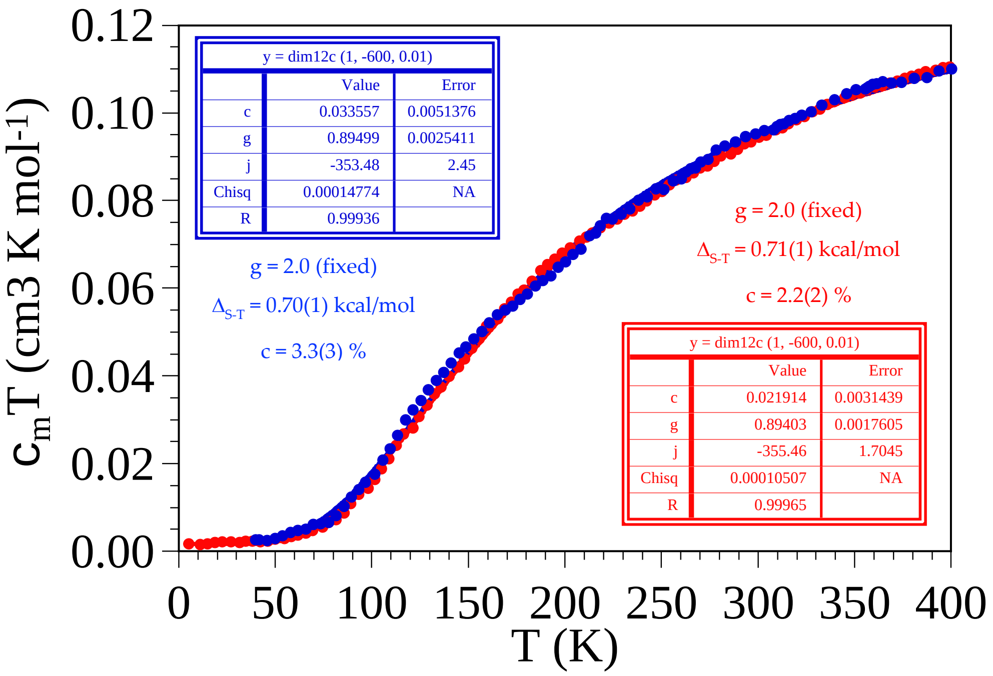

Figure S53. Temperature dependence of magnetic susceptibility of **1b** determined using (SQUID) magnetometry.

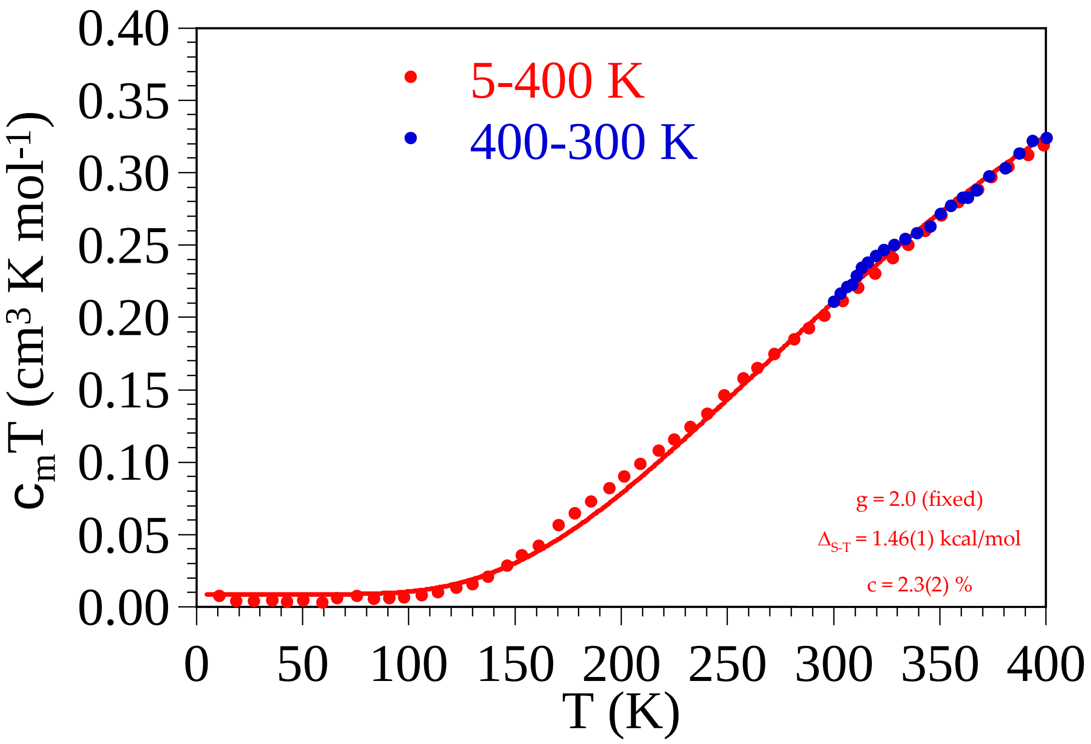

Figure S54. Temperature dependence of magnetic susceptibility of **2a** determined using (SQUID) magnetometry.

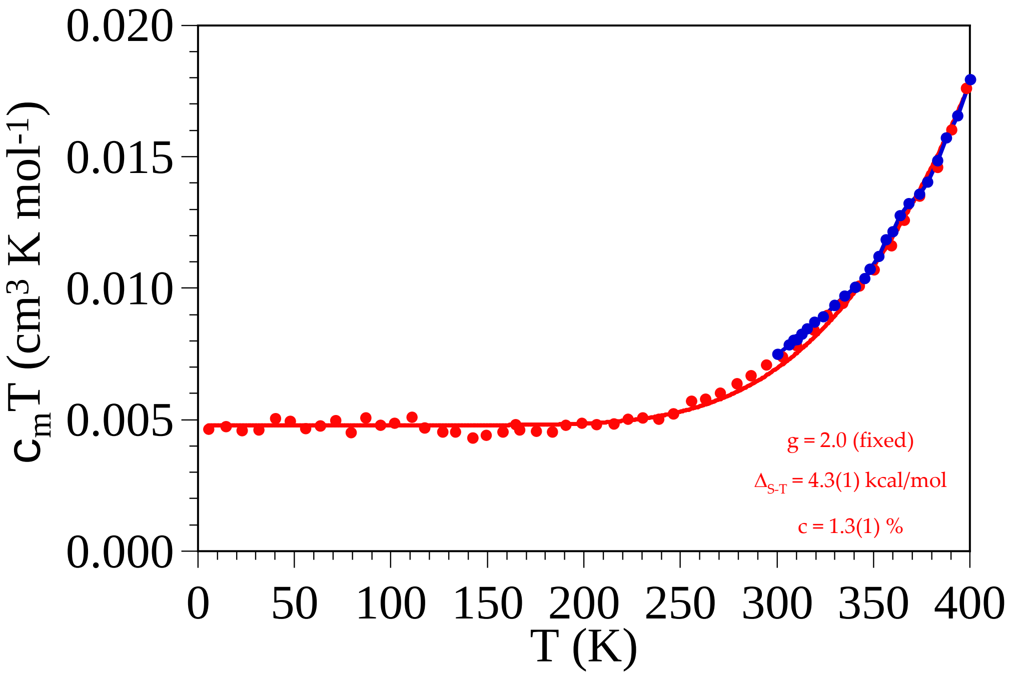

Figure S55. Temperature dependence of magnetic susceptibility of **1a** determined using (SQUID) magnetometry.

4.5. IR Spectroscopy

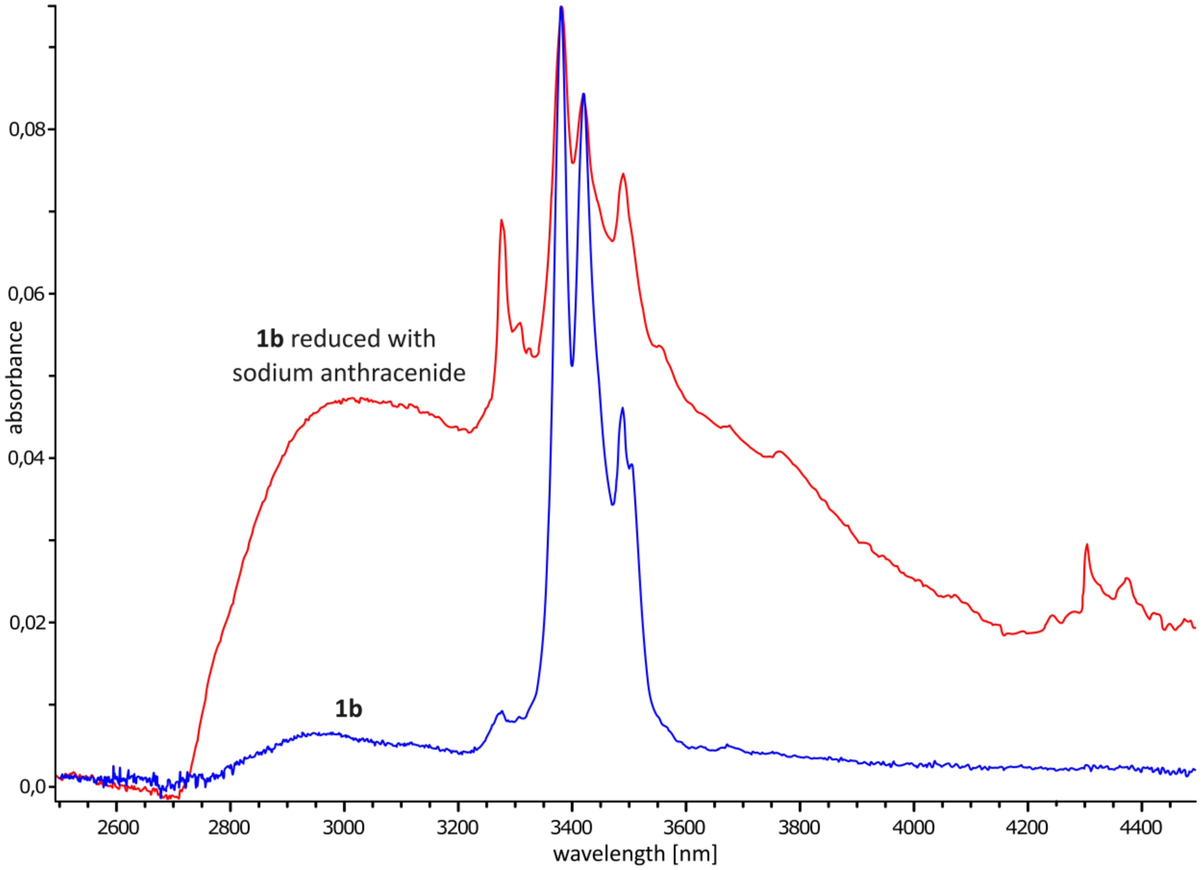

Figure S56. ATR-IR spectra of **1b** (blue trace) and **1b**$^-$ (red trace, made using 3 equiv. of sodium anthracenide in THF, at room temperature, with 30 min. stirring in glovebox + subsequent solvent evaporation). Remaining spectral regions omitted for clarity (no peaks recorded below 2600 nm).

5. Computational Data

5.1. Energies and Geometries

Table S4. Computational data for the DFT optimized structures of difluorenoheteroles.

Code[a]	C[b]	M[c]	SCF E[d,e] a.u.	ZPV[d,f] a.u.	LVF[d,g] cm^{-1}	E[d] a.u.	H[d] a.u.	G[d] a.u.	ΔE_{SCF}[h] kcal/mol	$\langle S^2\rangle$[i] a.a.
**1a'**	2	1	−1053.478056	0.333735	27.17	−1053.126415	−1053.125471	−1053.190252	0.00	
**1a'**	1	2	−1053.806100	0.330819	26.51	−1053.457033	−1053.456089	−1053.522383	1.20	0.772
**1a'**	1	4	−1053.781120	0.330148	24.95	−1053.432813	−1053.431869	−1053.498315	16.87	3.753
**1a'**	0	1	−1054.024729	0.330088	26.24	−1053.676641	−1053.675697	−1053.740397	0.22	0.852
**1a'**	0	3	−1054.022653	0.329879	25.82	−1053.674709	−1053.673765	−1053.739637	1.52	2.005
**1a'**	−1	2	−1054.074806	0.327951	26.51	−1053.728738	−1053.727794	−1053.793253	6.53	0.751
**1a'**	−1	4	−1054.023350	0.325218	24.54	−1053.679618	−1053.678674	−1053.745575	38.82	3.754
**1a'**	−2	1	−1054.032703	0.326254	28.17	−1053.688342	−1053.687398	−1053.752048	3.07	
**5a'**	0	1	−1055.298036	0.356228	25.09	−1054.923444	−1054.922500	−1054.988121	0.00	
**2a'**	2	1	−1053.469274	0.333474	38.08	−1053.117831	−1053.116887	−1053.181802	5.51	
**2a'**	1	2	−1053.808011	0.332071	36.42	−1053.457980	−1053.457036	−1053.522530	0.00	0.788
**2a'**	1	4	−1053.777820	0.328703	37.99	−1053.430663	−1053.429718	−1053.497178	18.95	3.754
**2a'**	0	1	−1054.025078	0.329702	37.39	−1053.677286	−1053.676341	−1053.741269	0.00	0.877
**2a'**	0	3	−1054.022044	0.329871	37.69	−1053.674126	−1053.673182	−1053.739038	1.90	2.005
**2a'**	−1	2	−1054.085214	0.328581	21.39	−1053.738288	−1053.737344	−1053.804004	0.00	0.751
**2a'**	−1	4	−1054.001118	0.322876	30.66	−1053.659290	−1053.658346	−1053.726310	52.77	3.753
**2a'**	−2	1	−1054.037596	0.326213	40.04	−1053.693170	−1053.692226	−1053.757113	0.00	
**8a'**	0	1	−1055.297723	0.356210	37.57	−1054.923164	−1054.922220	−1054.987827	0.20	
**1b'**	2	1	−1396.311193	0.317454	26.69	−1395.975381	−1395.974437	−1396.040468	0.00	
**1b'**	1	2	−1396.641889	0.314811	26.23	−1396.308391	−1396.307447	−1396.375212	5.15	0.773
**1b'**	1	4	−1396.622953	0.314664	24.18	−1396.289835	−1396.288891	−1396.356372	17.04	3.753
**1b'**	0	1	−1396.874198	0.314420	25.85	−1396.541481	−1396.540537	−1396.606247	1.47	0.923
**1b'**	0	3	−1396.873263	0.314423	25.73	−1396.540543	−1396.539598	−1396.606348	2.06	2.006
**1b'**	−1	2	−1396.931482	0.312911	26.13	−1396.600208	−1396.599264	−1396.665678	7.90	0.752
**1b'**	−1	4	−1396.879244	0.309963	25.06	−1396.550558	−1396.549614	−1396.617351	40.68	3.755
**1b'**	−2	1	−1396.902115	0.311354	26.95	−1396.572339	−1396.571395	−1396.637139	0.98	
**5b'**	0	1	−1398.149555	0.340813	24.90	−1397.790176	−1397.789232	−1397.855650	0.00	
**2b'**	2	1	−1396.307546	0.317577	39.80	−1395.971628	−1395.970684	−1396.036724	2.29	
**2b'**	1	2	−1396.650103	0.316525	38.31	−1396.315304	−1396.314360	−1396.380880	0.00	0.778
**2b'**	1	4	−1396.615379	0.313290	40.15	−1396.283394	−1396.282450	−1396.350441	21.79	3.753
**2b'**	0	1	−1396.876542	0.314398	39.96	−1396.543855	−1396.542911	−1396.608649	0.00	0.921
**2b'**	0	3	−1396.872691	0.314456	40.22	−1396.539954	−1396.539009	−1396.605748	2.42	2.005
**2b'**	−1	2	−1396.944070	0.313787	40.41	−1396.612005	−1396.611061	−1396.677357	0.00	0.752
**2b'**	−1	4	−1396.858070	0.307232	37.54	−1396.531724	−1396.530780	−1396.599314	53.97	3.753
**2b'**	−2	1	−1396.903670	0.311386	43.01	−1396.573898	−1396.572953	−1396.638636	0.00	
**8b'**	0	1	−1398.149329	0.340832	40.52	−1397.789941	−1397.788996	−1397.855416	0.14	

[a] structure code as defined in the manuscript. [b] Charge. [c] Multiplicity. Optimized geometries are available as individual files Code_C_M.xyz. [d] CAM-B3LYP/6-31G(d,p). [e] SCF energy. [f] Zero-point vibrational energy. [g] Lowest vibrational frequency. [h] SCF energy relative to the most stable system listed in the table with the same stoichiometry and charge. [j] After annihilation.

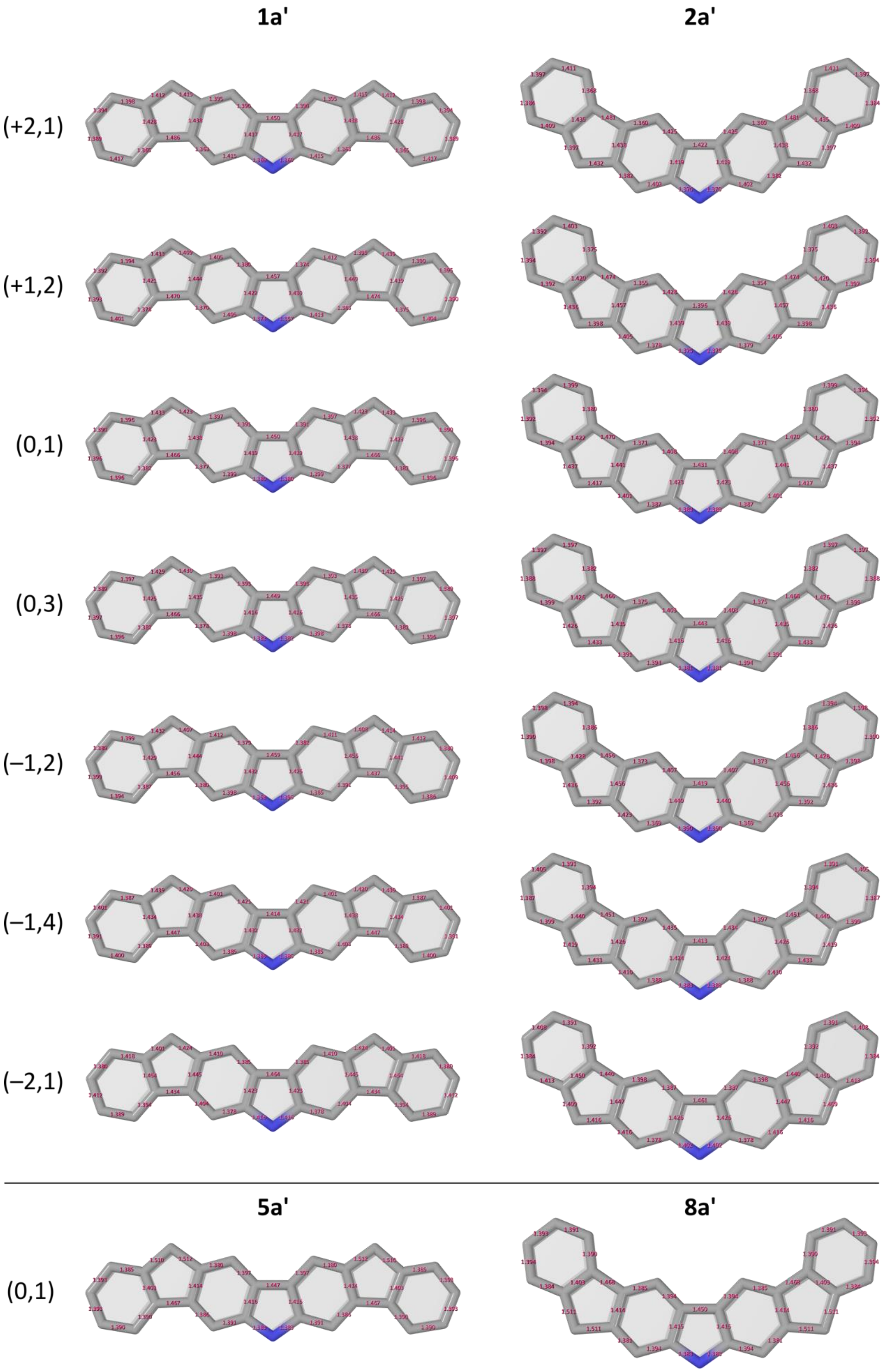

Figure S57. Bond distances in optimized geometries of **1a'**, **2a'**, **5a'**, and **8a'** at the relevant oxidation levels (CAM-B3LYP/6-31G(d,p)).

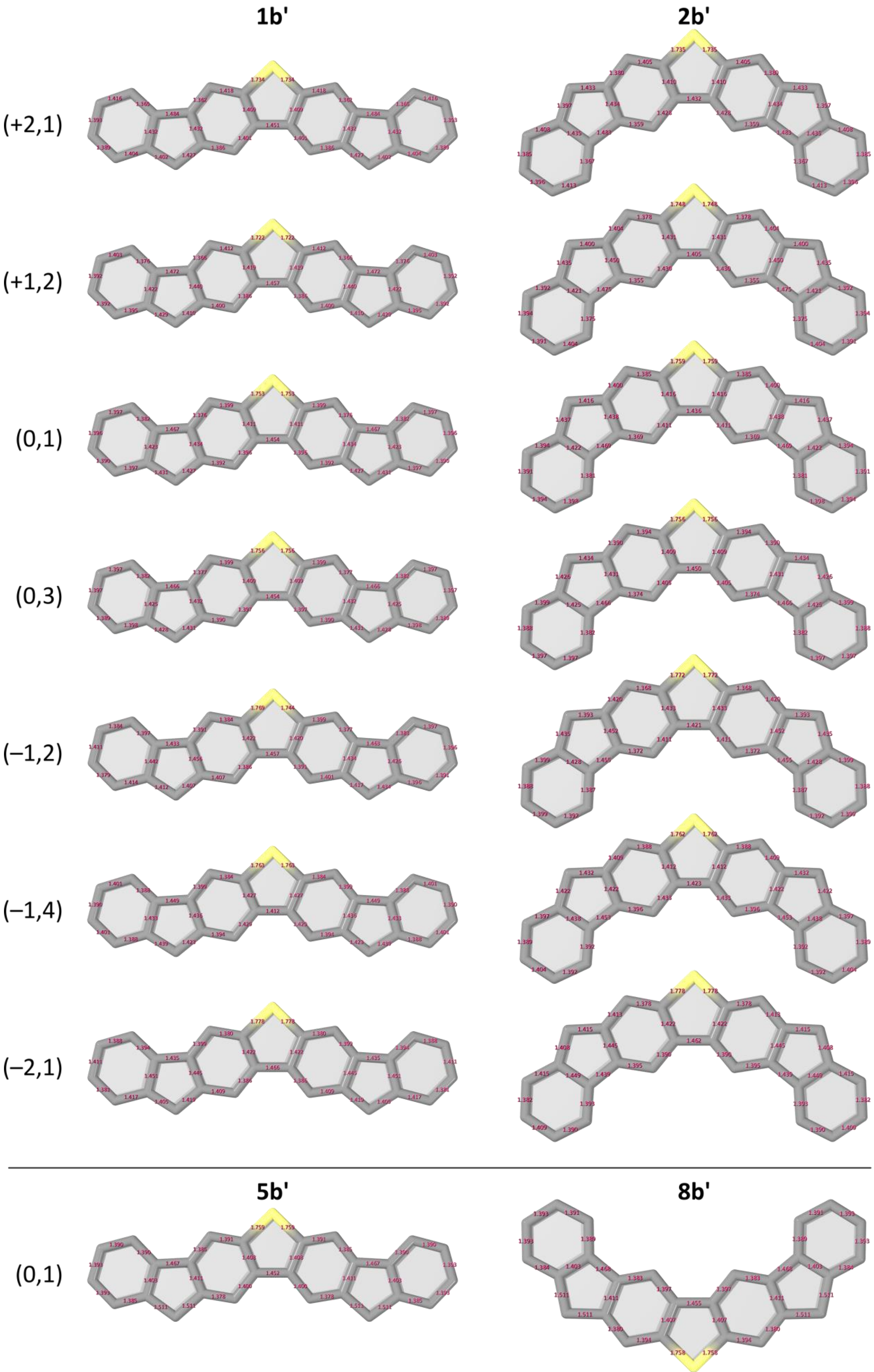

Figure S58. Bond distances in optimized geometries of **1b'**, **2b'**, **5b'**, and **8b'** at the relevant oxidation levels (CAM-B3LYP/6-31G(d,p)).

6. References

(1) Nobuyasu, R. S.; Ward, J. S.; Gibson, J.; Laidlaw, B. A.; Ren, Z.; Data, P.; Batsanov, A. S.; Penfold, T. J.; Bryce, M. R.; Dias, F. B. The Influence of Molecular Geometry on the Efficiency of Thermally Activated Delayed Fluorescence. *J. Mater. Chem. C* **2019**, *7* (22), 6672–6684. https://doi.org/10.1039/C9TC00720B.

(2) Oyama, T.; Mori, T.; Hashimoto, T.; Kamiya, M.; Ichikawa, T.; Komiyama, H.; Yang, Y. S.; Yasuda, T. High-Mobility Regioisomeric Thieno[f,F']Bis[1]Benzothiophenes: Remarkable Effect of Syn/Anti Thiophene Configuration on Optoelectronic Properties, Self-Organization, and Charge-Transport Functions in Organic Transistors. *Advanced Electronic Materials* **2018**, *4* (1), 1700390. https://doi.org/10.1002/aelm.201700390.

(3) Myśliwiec, D.; Stępień, M. The Fold-In Approach to Bowl-Shaped Aromatic Compounds: Synthesis of Chrysaoroles. *Angewandte Chemie International Edition* **2013**, *52* (6), 1713–1717. https://doi.org/10.1002/anie.201208547.

(4) Joseph, V.; Justin Thomas, K. R.; Yang, W. Y.; Kumar Yadav, R. A.; Kumar Dubey, D.; Jou, J.-H. Tetra-Substituted Dipolar Carbazoles: Tuning Optical and Electroluminescence Properties by Linkage Variation. *Asian Journal of Organic Chemistry* **2018**, *7* (8), 1654–1666. https://doi.org/10.1002/ajoc.201800248.

(5) Bain, G. A.; Berry, J. F. Diamagnetic Corrections and Pascal's Constants. *J. Chem. Educ.* **2008**, *85* (4), 532. https://doi.org/10.1021/ed085p532.

(6) Frisch, M. J.; Trucks, G. W.; Schlegel, H. B.; Scuseria, G. E.; Robb, M. A.; Cheeseman, J. R.; Scalmani, G.; Barone, V.; Petersson, G. A.; Nakatsuji, H.; Li, X.; Caricato, M.; Izmaylov, A. F.; Zheng, G.; Sonnenberg, J. L.; Hada, M.; Ehara, M.; Toyota, K.; Fukuda, R.; Hasegawa, J.; Ishida, M.; Nakajima, T.; Honda, Y.; Kitao, O.; Nakai, H.; Vreven, T.; Montgomery, Jr., J. A.; Peralta, J. E.; Ogliaro, F.; Bearpark, M.; Heyd, J. J.; Brothers, E.; Kudin, K. N.; Staroverov, V. N.; Kobayashi, R.; Normand, J.; Raghavachari, K.; Rendell, A.; Burant, J. C.; Iyengar, S. S.; Tomasi, J.; Cossi, M.; Millam, J. M.; Klene, M.; Adamo, C.; Gomperts, R.; Stratmann, R. E.; Yazyev, O.; Austin, A. J.; Cammi, R.; Pomelli, C.; Ochterski, J. W.; Martin, R. L.; Morokuma, K.; Zakrzewski, V. G.; Voth, G. A.; Salvador, P.; Dannenberg, J. J.; Dapprich, S.; Daniels, A. D.; Farkas, O.; Foresman, J. B.; Fox, D. J. Gaussian 16, Revision B.01, 2016.

(7) Becke, A. D. Density-Functional Exchange-Energy Approximation with Correct Asymptotic Behavior. *Phys. Rev., A* **1988**, *38* (6), 3098–3100.

(8) Becke, A. D. Density-functional Thermochemistry. III. The Role of Exact Exchange. *J. Chem. Phys.* **1993**, *98* (7), 5648–5652. https://doi.org/10.1063/1.464913.

(9) Lee, C.; Yang, W.; Parr, R. G. Development of the Colle-Salvetti Correlation-Energy Formula into a Functional of the Electron Density. *Phys. Rev. B* **1988**, *37* (2), 785–789. https://doi.org/10.1103/PhysRevB.37.785.

(10) Yanai, T.; Tew, D. P.; Handy, N. C. A New Hybrid Exchange–Correlation Functional Using the Coulomb-Attenuating Method (CAM-B3LYP). *Chemical Physics Letters* **2004**, *393* (1–3), 51–57. https://doi.org/10.1016/j.cplett.2004.06.011.

(11) Grimme, S.; Ehrlich, S.; Goerigk, L. Effect of the Damping Function in Dispersion Corrected Density Functional Theory. *Journal of Computational Chemistry* **2011**, *32* (7), 1456–1465. https://doi.org/10.1002/jcc.21759.

(12) Dunning, T. H. Gaussian Basis Sets for Use in Correlated Molecular Calculations. I. The Atoms Boron through Neon and Hydrogen. *J. Chem. Phys.* **1989**, *90*, 1007–1023. https://doi.org/10.1063/1.456153.

(13) Zimmerman, P. M.; Bell, F.; Goldey, M.; Bell, A. T.; Head-Gordon, M. Restricted Active Space Spin-Flip Configuration Interaction: Theory and Examples for Multiple Spin Flips with Odd Numbers of Electrons. *The Journal of Chemical Physics* **2012**, *137* (16), 164110. https://doi.org/10.1063/1.4759076.

(14) Bell, F.; Zimmerman, P. M.; Casanova, D.; Goldey, M.; Head-Gordon, M. Restricted Active Space Spin-Flip (RAS-SF) with Arbitrary Number of Spin-Flips. *Phys. Chem. Chem. Phys.* **2013**, *15* (1), 358–366. https://doi.org/10.1039/C2CP43293E.

(15) Casanova, D. Short-Range Density Functional Correlation within the Restricted Active Space CI Method. *The Journal of Chemical Physics* **2018**, *148* (12), 124118. https://doi.org/10.1063/1.5018895.

(16) Kruszewski, J.; Krygowski, T. M. Definition of Aromaticity Basing on the Harmonic Oscillator Model. *Tetrahedron Letters* **1972**, *13* (36), 3839–3842. https://doi.org/10.1016/S0040-4039(01)94175-9.

(17) Krygowski, T. M.; Szatylowicz, H.; Stasyuk, O. A.; Dominikowska, J.; Palusiak, M. Aromaticity from the Viewpoint of Molecular Geometry: Application to Planar Systems. *Chem. Rev.* **2014**, *114* (12), 6383–6422. https://doi.org/10.1021/cr400252h.

(18) Nakano, M. Electronic Structure of Open-Shell Singlet Molecules: Diradical Character Viewpoint. *Top Curr Chem (Z)* **2017**, *375* (2), 47. https://doi.org/10.1007/s41061-017-0134-7.
